# Ivermectin repurposing for COVID-19: pharmacological and bibliometric analysis

**DOI:** 10.1007/s00210-025-04233-5

**Published:** 2025-05-06

**Authors:** Maresa Dulle, Roland Seifert

**Affiliations:** Institute of Pharmacology, Hannover Medical School, Carl-Neuberg-Str. 1, D-30625 Hannover, Germany

**Keywords:** COVID-19, SARS-CoV-2, Ivermectin, Drug repurposing, Antiviral treatment, Methodological bias, Preprints, Citation analysis, Pandemic, Evidence-based medicine

## Abstract

Since the onset of the COVID-19 pandemic in March 2020, researchers worldwide have sought effective drugs to prevent and manage SARS-CoV-2 and its spectrum of symptoms. Ivermectin, originally developed as an anthelmintic for controlling parasitic infections in humans and animals, has drawn attention based on the hypothesis that it inhibits viral replication. In Austria, ivermectin usage peaked in November 2021, following promotion by the right-wing Freedom Party of Austria (FPÖ) as an alternative treatment to vaccination, resonating strongly within anti-vaccine and skeptical communities. The topic is also very present in the United States of America due to the re-election of D. Trump as US President and the designation of R. Kennedy as the United States’ Secretary of Health and Human Services. To critically examine the controversial use of ivermectin for COVID-19 and publication trends during the pandemic, this study analysed all publications listed in PubMed from 1 January 2020 to 31 December 2022 using the keywords ‘ivermectin’ and ‘COVID-19’, resulting in a dataset of 353 publications. These publications were assessed for scientific quality, methodological rigour and bias, with particular focus on the influence of social and political dynamics on publication practices, as well as the prevalence of preprints, citation trends and the role of funding sources. Our study shows that many highly cited studies on ivermectin display methodological weaknesses and data gaps, contributing to the propagation of hypotheses lacking substantial empirical support. This analysis underscores the necessity of rigorous quality control during crises and highlights the long-term risks posed to scientific databases and public health by methodologically deficient research.

**Supplementary Information:**

The online version contains supplementary material available at 10.1007/s00210-025-04233-5.

## Introduction

Since the outbreak of the COVID-19 pandemic in March 2020, researchers worldwide have been looking for a drug to prevent and combat the SARS-CoV-2 coronavirus and its range of symptoms. One drug that has been in focus is the anthelmintic ivermectin. From 2020 to 2021, the number of publications in the PubMed database doubled (https://pubmed.ncbi.nlm.nih.gov/?term=ivermectin; accessed November 17, 2024). Ivermectin belongs to the group of avermectins. These are fermentation products of the bacterium *Streptomyces avermectinus*, which belong to the macrocyclic lactones. Avermectin B1a and B1b reduced to dihydroxy derivatives showed the greatest anthelmintic efficacy (Crump and Otoguro 2005). The discovery of these substances is largely due to the work of Satoshi Ōmura, who was awarded the Nobel Prize for Medicine in 2015 for his outstanding contributions (https://www.leopoldina.org/presse-1/nachrichten/satoshi-omura-erhaelt-medizin-nobelpreis-2015/ last accessed 05/22/2024). Ivermectin is used as an ecto- and endoparasiticide. It has an effect against nematodes and arthropods. This effect is based on the binding of ivermectin to glutamate-gated chloride channels and to GABA receptors in the nerve cells of the parasites. This binding causes hyperpolarisation of the cell membranes, leading to the death of the parasites (Fox 2006). In veterinary medicine, it is used in particular in cattle, horses, sheep and swines (Barragry 1987). In the field of human medicine, ivermectin is used to treat various parasitic diseases such as scabies, pediculosis, myiasis, loiasis, onchocerciasis, rosacea and strongyloidiasis (Johnson-Arbor 2022). In addition to its antiparasitic effect, ivermectin also has anti-carcinogenic (Sharmeen et al. 2010), anti-inflammatory and antiviral properties (Laing et al. 2017). In vitro studies report that ivermectin acts as a DNA helicase inhibitor in several viruses, including BK polyomavirus (Bennett et al. 2015), pseudorabies virus (Lv et al. 2018), porcine circovirus 1 (Wang et al. 2019) and bovine herpesvirus 1 (Raza et al. 2020). Furthermore, in vitro, ivermectin inhibits the replication of HIV by inhibiting the importin-α/β−1 complex and the RNA helicase in viruses such as dengue fever (Tay et al. 2013), yellow fever (Mastrangelo et al. 2012), West Nile virus (Mastrangelo et al. 2012) and Venezuelan equine encephalitis (Shechter et al. 2017). There are also in vitro studies confirming the efficacy of ivermectin as an inhibitor of HIV replication by inhibiting importin α/β1 (Jans et al. 2019). However, since ivermectin is not approved for this use as a drug, such use is referred to as repurposing.

Repurposing drugs means ‘studying the drugs that are already approved to treat one disease or condition to see if they are safe and effective for treating other diseases’ (Sonaye et al. 2021). Repurposing offers numerous advantages, particularly in terms of efficiency and safety. One major advantage is the reduced time required, since a large pool of existing research can be drawn upon. This enables a significantly shorter time span between the identification of a new therapeutic use and the practical application of the drug. In addition, research costs are usually lower because the basic pharmacological properties of the drug have already been investigated. Another significant advantage is the lower risk of adverse effects, since the safety of the drug has already been extensively researched and proven in previous applications. These factors make repurposing an attractive strategy, especially in situations where fast and safe solutions are needed (Pushpakom et al. 2019). During the COVID-19 pandemic, the hypothesis developed that ivermectin has an antiviral effect against the SARS-CoV-2 virus (Caly et al. 2020). This hypothesis is based on the fact that SARS-CoV-2, as a positive-stranded ssRNA virus, uses importin α/β1 for transport in the cell. Caly et al. (2020) conducted in vitro experiments with COVID-19-infected Vero/hSLAM cells, in which ivermectin at a concentration of 5 µM was administered over a period of 3 days and a 5000-fold reduction in virus was observed after 48 h. This is countered by different publications e.g. Schmith et al. who stated that the concentration required in Caly et al. was 35 times higher than the maximum plasma concentration (*C*_max_) of the approved preparation, which indicates an excessive dosage (Schmith et al. 2020).

Right-wing parties are using the data by Caly et al. (2020) to promote ivermectin as a potential COVID-19 prophylaxis or therapy. In Germany, the Alternative for Germany (AfD) represents an opposition party (Stern 2021). It submitted requests to the government to promote research into ivermectin and criticise the use of other drugs. In December 2021, the right-wing populist politician of the Freedom Party of Austria (FPÖ), Herbert Kickl, recommended the use of ivermectin as an alternative to vaccinations against COVID-19 in an interview (https://www.derstandard.de/story/2000132,207,743/kickl-wettert-gegen-impfung-und-fordert-studien-mit-ivermectin, last accessed on 24 May 2024). This happened despite warnings and advice from international health authorities: The FDA advised against the use of ivermectin outside clinical trials on 5 March 2021 (/https://www.cnn.com/2021/03/05/health/ivermectin-covid-19-fda-statement-wellness/index.html, last accessed on 16/06/2024), the EMA on 22 March 2021 (https://www.ema.europa.eu/en/news/ema-advises-against-use-ivermectin-prevention-or-treatment-covid-19-outside-randomised-clinical-trials, last accessed on 16/06/2024) and the WHO on 31 March 2021 (https://www.who.int/news-room/feature-stories/detail/who-advises-that-ivermectin-only-be-used-to-treat-covid-19-within-clinical-trials, last accessed on 16/06/2024). The topic is still relevant today. In the news satire ‘Heute-Show’ on the public broadcaster ZDF on 10 April 2024, the election victory of the FPÖ with its recommendation to take ivermectin against COVID-19 was reported again even if the formation of a government with Kickl as potential chancellor subsequently failed (https://www.zdf.de/comedy/heute–show/heute–show–vom–4–oktober–2024–100.html, last accessed on 26 October 2024; https://www.tagesschau.de/ausland/europa/oesterreich-koalition-bestaetigung-100.html, last accessed 04/11/2025).

The topic also remains highly relevant in the USA due to Donald Trump’s victory in the presidential election. Despite the open letter written by 77 Nobel Laureates on 10 December as part of Nobel Day, in which they spoke out against the selection of R. Kennedy, he was sworn in as the United States’ Secretary of Health and Human Services (https://www.nytimes.com/2024/12/09/health/kennedy–hhs–nobel–laureates.html, last accessed 12/20/2024; https://www.hhs.gov/about/leadership/index.html, last accessed 04/11/2025). The debate shows how strongly health policy is interwoven with global power relations, trust in institutions and ideologies. Table 1 presents key opinions of the political ivermectin debate.

**Table 1:** Statements by various parties and public figures on the ivermectin issue

Parties/persons	Statement	Sources
H. Kickl (FPÖ)	12/2021: recommended the use of ivermectin as an alternative to vaccinations against COVID-19	https://www.derstandard.de/story/2000132,207,743/kickl-wettert-gegen-impfung-und-fordert-studien-mit-ivermectin, last accessed 05/24/2024
AfD	12/18/2020: minor question with 19 questions to the German government which lists many studies as arguments for the effectiveness of ivermectin against COVID-19	(Weidel et al. 2021)
	06/14/2021: twelve-question request to the Bavarian state parliament regarding the use of ivermectin as an alternative to COVID-19 vaccination	(Klingen 2021)
R. Paul	8/31/2021: ‘The hatred for Trump deranged these people so much, that they’re unwilling to objectively study it. So someone like me that’s in the middle on it, I can’t tell you because they will not study ivermectin. They will not study hydroxychloroquine without the taint of their hatred for Donald Trump’	https://edition.cnn.com/2021/08/31/politics/rand-paul-covid-19-ivermectin/index.html, last accessed 12/30/2024
F. Kennedy	10/25/2024: ‘FDA’s war on public health is about to end. This includes its aggressive suppression of psychedelics, peptides, stem cells, raw milk, hyperbaric therapies, chelating compounds, ivermectin, hydroxychloroquine, vitamins, clean foods, sunshine, exercise, nutraceuticals and anything else that advances human health and can’t be patented by Pharma. […]’	https://x.com/RobertKennedyJr/status/1849925311586238737, last accessed 11/18/2024
Nobel laureates	12/10/2024: ‘We, the undersigned Nobel Laureates, are writing to ask you to oppose the confirmation of Robert F. Kennedy, Jr. as Secretary of the Department of Health and Human Services (DHHS). […]’	https://www.nytimes.com/interactive/2024/12/09/health/rfkltr.html, last accessed 12/20/2024

To examine the data basis behind this tension between political propaganda and science, we performed a pharmacological and bibliometric analysis of the use of ivermectin for the prevention and treatment of COVID-19 as well as the publication behaviour during the COVID-19 pandemic.

## Material and methods

**Fig. 1:**
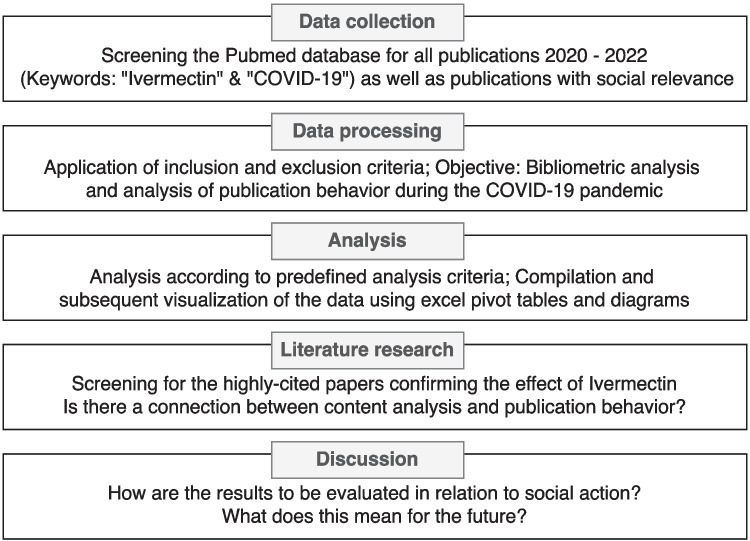
This flowchart shows an overview of our method

**Fig. 2:**
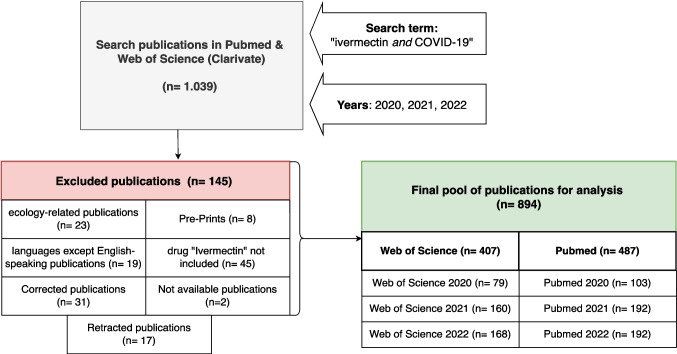
Detailed overview of exclusion and inclusion criteria

Figure 1 shows the methodological approach, which is explained in detail below. A bibliometric analysis was conducted of all English-language publications from 2020, 2021 and 2022 that were indexed in the PubMed and Web of Science (Clarivate) databases with the keywords ‘ivermectin’ and ‘COVID-19’ (*n* = 1039). Only publications in which ivermectin is mentioned as a drug were included. If ivermectin is not explicitly mentioned, the publication was excluded (*n* = 45). In addition, publications in other languages (*n* = 19), preprints (*n* = 8), unavailable full texts (*n* = 2), corrected (*n* = 31) and withdrawn publications (*n* = 17) and studies on the environmental effects of ivermectin (*n* = 23) were excluded. The final pool of publications for the analysis comprises 894 publications, of which 487 are from PubMed and 407 from Web of Science. Figure 2 shows this breakdown of the exclusion criteria and the publications included.

After applying the exclusion criteria, each publication was analysed individually. The following criteria were defined for this purpose: Country of publication, number and type of drugs mentioned, type of document type published, inclusion of preprints in the sources, frequency of citation, authors’ opinion on the efficacy of ivermectin and the latency period between submission and publication with significance testing using ANOVA analysis. The data were taken from the online versions of the publications or the PubMed and Web of Science databases. Most publications were published in Brazil, India and the USA. Therefore, only these three countries were considered in country-specific analyses. To show the number of all named drugs, the drugs were categorised according to their drug group. The total number of mentions of each drug was calculated and, consequently, a statement was made about the distribution of the individual drugs within all publications.

To evaluate the different types of document types based on their scientific value, a category system was introduced and the document types were assigned to three different categories (1–3) (Table 1). The document type ‘study’ is an inhomogeneous group with many different study biases. These cannot be clearly assigned to one category and thus, were excluded from the category one to three to form the category ‘study’. To compare the citation numbers with the COVID-19 case numbers, the citations of the publications from PubMed were collected using Semantic Scholar. For publications from Web of Science, the data was taken directly from the database. The worldwide monthly case numbers 2020–2023 were taken from the WHO COVID-19 dashboard.

A subsequent literature search was conducted to analyse publication behaviour. For this purpose, all publications were analysed in terms of content and the arguments justifying the prescription and use of ivermectin were filtered. We classified these on the basis of their validity and analysed in detail the arguments that were not valid. Looking at the number of citations, seven publications in the PubMed database and four publications in the Web of Science database were cited significantly more often than the rest (< 95 citations). The content analysis of these publications for quality assessment is based on the following criteria: objective, study design, risk of bias, data availability, statistical methodology, result, conclusion, conflict of interest and reliability of sources. In addition, the quality assessment was clearly displayed in colour using a traffic light system.

To visualise the results, graphics and diagrams were created using Microsoft Excel based on the statistical analysis of the Microsoft Excel pivot tables.

This analysis includes publications from the databases PubMed and Web of Science. A comparison of the structure of the two databases is shown in Table 3. Comparing the results of both databases, they are relatively homogeneous overall (Table 4). Therefore, in this publication we will focus on the results of the PubMed database, which are presented in Figs. 3, 4, 5, 6, 7, 8, 9, 10, 11, 12, 13 and Tables 1, 2, 3, 4, 5, 6, 7, as well as Supplemental Tables 2, 3, 4 and 8. The results of the Web of Science data analysis can be found in the appendix, including Supplement Figs. 1, 2, 3, 4, 5, 6, 7, 8, 9, 10, 11, 12 and 13 and Supplement Tables 1 and 5, 6 and 7. The methodological process is summarised in Figs. 1 and 2.

**Fig. 3:**
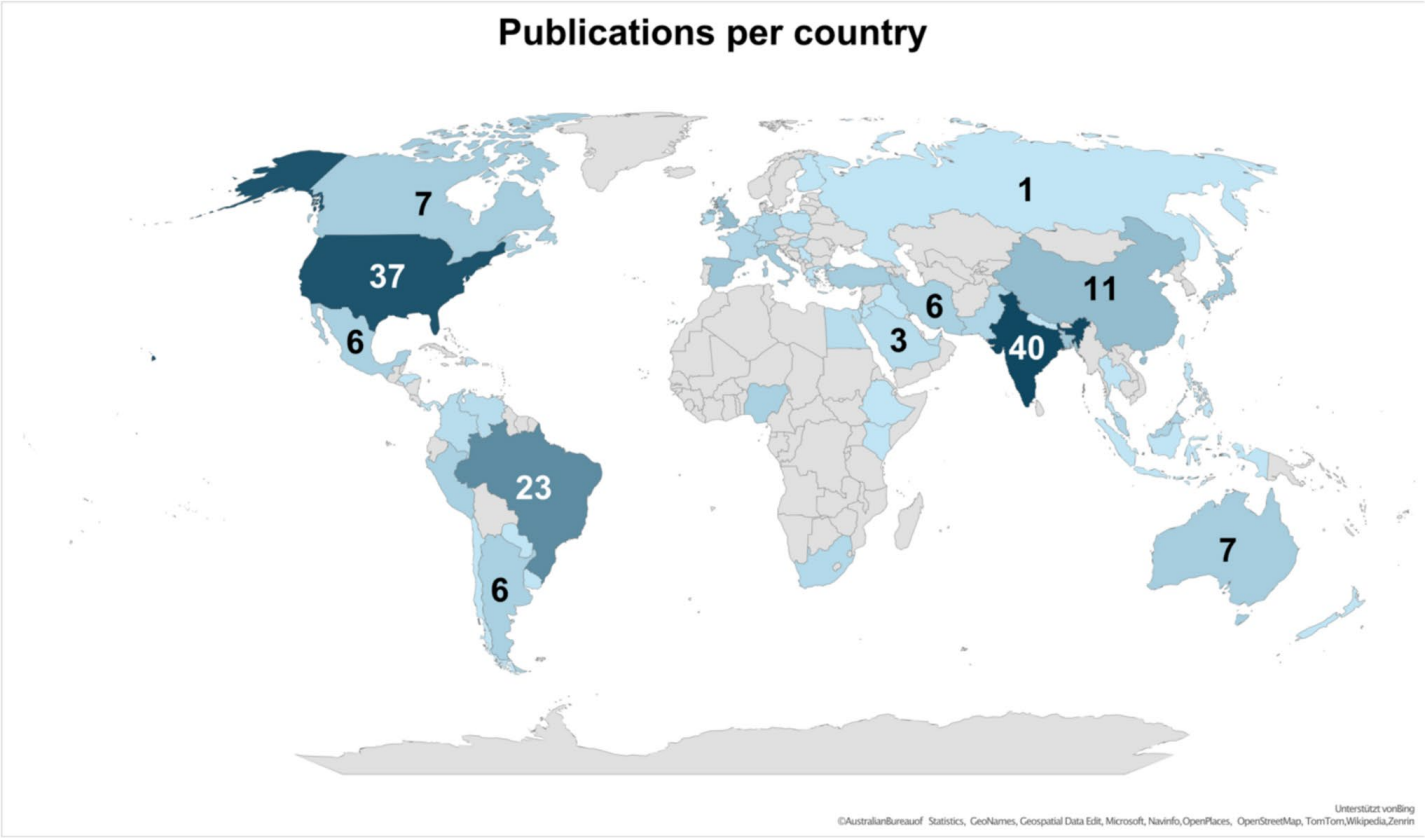
The world map shows the number of publications per country in 2020, 2021 and 2022 and highlights the three countries with the most publications in white

**Fig. 4:**
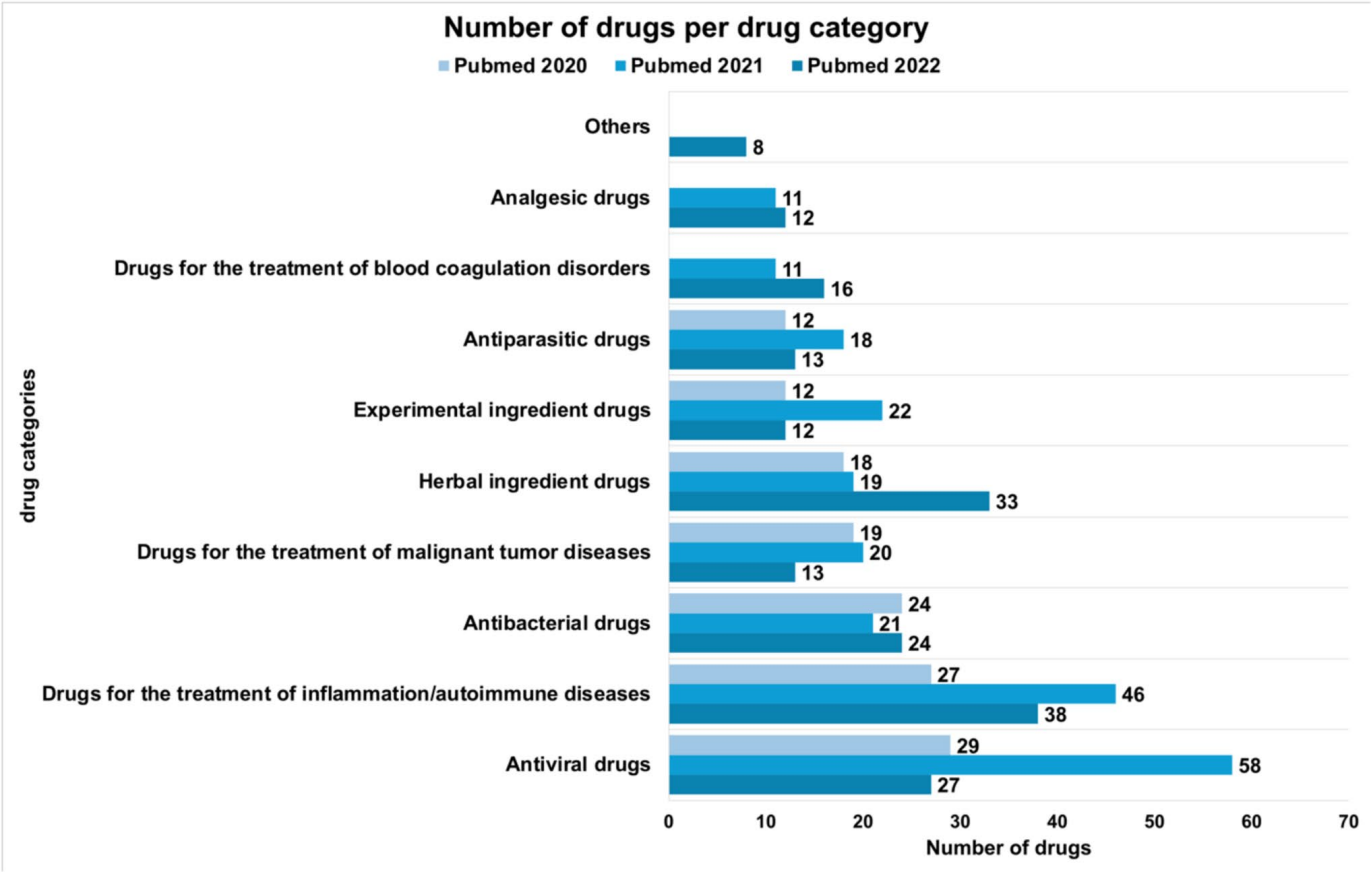
The chart shows the absolute number of drugs per drug category in the years 2020, 2021 and 2022 mentioned in all publications

**Fig. 5:**
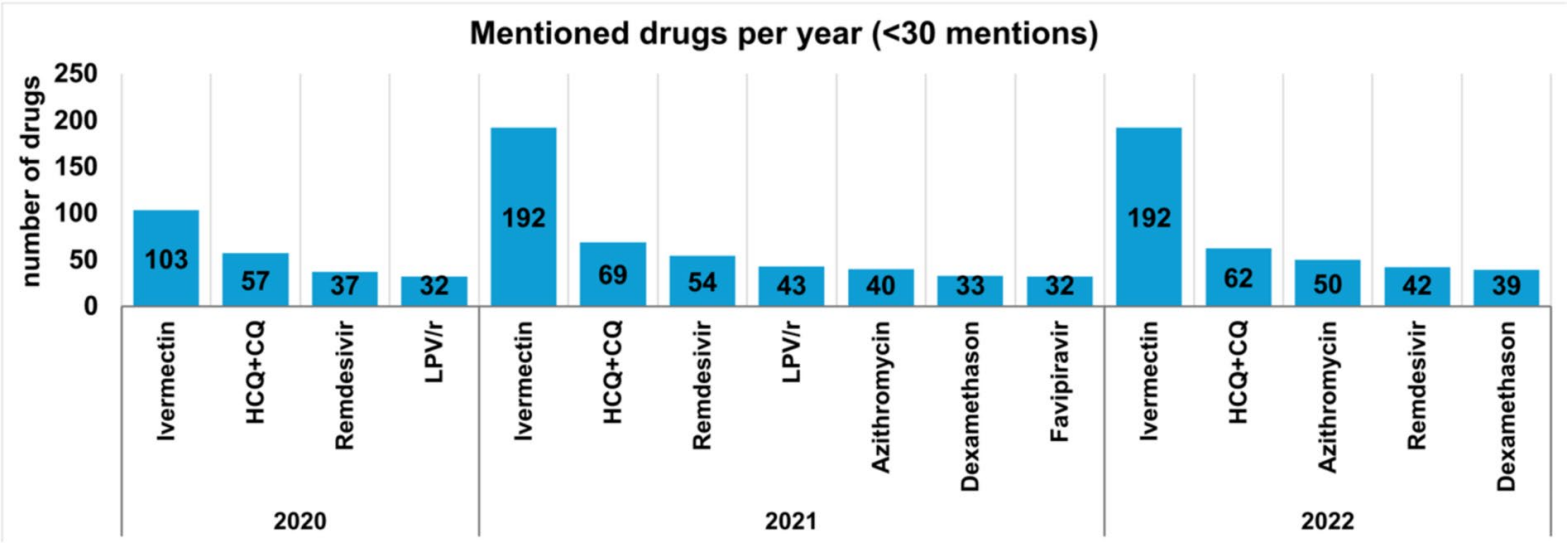
Looking at each drug individually, this graph shows the total number of mentions of each drug in the years 2020, 2021 and 2022. Only drugs mentioned more than 30 times are included. The y-axis shows the number of drugs

**Fig. 6:**
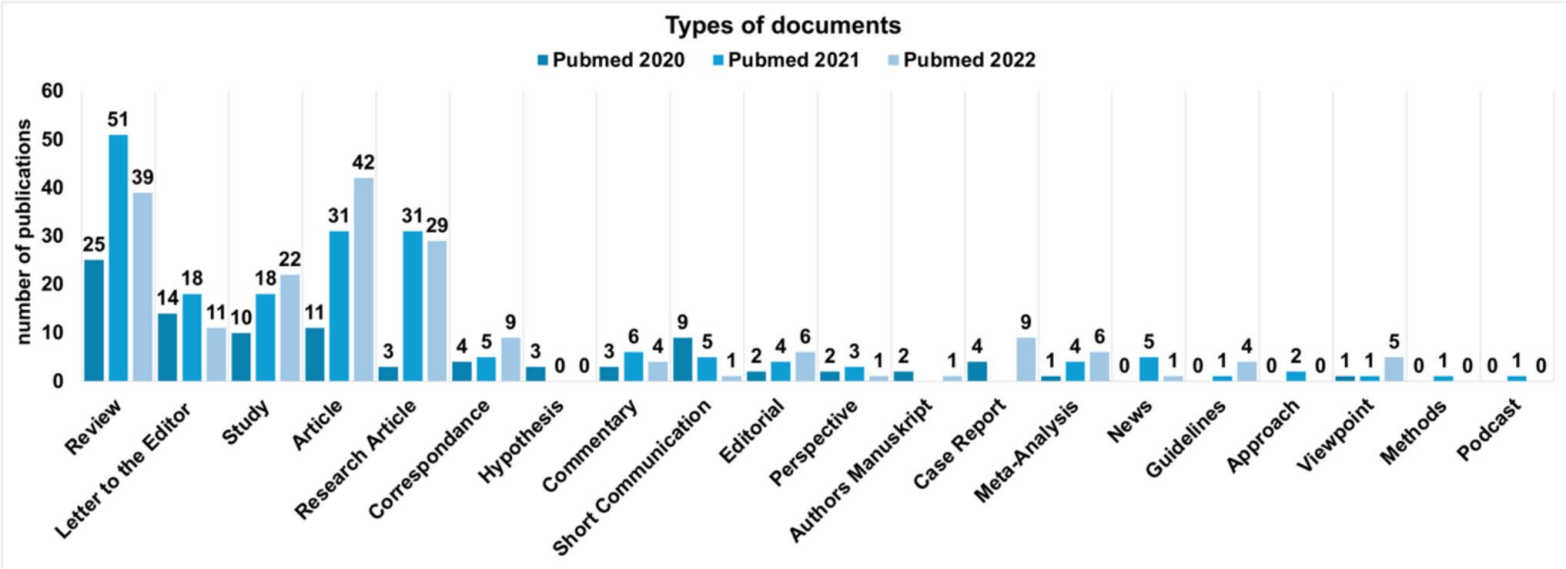
This chart shows the use of the various document types in 2020, 2021 and 2022. The y-axis shows the number of publications

**Fig. 7:**
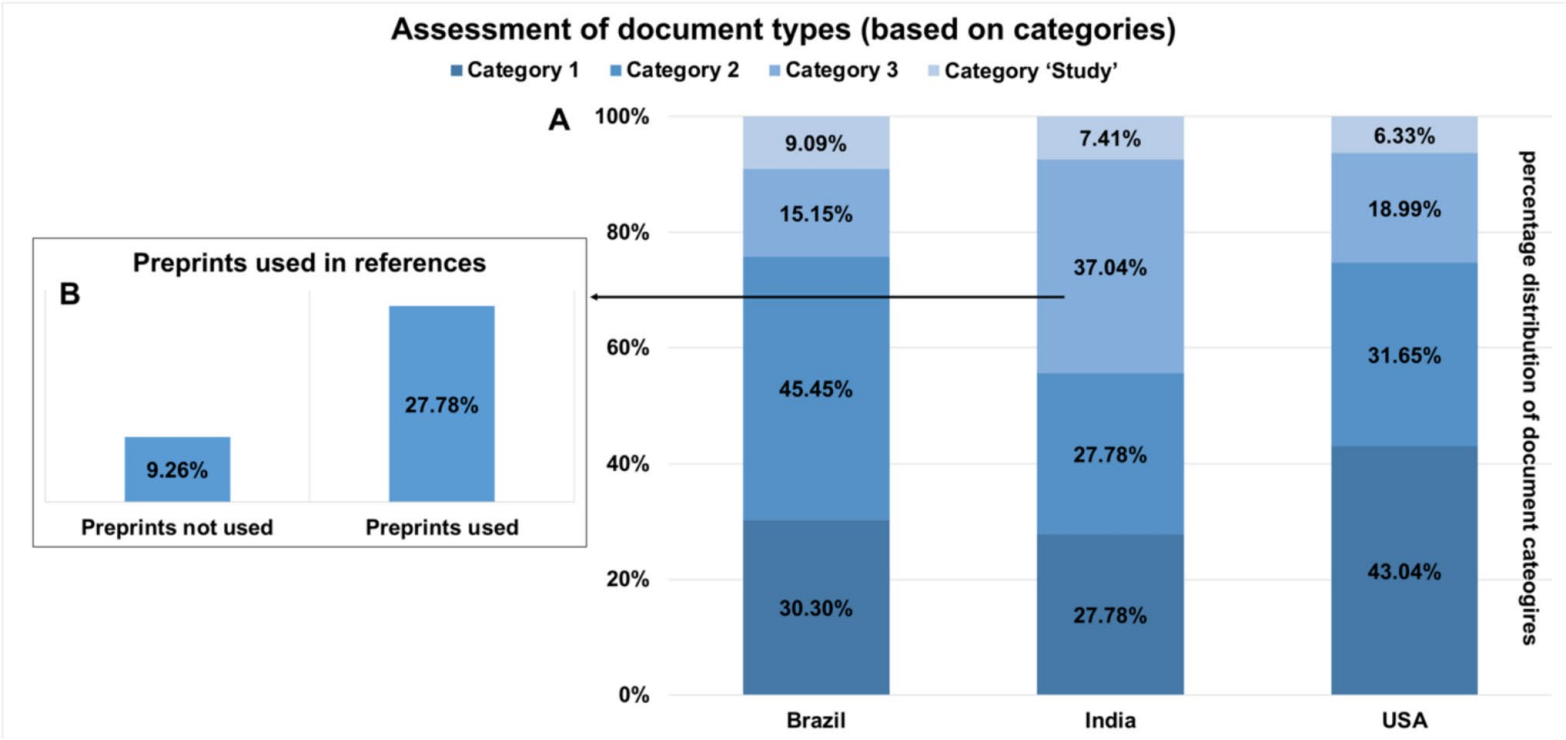
This chart shows the distribution of the different document types in three categories + category ‘study’ for quality assessment. **A** Percentage distribution of document types in the categories within the countries Brazil, India and the USA **B** Percentage distribution of the use of preprints in the references in category 3 in India

**Fig. 8:**
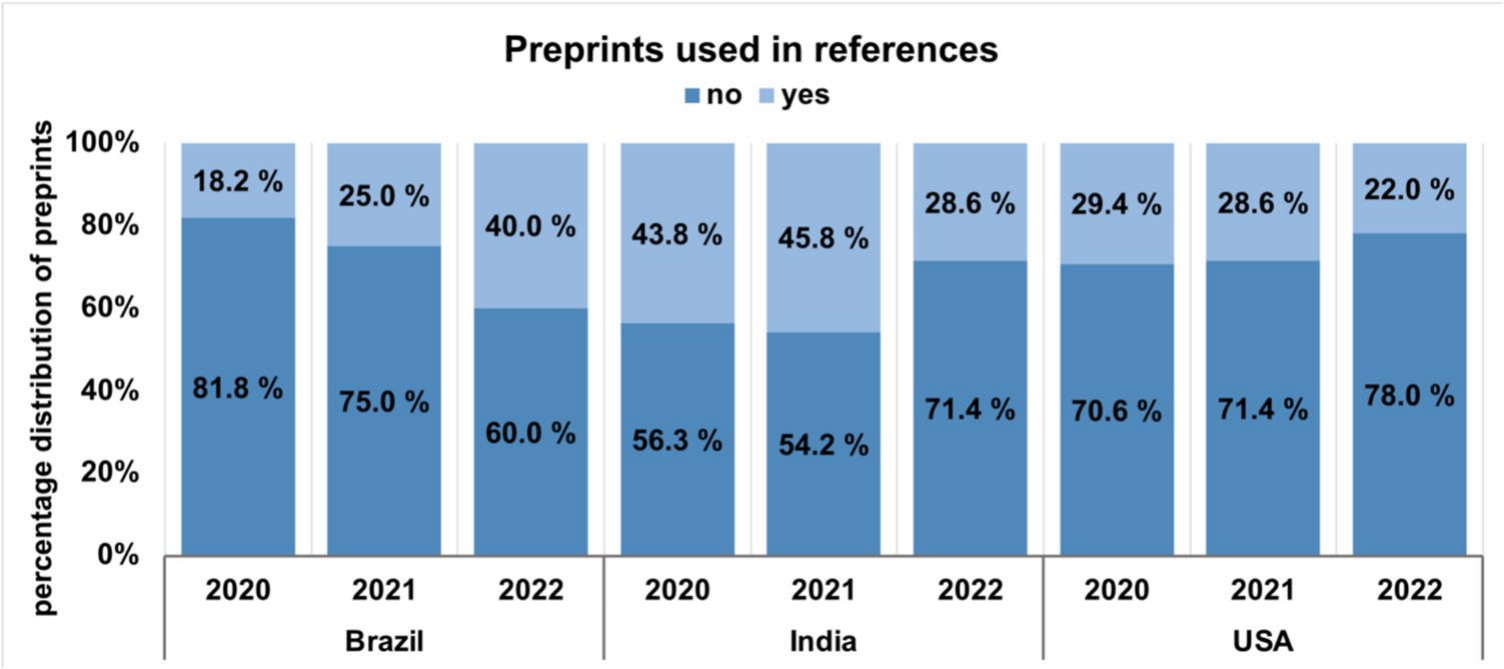
This chart shows the percentage distribution of the usage of preprints in the references in Brazil, India and the USA in 2020, 2021 and 2022

**Fig. 9:**
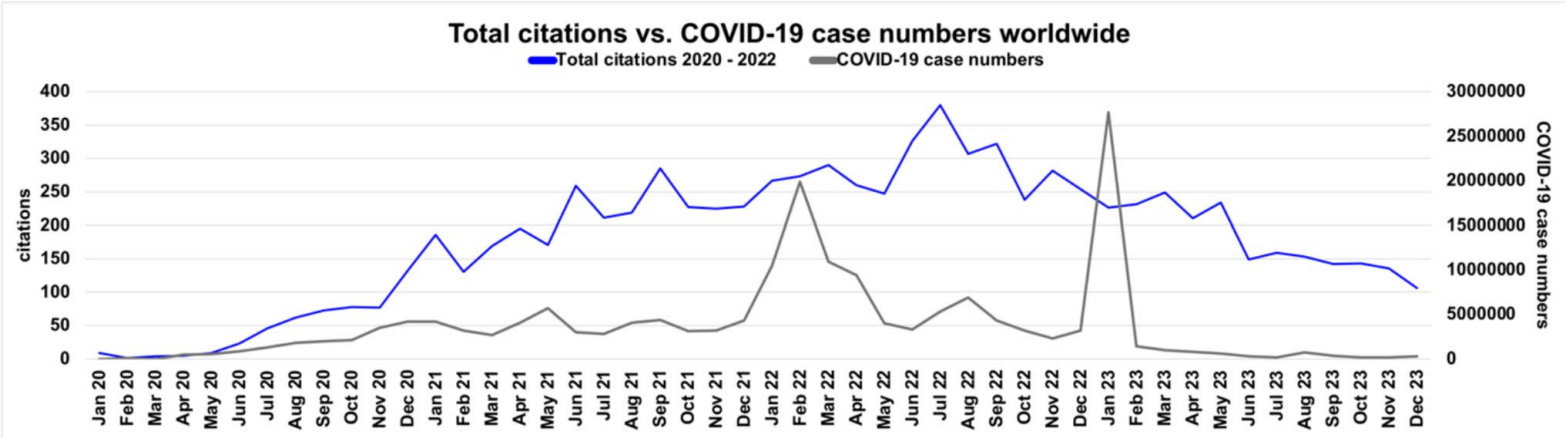
This chart shows the total number of citations in a period from 2020 to 2023 of all included publications from 2020 to 2022 in comparison to the COVID-19 case numbers. The x-axis shows the months, the left y-axis shows the number of citations and the right y-axis shows the number of COVID-19 case numbers

**Fig. 10:**
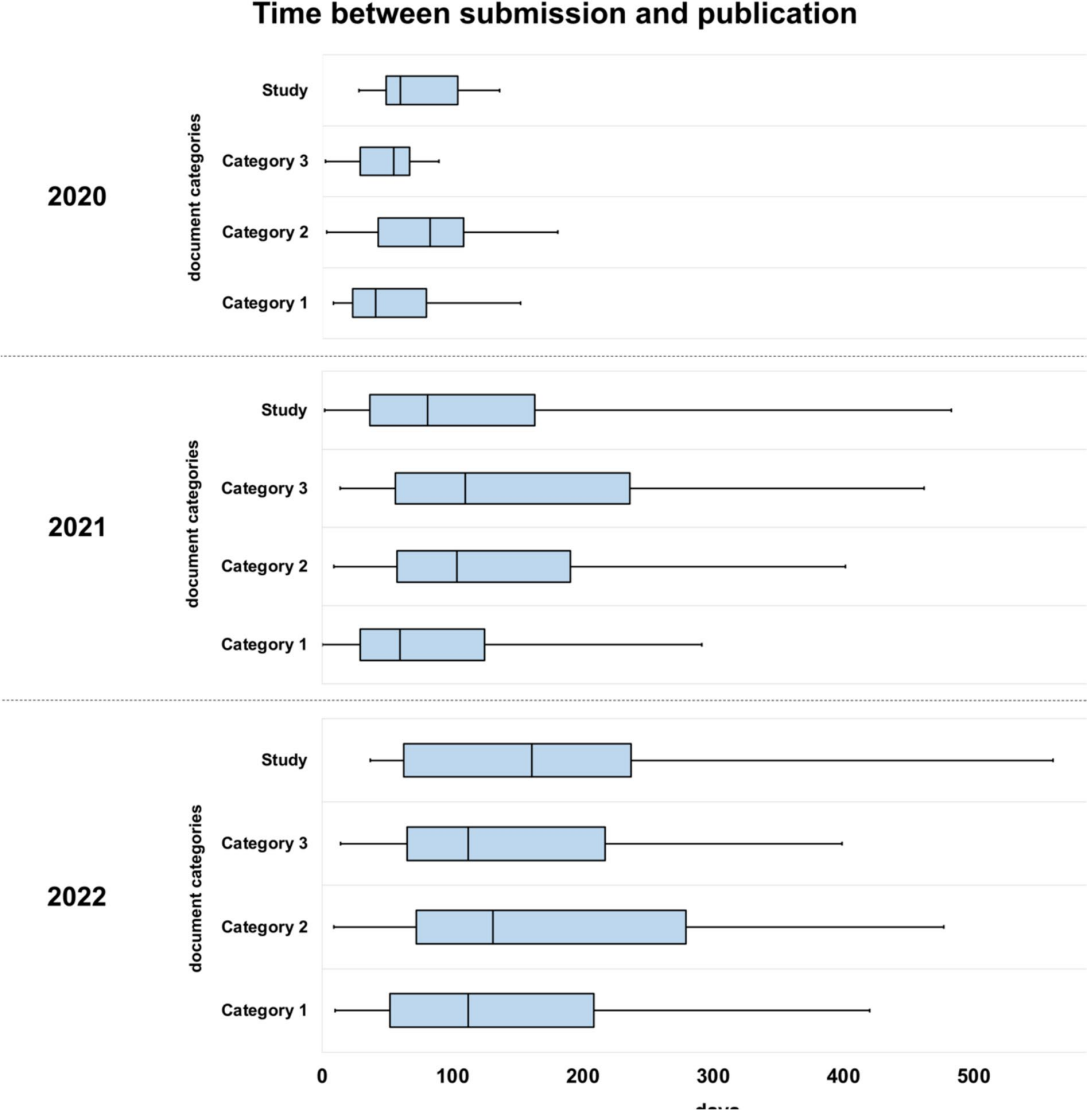
This chart uses boxplots to show the time between submission and acceptance for publication, divided into the three categories according to document type (see Fig. 6, see Table 1) and studies. The x-axis describes the time in days. On the y-axis, the categories are subordinated into the years 2020, 2021 and 2022

**Fig. 11:**
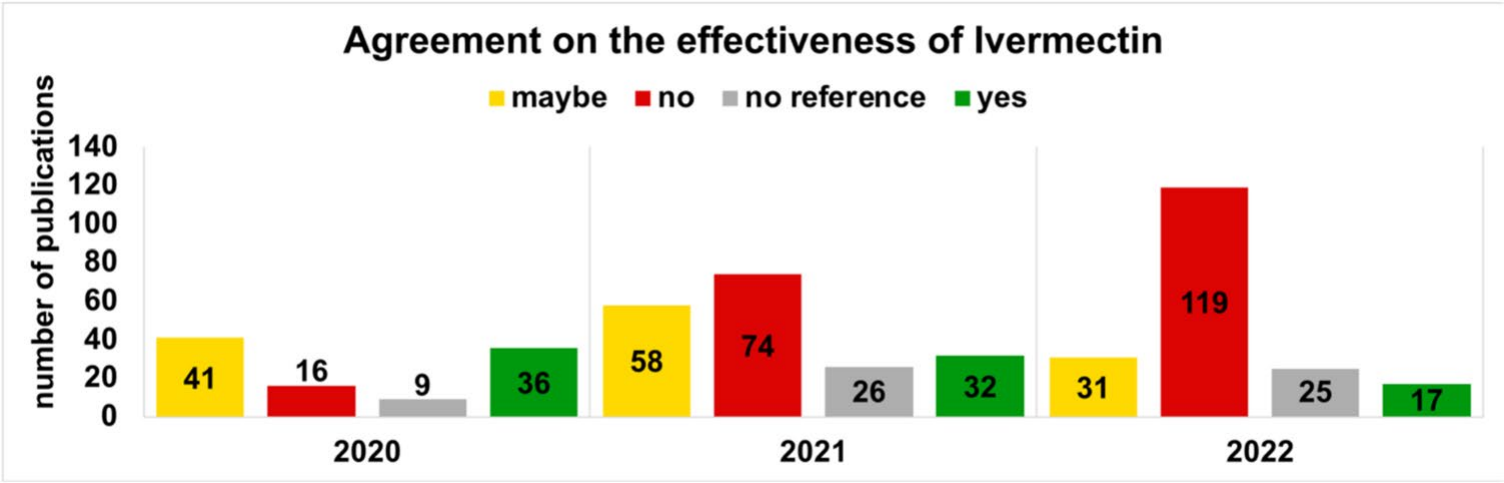
This chart describes the opinion of the individual publications on the efficacy of ivermectin for COVID-19 between 2020 and 2022

**Fig. 12:**
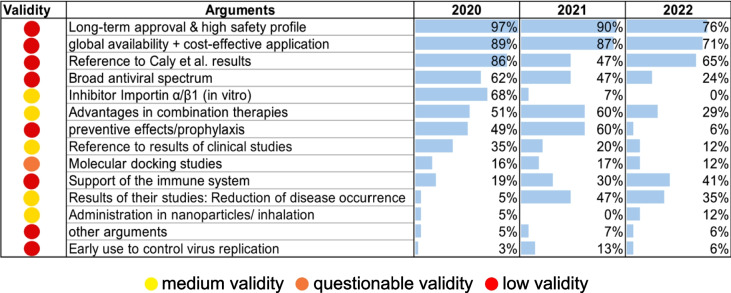
This table lists all the arguments that justify the prescription and use of ivermectin for COVID-19. The percentage use of these arguments in 2020, 2021 and 2022 is shown in the bar chart. The arguments are coded using a traffic light colour system according to their validity

**Fig. 13:**
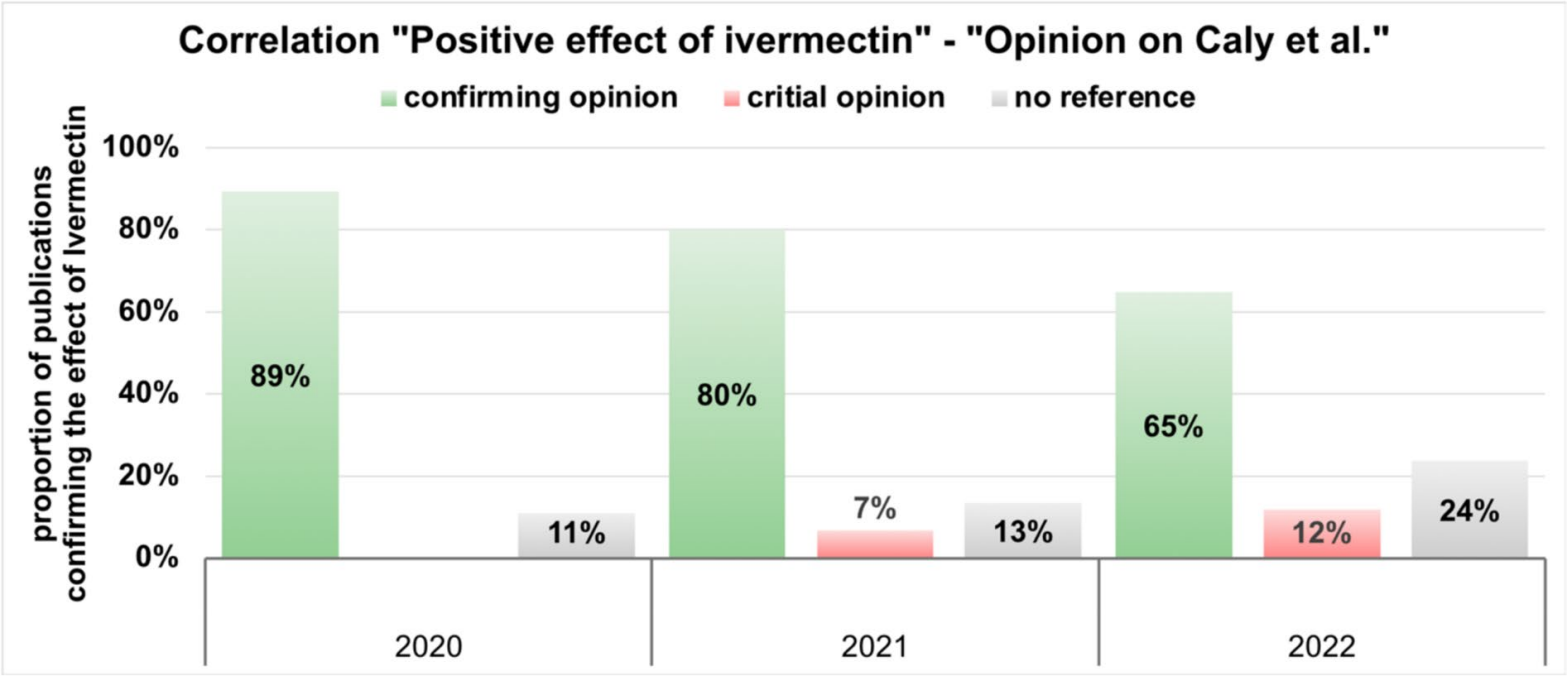
This chart describes the correlation between the statement that ivermectin has a positive effect and the opinion of the individual publications on the study results of the in vitro study by Caly et al. The years 2020, 2021 and 2022 are shown. The proportion of publications confirming the effect of Ivermectin is shown on the y-axis

**Table 2:** This table shows the allocation of the various document types to categories 1, 2 and 3

Category	Document type
1	News, Hypothesis, Commentary, Short Communication, Abstract, Viewpoint, Perspective, Editorial, Letter to the Editor, Correspondance, Case Reports
2	Original Article, Research Article
3	Review + Meta-Analysis, Guidelines, Reviews

**Table 3:** Comparison of the databases PubMed and Web of Science Core Collection

	PubMed	Web of Science Core Collection
Company	National Library of Medicine, neutral, non-commercial, public institute, financed by the National Institute of Health	Clarivate (company bought by Onex Corporation & Baring Private Equity Asia)
Data source	MEDLINE database (< 35 million publications) – the world’s largest biomedical database	Interdisciplinary citation index consisting of several journal indices (21,000 +) as well as collections of conferences and books
Selection process	A committee of 15 members who comprehensively evaluate each journal individually and make a decision about inclusion in the database based on the overall evaluation	100% contract-neutral; strict selection criteria and assignment to various indices

**Table 4:** Summary and comparison of the analysis results of the PubMed and Web of Science databases 2020–2022

Analysis category	PubMed results	Web of Science results
Publications per country	India, USA, Brazil as top contributors, similar distribution across years	Similar trends, top countries consistent with PubMed findings
Number of drugs per category	Increase in diversity of drug mentions, with antivirals dominating initially; shift to anti-inflammatory drugs in 2022	Similar diversity patterns; emphasis on herbal ingredient drugs in later years
Mentions of individual drugs	Ivermectin, HCQ, and remdesivir as top drugs across years; dexamethasone usage increases later	Parallel findings to PubMed; ivermectin consistently most mentioned
Document types usage	Significant use of reviews, clinical studies and articles; rapid publication initially	Similar trends; document types consistent with PubMed but with minor country-specific variations
Citation trends vs. COVID-19 case numbers	Citation trends follow COVID-19 cases with a slight time lag	Citation trends follow COVID-19 cases with a slight time lag; trends consistent with PubMed findings
Publication latency periods	Shortened latency periods initially; increase in later years. Category 3 had the longest delay	Comparable trends, with category 3 showing the longest delays in publication times
Opinion on ivermectin efficacy	Growing skepticism, with rejections increasing sevenfold; affirmations decrease	Declining affirmation of efficacy; trends align with PubMed results
Use of preprints in references	Use of preprints decreased over time in key countries. India cited preprints heavily in category 3	Preprint citation trends match PubMed findings, especially the decline over time
Arguments for ivermectin use	High safety profile and cost-effectiveness frequently cited; arguments often lack validity	Arguments mirror PubMed analysis; cost and safety emphasized despite lacking strong validity
Correlation with Caly et al. study	Strong correlation in 2020; declines in subsequent years as criticism of Caly et al. study rises	Correlation with Caly et al. findings observed in 2020; decreases in 2021–2022, parallel findings to PubMed

**Table 5:** Dose and observed adverse events in the study by Guzzo et al. (2002)

Administered dose (mg)	Approx. dose in mg/kg (assuming ~ 80 kg BW)	Observed adverse events
30 mg	~ 0.375 mg/kg	Occasional dizziness, occasional headaches
60 mg	~ 0.75 mg/kg	Increase in dizziness, rarely mild nausea
90 mg	~ 1.125 mg/kg	More frequent dizziness, mild gastrointestinal complaints, headaches
120 mg	~ 1.5 mg/kg	Increase in dizziness, occasional gastrointestinal complaints, rarely mild skin reactions

**Table 6:** This table shows all the molecular docking studies investigating the receptor binding site of the spike protein

#	Publication	Amino acid residues in the receptor binding site (RBS) of the spike protein	Affinity	Binding energy	Investigation methods
1	Elucidation of the inhibitory activity of ivermectin with host nuclear importin a and several SARS-CoV-2 targets (Bello 2022)	Leu492, Gln493, Gly496, Tyr505	− 9.0 kcal/mol	− 30.60 kcal/mol	MD, MM-PBSA
2	Repurposing approved drugs as inhibitors of SARS-CoV-2 S-protein from molecular modeling and virtual screening (De Oliveira et al. 2021)	Arg403, Ile418, Tyr489, Phe490	− 8.1 kcal/mol	/	MD, MM-PBSA
3	Repurposing of the approved small molecule drugs in order to inhibit SARS-CoV-2 S protein and human ACE2 interaction through virtual screening approaches (Kalhor et al. 2022)	Tyr449, Tyr453, Leu455, Phe456, Ala475, Gly476, Phe486, Asn487, Tyr489, Gln493, Gly496, Gln498, Thr500, Asn501, Gly502, Tyr505	− 10.8697 kcal/mol	− 89.360 kJ/mol	MM-PBSA
4	The binding mechanism of ivermectin and levosalbutamol with spike protein of SARS-CoV-2 (Saha and Raihan 2021)	Leu492, Gln493, Gly496, Tyr505	− 9.0 kcal/mol	− 224 kcal/mol (estimated using DFT)	MD, DFT

**Table 7.**
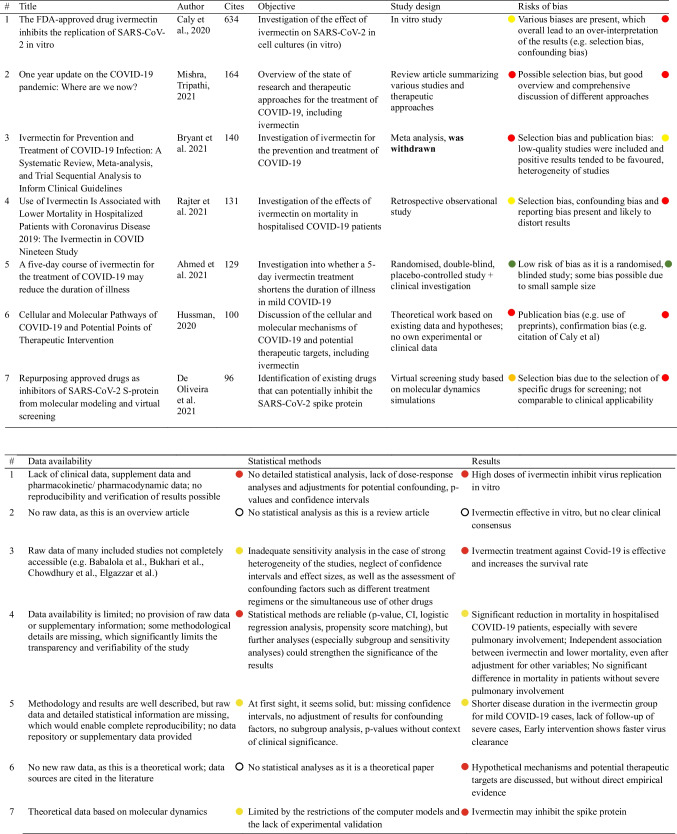

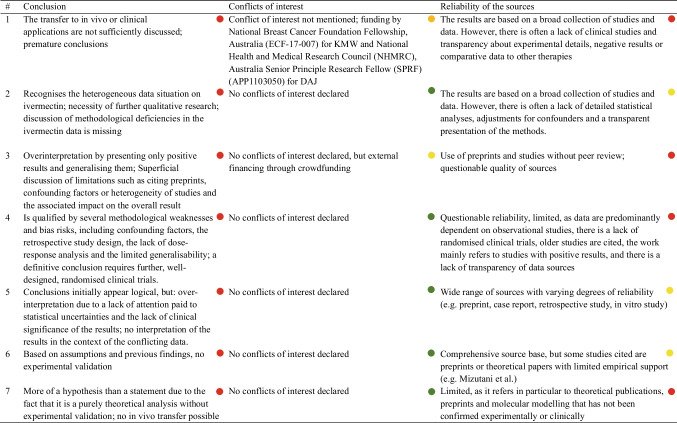
Content analysis of the most cited publications in the PubMed database, key date 30.09.2024

High quality  medium quality  low quality  no assessment

## Results and discussion

### Publications per country

Figure 3 shows the number of publications per country from 2020 to 2022 on a world map. India has authored the most scientific publications between 2020 and 2022. The USA has the second-largest scientific pool of publications with, Brazil follows with 23 publications. European and African countries have significantly fewer publications compared to these three countries. Comparing this with the absolute COVID case numbers (https://data.who.int/dashboards/covid19/cases?n=c; last accessed 10/21/2024), it is clear that the USA, India and Brazil in particular were affected by the COVID-19 pandemic and therefore the focus on finding a drug in these three countries indicates increased research activity. Supplemental Fig. 1 shows the results for the Web of Science research.

### Use of various drugs

This diagram shows the absolute number of drugs in different drug substance groups in the years 2020 to 2022, which were mentioned in all publications (Fig. 4). The drugs were assigned to their drug categories and the total number of mentions was counted. While antiviral drugs were the most frequently mentioned drugs in 2020 and 2021 these changes over time. In 2022, the most frequently counted drugs were those in the category ‘drugs for the treatment of inflammation/autoimmune diseases’. In addition, the use of herbal ingredient drugs is increasing. These fluctuations show how medical research interest dynamically adapts to medical challenges and scientific findings. Overall, the range of different drug groups is widening from 2020 to 2022. It is possible that earlier tests did not show sufficient effect or that more time could be spent on research with new findings and indications for further active ingredients due to the progression over time.

Another analysis looks at the total number of mentions of individual drugs (only drugs with more than 30 mentions included) over the period 2020—2022, depicted in Fig. 5. The graphic shows that the drugs ivermectin, hydroxychloroquine and chloroquine (HCQ + CQ), and remdesivir are amongst the most frequently mentioned drugs every year.

Dexamethasone is a drug in the category ‘drugs for the treatment of inflammation/autoimmune diseases’ and increased from 2021 to 2022. This result corroborates the findings in Fig. 4. Ivermectin remains the most frequently studied and discussed drug in COVID-19 research in this paper. Both Figs. 4 and 5 and the results underline the change in drug research against COVID-19 and the ongoing discussion about the effectiveness of ivermectin. The results for the Web of Science analysis are shown in Supplemental Figs. 2 and 3.

### Document types and preprints

Figure 6 shows the use of the various document types in 2020, 2021 and 2022. Overall, there are a large number of different document types that were used. Five document types were used very frequently: reviews, letter to the editor, study, article and research article. Reviews were used most frequently in the three years. Reviews are mainly based on research papers and original articles, which were only published in larger numbers over time. This ratio is unbalanced. As a result, at the beginning of the pandemic, many reviews were based on the same research, from which a loss of quality can be inferred. A detailed overview of how often each type of document was used per year in the Web of Science database can be found in the appendix (Supplemental Fig. 4).

Figure 7A shows the percentage distribution of the document categories (Table 2) for the countries USA, India and Brazil. When comparing these between the countries, India stands out with the highest percentage of category three. This percentage is twice as high as the percentage for the USA and Brazil. The second step in assessing scientific rigour is to analyse the distribution of publications that cite preprints as a source (Fig. 7B). Since India has a particularly high number of category 3 publications, we scanned the source lists of these publications for preprints. Two thirds of category 3 publications in India cite preprints in their bibliography. The results for the Web of Science analysis are shown in Supplemental Fig. 5.

During global crises, scientists face the challenge of publishing results quickly. Compared to other global outbreaks caused by RNA viruses (e.g. Ebola epidemic, Zika virus epidemic), this has led to significantly more preprints being published during the pandemic (Fraser et al. 2021). Rapid access to current research results is seen as an advantage (Glymour et al. 2023). As many articles are hidden by paywalls, the proportion of open access publications for COVID-19 has increased significantly during the pandemic. It also shows that a larger number of authors publishing on the topic of COVID-19 published a preprint for the first time, especially in India, UK, US and Germany (Fraser et al. 2021).

However, the use of preprints harbours some risks. Firstly, the quality of publications is reduced. The peer review process, an important instance in the publication process, is not carried out, which increases the likelihood of errors or unverified claims (Fraser et al. 2021). Especially with the explosive increase in preprints in this case, there is a risk that important research will be overlooked (Kwon 2020). This leads to a loss of quality and the spread of misinformation. It is very difficult for scientists to keep up with the amount of information. This also creates problems in assessing the credibility of preprints (Brainard 2020). A detailed breakdown of the use of preprints within the categories per country can be found in the appendix (Supplemental Fig. 13).

Figure 8 shows the overall distribution of preprints in the references of all publications. While the proportion of cited preprints in Brazil doubled over time, the proportion in India and the USA fell by around 25%. Studies on the dissemination of preprints in COVID-19 research show that COVID-19-related preprints were accessed and cited more frequently than non-COVID-19-related preprints (Fraser et al.).

In the case of crises or pandemics, preprint publications are usually the only source of data on which further research and publications can be based (Johansson et al. 2018). The chart shows that in India and the USA, the peer review process has been increasingly used for many publications over time. Previous analyses show that around 70% of these preprints are eventually published in peer-reviewed scientific journals. These publications ensure greater evidence and certainty in their statements. In turn, higher quality research can be conducted on this basis (Smith 2006). The results for the Web of Science analysis are shown in Supplemental Figs. 6 and 7.

### Citation rates and COVID-19 case numbers

Figure 9 shows the citation rates of the publications and the monthly WHO case numbers over a period from January 2020 to December 2023. If we take the frequency of citations as a measure of interest in and research on COVID-19, they follow the COVID-19 case numbers with a slight time lag. This shows that, despite the very short latency period of the publications from weeks to months (compare Fig. 9), the scientific debate is ongoing. Despite warnings from major scientific institutions such as the FDA (/https://www.cnn.com/2021/03/05/health/ivermectin-covid-19-fda-statement-wellness/index.html, last accessed 06/16/2024), the EMA (https://www.ema.europa.eu/en/news/ema-advises-against-use-ivermectin-prevention-or-treatment-covid-19-outside-randomised-clinical-trials, last accessed 06/1 6/2024) or the WHO (https://www.who.int/news-room/feature-stories/detail/who-advises-that-ivermectin-only-be-used-to-treat-covid-19-within-clinical-trials, last accessed 06/16/2024), the citation figures adapt to the case numbers. This indirectly shows how much trust exists in the political propaganda of right-wing parties. As soon as a new peak in the number of COVID-19 cases was reached and the emergency situation continued to worsen, parties promoted alternative treatment concepts to vaccination (Serrano-Alarcón et al. 2023). Supplemental Fig. 8 shows the results for the Web of Science research.

### Latency periods of the publication process

Figure 10 shows the latency periods between submission and publication broken down by document category over the three years. The average time between submission and publication increased for all categories from 2020 to 2022. For example, the median processing time in category 1 increased from 50 to 100 days. Particularly for publications from category 3, the processing time increased continuously, which could indicate increased quality control. It is striking that publications in category 1 show a shorter time to publication overall than publications in category 3. A change over the years is noticeable as well. During the initial phase of the pandemic in 2020, all publications were published very rapidly to provide time-critical information promptly. In 2021 and 2022, the processing time for publications, especially category 3, increased. To summarise, the chart shows that the publication process was initially accelerated during the pandemic. Horbach’s publication supports these findings. He examined the publication process of 669 publications from 14 different medical journals during the COVID-19 pandemic. He observed that the time between submission and publication was significantly reduced, on average by 49%. It is also noted that the shortening of publication processes only applies to COVID-19-related publications (Horbach 2020).

Speeding up the review and publication process made it possible to provide information faster, which was crucial for the immediate response to the crisis (Horbach 2020). Even if the average time span increases, the results must be critically contextualised. In the usual scientific publication process, the review process can last several months to years (Björk and Solomon 2013). The acceleration thus means an increased risk of methodological weaknesses and scientific manipulation due to insufficient peer review in the short term, and the weakening of scientific databases in the long-term (Dimitrios 2020).

The ANOVA analysis showed that the duration between submission and publication (measured in days) differs significantly between the years 2020–2022 for categories one to three: in category 1: *F* (2, 84) = 6.361, *p* = 0.003; in category 2: *F* (2, 135) = 6.389, *p* = 0.002; in category 3: *F* (2, 102) = 6.473, *p* = 0.002. There is no statistically significant difference between the publication time of the years 2020—2022 in the category ‘study’: *F* (2, 47) = 1.799, *p* = 0.177.

The Bonferroni-corrected post-hoc analysis revealed several significant differences (p < 0.05) in the number of days to publication; in category 1 between 2020 and 2022 (*M*_Diff_ = 54.21, 95% CI [17.05, 91.37]), in category 2 between 2020 and 2022 (*M*_Diff_ = 58.37, 95%-CI [11.45, 105.29]) and between 2021 and 2022 (*M*_Diff_ = 37.06, 95%-CI [5.05, 69.07]) and in category 3 between 2020 and 2021 (*M*_Diff_ = 77.38, 95%-CI [24.75, 130.00]) and between 2020 and 2022 (*M*_Diff_ = 63.56, 95%-CI [9.83, 117.29]). The complete results can be found in the Supplemental Tables 2, 3 and 4. Overall, the results underline the dynamics and influence of the COVID-19 pandemic on scientific publishing. The data of the Web of Science analysis is presented in Supplemental Fig. 12 and Supplemental Tables 5, 6 and 7.

### Opinion on the effectiveness of ivermectin

All publications were examined to see whether they agreed or disagreed with the assumed effectiveness of ivermectin in COVID-19. The rejection of the effectiveness and use of ivermectin increased sevenfold, from 16 publications with a negative attitude to 119 publications. In contrast, the number of publications with an affirmative opinion decreased (Fig. 11).

Fourteen main arguments are used to justify the prescription and effectiveness of ivermectin. Most publications argue that ivermectin can be prescribed without concern due to its long-term approval and high safety profile (Babalola et al. 2022; Bryant et al. 2021; Caly et al. 2020; De Oliveira et al. 2021; Mishra and Tripathi 2021). In addition, its global availability, coupled with a low-cost prescription, is an argument in its favour. All further arguments and their different percentage usage can be found in Fig. 12. For the Web of Science data analysis, the results are depicted in Supplemental Figs. 9 and 10. In a further step, we examined the arguments for their validity and categorised them using a traffic light colour system.

We have analysed all arguments in detail and assessed their validity. The following arguments are classified in the red area due to a lack of validity:

- Long-standing approval and high safety profile in the original indication
- Global availability and cost-effective use
- Broad antiviral spectrum in in vitro studies on the inhibition of RNA/DNA helicase
- Prophylactic use/lower case numbers in countries that take ivermectin prophylactically
- Support of the immune system by preventing a cytokine storm
- Early use to control virus replication
- Others:
  o Because it is effective against SARS-CoV-1, it is also effective against SARS-CoV-2

All these arguments are taken out of context and incorrectly applied to the antiviral effect of ivermectin. Therefore, these arguments do not justify the prescription and use of ivermectin in the COVID-19 pandemic and are unusable.

Many publications that consider ivermectin to be effective refer to the results of the in vitro study ‘The FDA-approved drug ivermectin inhibits the replication of SARS-CoV-2 in vitro’ by Caly et al. (2020). In this study, an in vitro experiment was conducted with Vero/hSLAM cells infected with Covid-19. A single concentration of 5 µM ivermectin was added to these and examined by RT-PCR for 3 days. The result after 48 h shows an approx. 5000-fold virus reduction compared to the control group.

Based on the assumed distribution volume of 3.3 L/kg (González Canga et al. 2008), a dose of 14.44 mg/kg of ivermectin would be needed to achieve a plasma concentration of 5 µM. However, the FDA only approved a dosage of 0.2 mg/kg body weight for humans for the treatment of strongyloidiasis (https://www.merck.com/product/usa/pi_circulars/s/stromectol/stromectol_pi.pdf, last accessed 11/18/2024).

In therapeutic dosage as part of anthelmintic therapy, adverse drug reactions are described in the information for healthcare professionals for Ivermectin tablets (Ratiopharm) for various indications. For example, general adverse effects are: transient hypereosinophilia, liver dysfunction, neurotoxicity, fatigue, headache and much more (https://www.ratiopharm.de/assets/products/de/label/Ivermectin-ratiopharm%203%20mg%20Tabletten%20-%205.pdf?pzn=18030208, last accessed 12/16/2024).

At excessively high dosages and with accumulation in the body, the following adverse effects result, which were published in the product information sheet: dizziness, nausea, diarrhoea and, rarely, neurological symptoms such as cramps. As the dosage increases, so does the severity of the side effects (https://www.ratiopharm.de/assets/products/de/label/Ivermectin-ratiopharm%203%20mg%20Tabletten%20-%205.pdf?pzn=18030208, last accessed 12/16/2024). The results are illustrated in Table 5. An animal study shows that the lethal dose (LD_50_) is 50 mg/kg. This is also confirmed by the data sheet for Stromectol (https://www.accessdata.fda.gov/drugsatfda_docs/nda/98/50–742 s001_Stromectol.cfm, last accessed 11/18/2024). Thus, with a dose of > 14 mg/kg of ivermectin, substantial toxicity is expected.

The study by Caly et al. was submitted on 18/03/2020 and published on 03/04/2020. This corresponds to a latency period of 16 days. Compared to Fig. 10, this is a very short latency period. Thus, it is doubtful to what extent a high-quality peer review process has taken place to examine the experiment and the argumentation regarding the transferability of the results to in vivo experiments and application. In this context, we analysed the connection between the endorsement of the effectiveness of ivermectin and the opinion of the individual publications on the results of the in vitro study by Caly et al. Only publications that agree with the effect and usage of ivermectin were included (Fig. 13, Web of Science: Supplemental Fig. 11). In 2020, 89% of these publications also agree with the results of the study by Caly et al. In 2021, the percentage was 80%, and in 2022, it was 65%. This shows how large the share of scientific publications is that agree with the results of Caly et al. A tabular list of all publications with their opinion on the publication by Caly et al. can be found in the supplement (Supplemental Table 8).

Another argument that we examined is the reliance on results from molecular docking studies. The docking studies use the docking programmes Auto Dock 4.2 or Auto Dock Vina. However, the reported targets are highly inconsistent and methodologically divergent. Often, no more precise analysis of the exact binding sites takes place, but only a mention of these targets. To present this in more detail, we have focused on four publications that examine the receptor binding domain (RBD) of the SARS-CoV-2 spike protein (Table 6). For the Web of Science analysis, we focused on six publications (Supplemental Table 1).

Within this investigation, there is an inhomogeneity in the identified amino acid residues as well as the binding affinity, binding energy and investigation methods. In total, all molecular docking studies are not supported by further experimental research. There is no consistent use of different methods for a detailed examination of the protein dynamics and the quality of the 3D structure of the target protein, and values such as binding energy are missing.

To analyse publication behaviour, we conducted a content analysis of the most cited publications. Between 2020 and 2022, seven publications with over 96 citations were found in the PubMed database (Ahmed et al. 2021; Bryant et al. 2021; Caly et al. 2020; De Oliveira et al. 2021; Hussman 2020; Mishra and Tripathi 2021; Rajter et al. 2021). The analysis examined the methodological quality and reliability of the studies that were frequently used as a basis for assessing the effectiveness of ivermectin against COVID-19. This allowed conclusions to be drawn about publication behaviour during the pandemic.

Table 7 highlights the methodological weaknesses and risks of bias in the highly cited papers, such as inadequate statistical analyses, missing data availability and a lack of transparency. It shows that positive results are often over-interpreted and methodological biases are insufficiently addressed. The traffic light system clearly summarises the quality assessment.

The study by Caly et al. was by far the most cited, with 634 citations. In addition to the already discussed lack of transferability of the results to clinical scenarios, there are further methodological weaknesses. The data and statistical methodology are not available for a repeat of the experiment and possible distortions are not addressed.

The studies show various risks of bias, which affect the validity of the study results and often lead to an overestimation of positive effects. All studies show limited accessibility of raw data and limited transparency of methodology, which significantly limits critical review, reproducibility and independent verification of results.

The conclusions of the analysed papers are unbalanced and tend to emphasise positive effects without sufficiently discussing the methodological limitations. Many of the sources cited in the analysed publications are based on preprints and other non-peer-reviewed papers.

The analysis shows that methodological weaknesses not only reduce the quality of individual studies based on them but also undermine the scientific validity of the evidence base as a whole. A scientifically based evaluation of the actual efficacy of ivermectin is made more difficult (Bramstedt 2020), (http://www.sciencedaily.com/releases/2020/10/201001200246.htm, last accessed 11/17/2024).

The pandemic has thus provided an example of how vulnerable scientific procedures are under extreme conditions and how easily they can be distorted in such situations.

One particularly problematic aspect here is the absurdity with which ivermectin is being discussed: Although methodological weaknesses are clearly evident in many studies, the drug is given a veritable ‘salvation status’ within certain social groups and political camps. This often completely ignores the fact that much of the data is either based on inadequate studies or merely reflects theoretical models and assumptions. Proven facts, preliminary study results and conspiracy theories exist side by side and are equally used to support certain narratives. Ivermectin seems to have become a controversial political issue in which the actual question of scientific evidence takes a back seat. This dynamic illustrates the extent to which scientific work can become unbalanced once a debate has become politicised. This development is not only scientifically questionable, but can also have far-reaching socio-political consequences. If it later becomes clear that political decisions or clinical guidelines have been based on dubious evidence, a far-reaching loss of trust in science and politics can be predicted. At the same time, serious research endeavours are likely to come under greater pressure if flawed studies and publications are quickly accepted in the broad discourse, creating an apparent evidence base.

## Limitations

This analysis covered the period of the COVID-19 pandemic from 2020 to 2022. This time frame allows for a focused examination of the most relevant publications during the global COVID-19 pandemic. Publications released after this period were not considered. Therefore, the time constraint and its potential impact on the completeness of the analysis should be considered when interpreting the results.

The decision to include only English-language publications in the analysis could lead to the omission of important scientific contributions written in other languages. This language restriction may influence the global perspective and relevance of the results, as potentially valuable insights from non-English-language studies are not considered.

The classification of categories for the evaluation of evidence is based on the document designations, without a separate assessment of publication bias. This methodological decision could have an impact on the interpretation, since the influence of publication bias on the evidence evaluation is not explicitly considered. In addition, the document designations are not used in the same way in every publisher and journal. This makes it difficult to assign them correctly.

## Conclusion

The content analysis of the most frequently cited ivermectin publications forms the thematic framework for this paper. The analysis was conducted to obtain an objective assessment of the scientific quality of the underlying studies that served as the basis for many subsequent papers on ivermectin as a potential COVID-19 treatment. The results of this work emphasise how methodological weaknesses, data gaps and biases in basic studies can impact the entire research chain and lead to distorted scientific and clinical perceptions.

A key finding is that many of the frequently cited ivermectin studies do not fulfil the scientific quality standards required for robust evidence. Methodological errors such as the uncritical interpretation of in vitro results, lack of data availability and a high proportion of non-peer-reviewed preprints significantly affect the reliability of the evidence. Such publications contribute to the dissemination of hypotheses about the efficacy of ivermectin without a sufficient empirical basis.

At the same time, an ‘absurd’ and overtly politicised discourse has developed that overshadows established scientific standards with unverified claims and has far-reaching consequences. The results of this study should therefore suggest that in times of crisis, particular attention must be paid to the quality of published research findings.

## Further perspectives

The analysis of scientific publications during the COVID-19 pandemic highlights the urgent need for a continuous critical approach to scientific sources. Despite existing quality analyses of databases, it remains essential to question the methodology and validity of published research results, especially in times of crisis when the pressure to publish is increasing.

At the same time, social dynamics, characterised by the rise of fake news and the influence of right-wing populism, show that a critical approach to all media formats – including scientific publications – is of crucial importance. In the future, effective media criticism and the ability to distinguish well-founded scientific information from misinformation will be essential to strengthen trust in scientific knowledge and counteract its misuse. These contents and critical thinking should be taught and practised at an early age in an age-appropriate way in order to build a critically thinking, self-confident society.

If publications continue to be based on this poor evidence, medical databases will increasingly be ‘cluttered’ with such papers. This becomes particularly relevant if such poor-quality papers are fed into databases used for large language models (artifical intelligence). Since scientists more and more use large language models for ‘writing’ papers, contaminated databases may propagate the dissemination of incorrect information because no critical analysis of the included information took place in the first place. In the long-term, the resulting distortions and misinformation could not only endanger medical care, but also significantly impair the ability of science to progress. It is therefore crucial to support initiatives that enable a rigorous quality analysis of scientific publications and help to expose bias and fake papers.

## Supplementary Information

Below is the link to the electronic supplementary material.

Supplementary file1 (DOCX 1.81 MB)

## Data Availability

All source data for this study are available upon reasonable request from the authors.

## Abbreviations

AfD: Alternative für Deutschland (right-wing party in the Bundestag, Germany)
Ala: Alanin
Arg: Arginin
Asn: Asparagin
Cmax: Maximum plasma concentration
COVID-19: Coronavirus disease 2019
DFT: Density functional theory
EMA: European medicines agency
FDA: Food and drug administration
FPÖ: Freedom party of Austria
GABA: Gamma-aminobutyric acid
Gln: Glutamin
Gly: Glycin
HCQ + CQ: Hydroxychloroquine and chloroquine
Ile: Isoleucin
IMP: Importin
Leu: Leucin
LD_50_: Median lethal contentration
LPV/r: Lopinavir/ritonavir
MD: Molecular dynamics
MM-PBSA: Molecular mechanics poisson–boltzmann surface area
Phe: Phenylalanine
RBS: Receptor binding site
SARS-CoV-2: Severe acute respiratory syndrome coronavirus 2
Thr: Threonine
Tyr: Tyrosine
WHO: World health organisation
