# Supplementary material for: Ivermectin repurposing for COVID-19: pharmacological and bibliometric analysis
Source: Naunyn Schmiedebergs Arch Pharmacol. 2025 May 6;398(11):15475–93. doi: 10.1007/s00210-025-04233-5 (PMC12552315; doi:10.1007/s00210-025-04233-5)
Supplement: Supplementary file 1 — Supplementary file1 (DOCX 1.81 MB) [file 210_2025_4233_MOESM1_ESM.docx]

**Ivermectin repurposing for COVID-19: Pharmacological and bibliometric analysis**

**Maresa Dulle and Roland Seifert**

**Supplemental Figures (Web of Science analysis)**


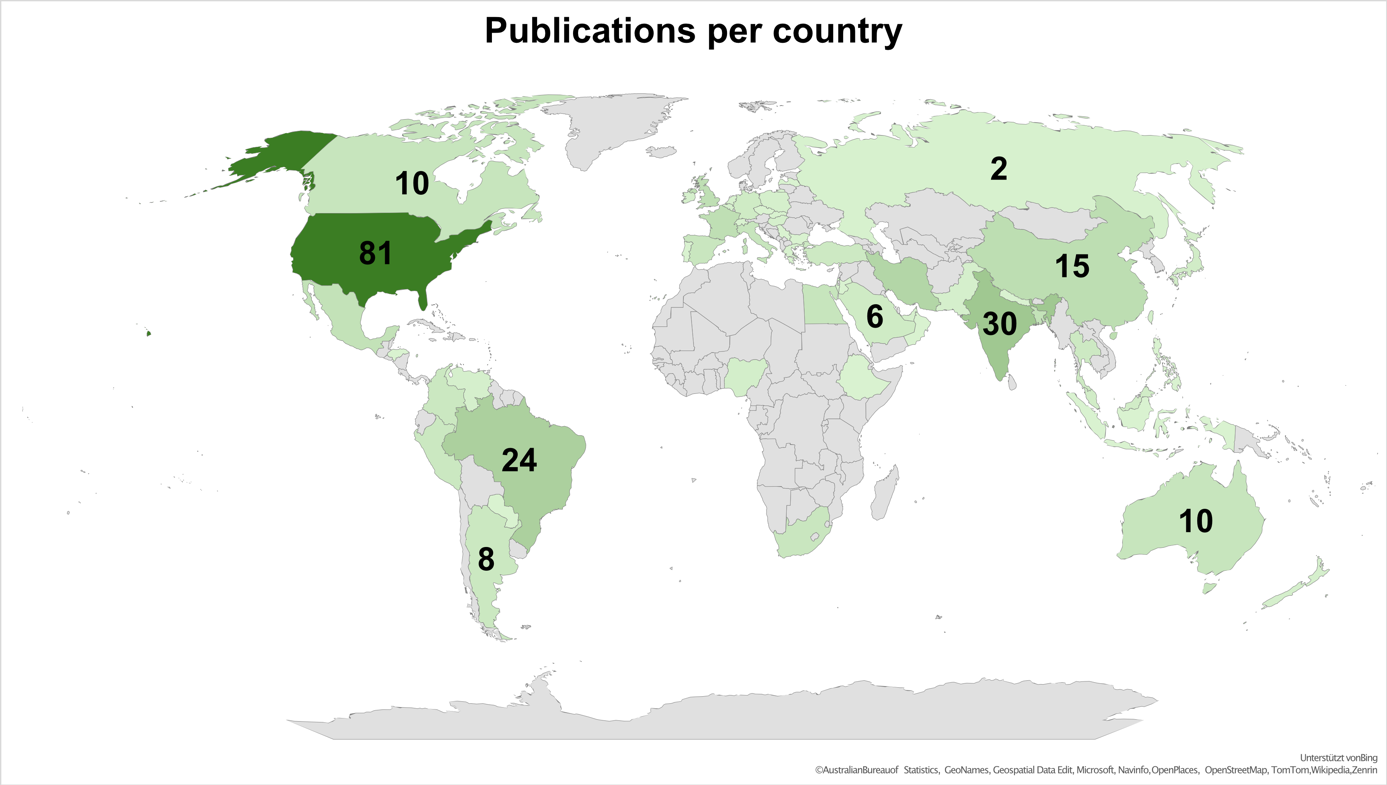


Supplemental Figure 1 The world map shows the number of publications per country in 2020, 2021 and 2022.


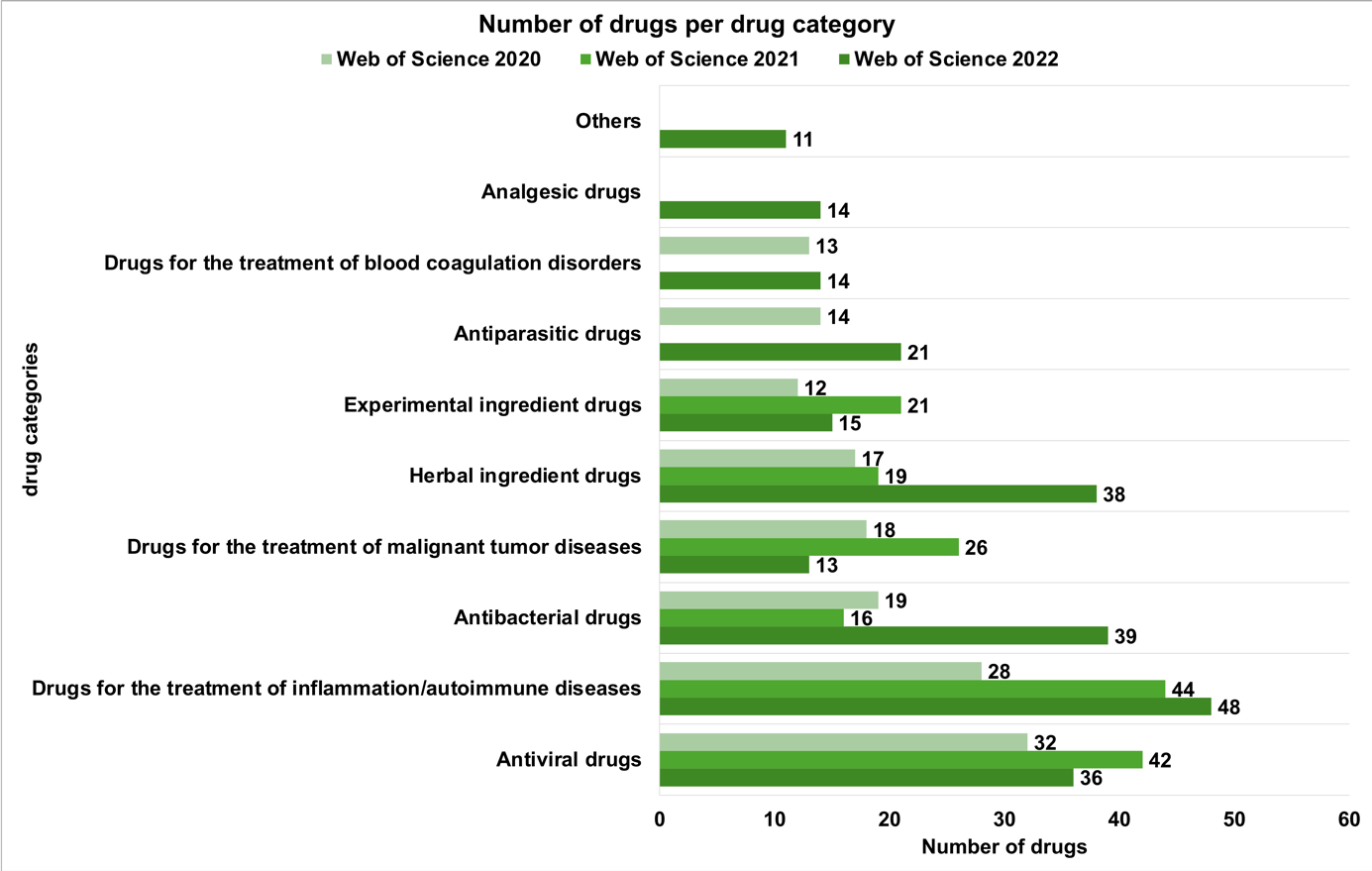


Supplemental Figure 2 The illustration shows the absolute number of drugs per drug category in the years 2020, 2021 and 2022 mentioned in all publications.


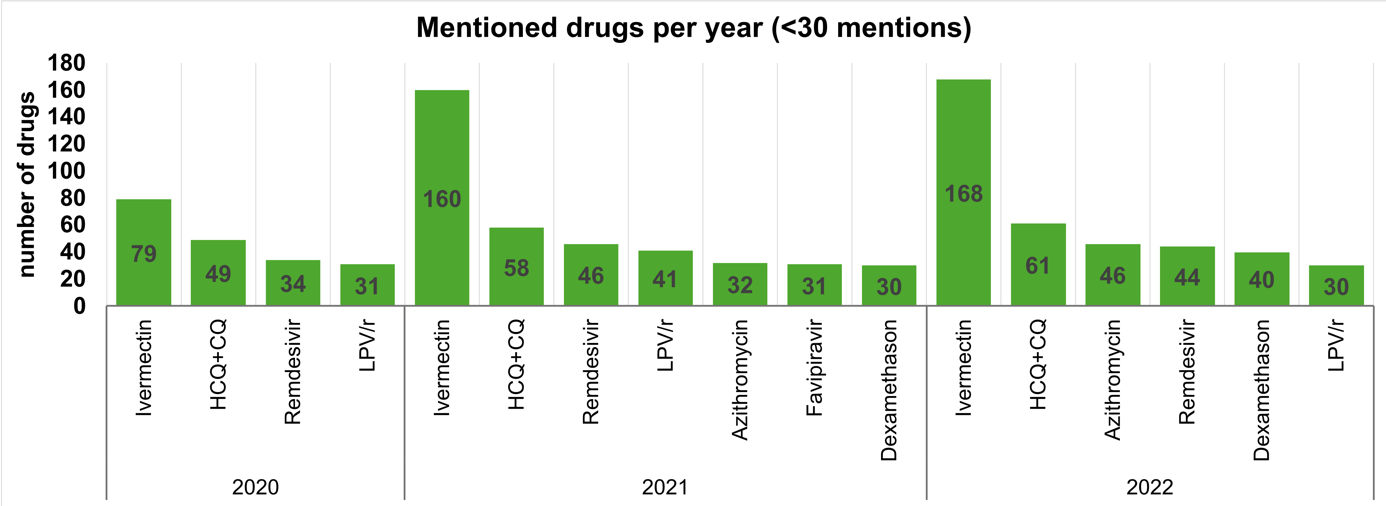


Supplemental Figure 3 Looking at each drug individually, this graph shows the total number of mentions of each drug in the years 2020, 2021 and 2022. Only drugs mentioned more than 30 times are included. The y-axis shows the absolute number of drugs.


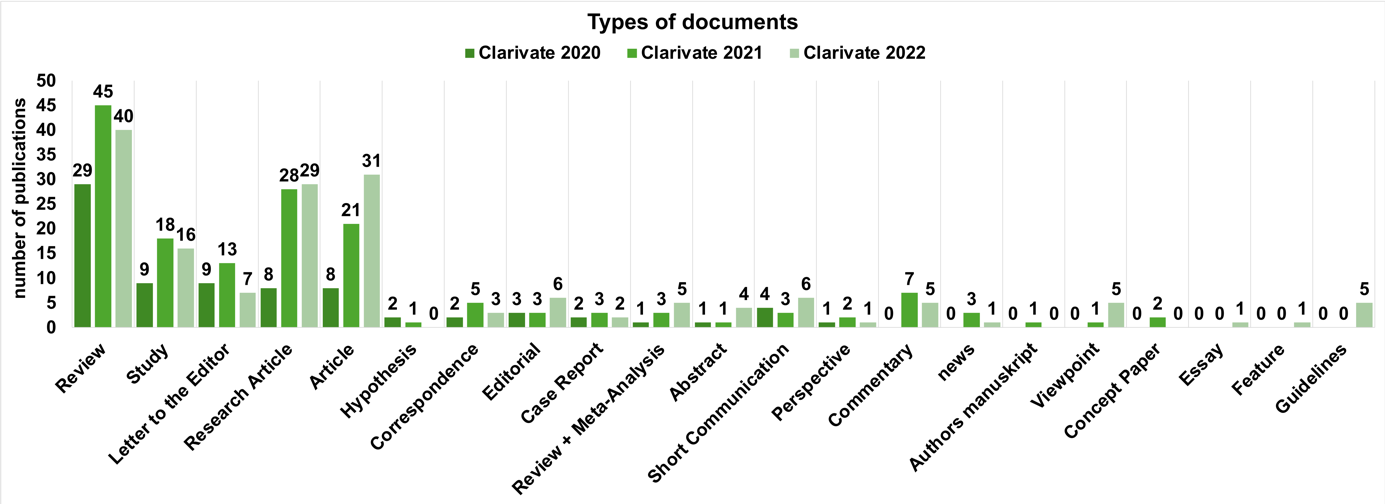


Supplemental Figure 4 Looking at each drug individually, this graph shows the total number of mentions of each drug in the years 2020, 2021 and 2022. Only drugs mentioned more than 30 times are included. The y-axis shows the absolute number of drugs.


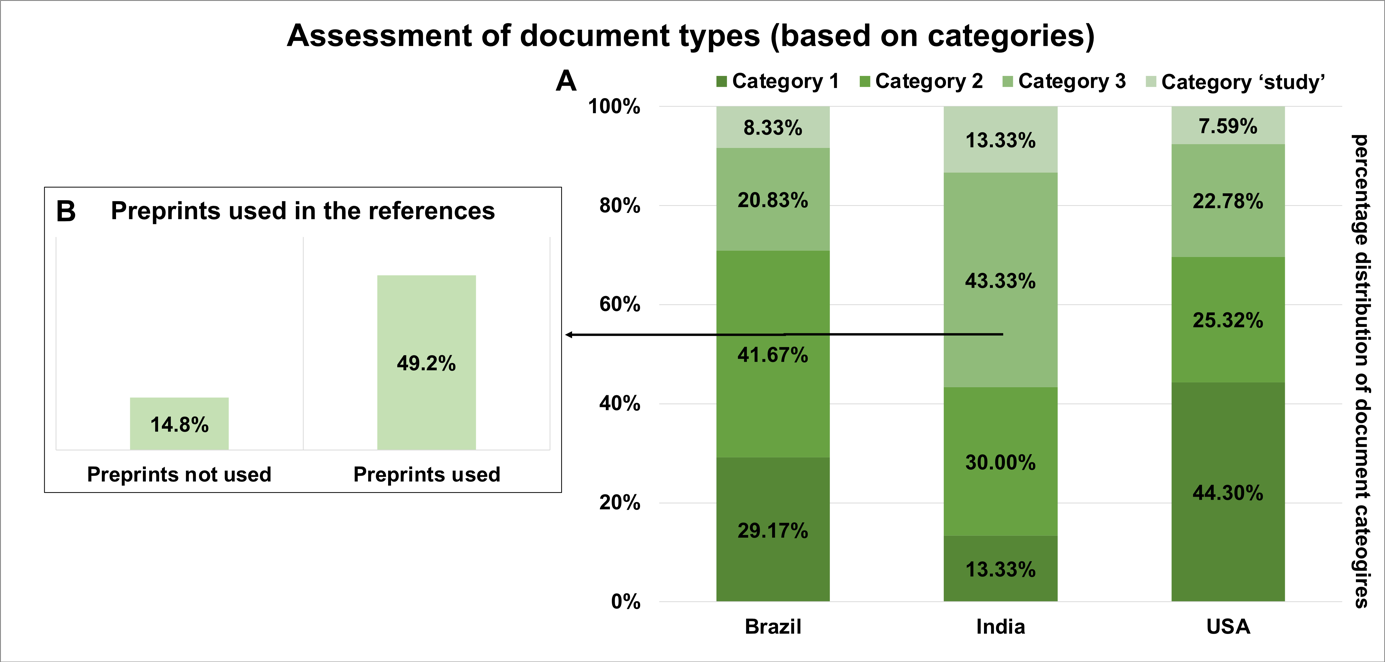


Supplemental Figure 5 This chart shows the distribution of the different document types in the document categories for quality assessment. **A** Percentage distribution of document types in the categories within the countries Brazil, India and the USA **B** Percentage distribution of the use of preprints in the references in category 3 in India


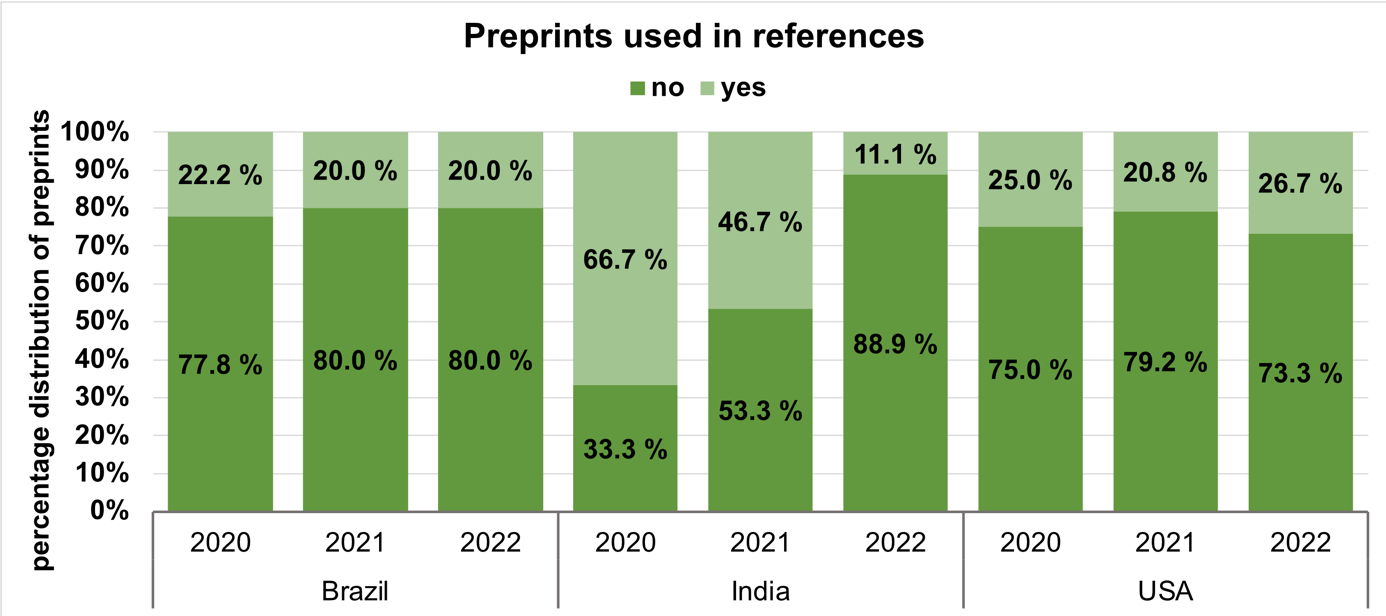


Supplemental Figure 6 This chart shows the percentage distribution of the usage of preprints in the references in Brazil, India and the USA in 2020, 2021 and 2022.


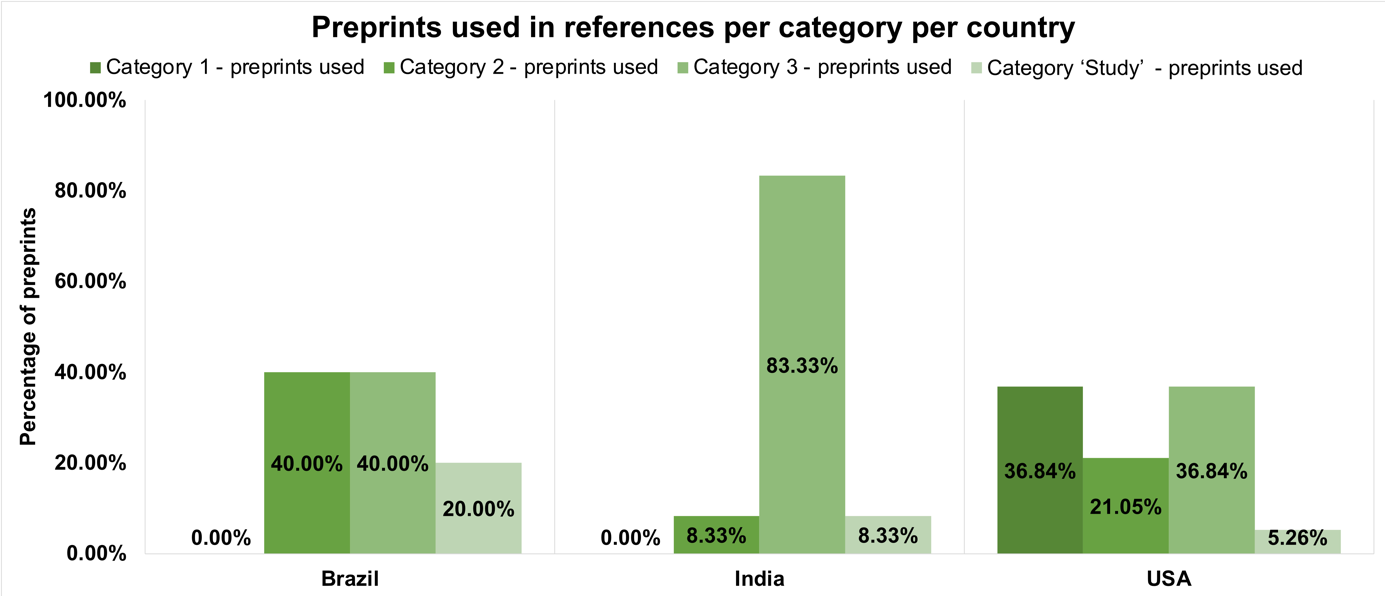


Supplemental Figure 7 This chart shows a breakdown of the percentage distribution of the usage of preprints in the references per document category in Brazil, India and the USA.


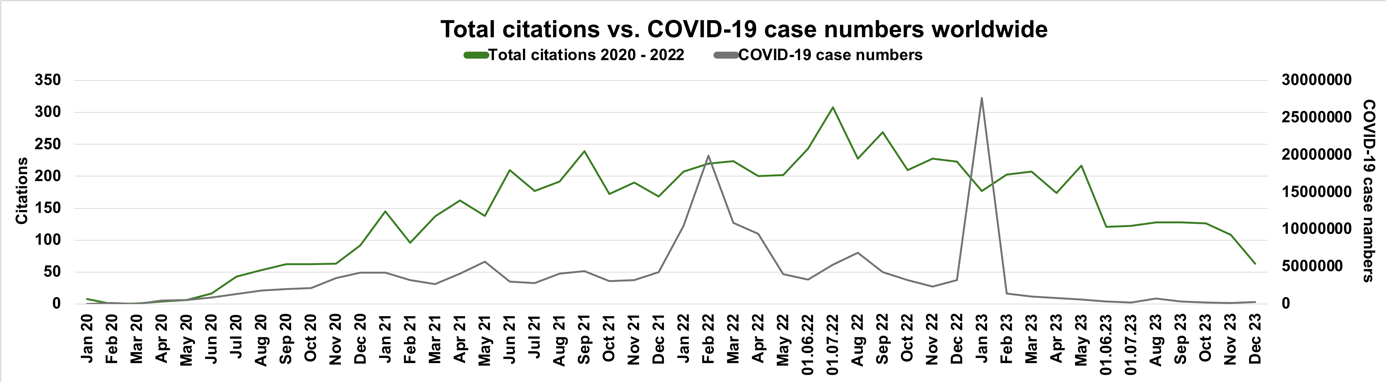


Supplemental Figure 8 This chart shows the total number of citations in a period from 2020 to 2023 of all included publications from 2020 to 2022 in comparison to the COVID-19 case numbers. The x-axis shows the months, the left y-axis shows the number of citations and the right y-axis shows the number of COVID-19 case numbers.


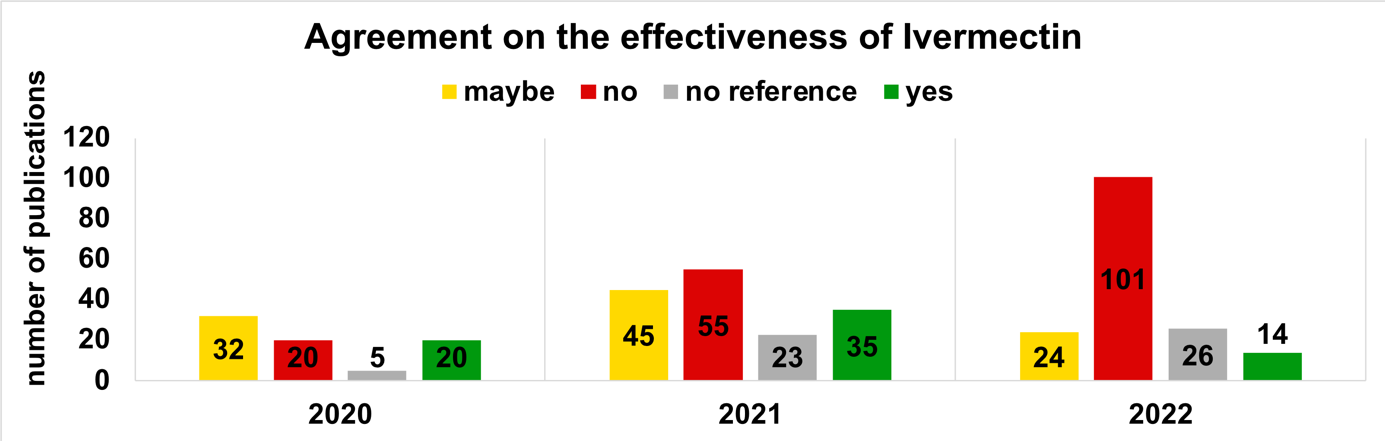


Supplemental Figure 9 This chart describes the opinion of the individual publications on the efficacy of ivermectin for COVID-19 between 2020 and 2022.


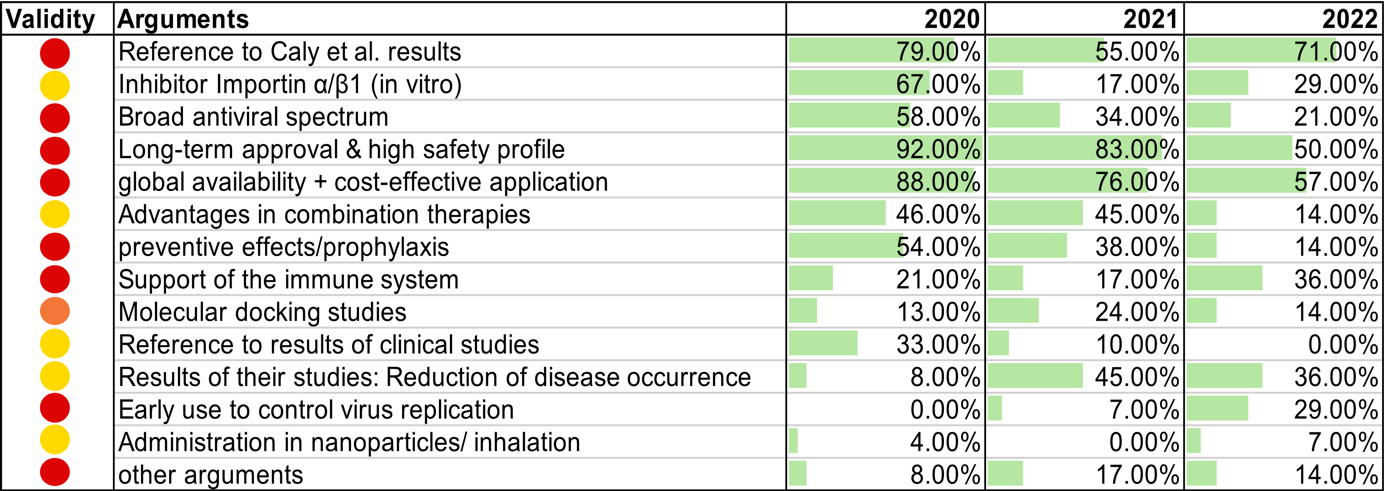


medium validity questionable validity low validity

Supplemental Figure 10 This table lists all the arguments that justify the prescription and use of ivermectin for COVID-19. The percentage use of these arguments in 2020, 2021 and 2022 is shown in the bar chart. The arguments are coded using a traffic light colour system according to their validity.


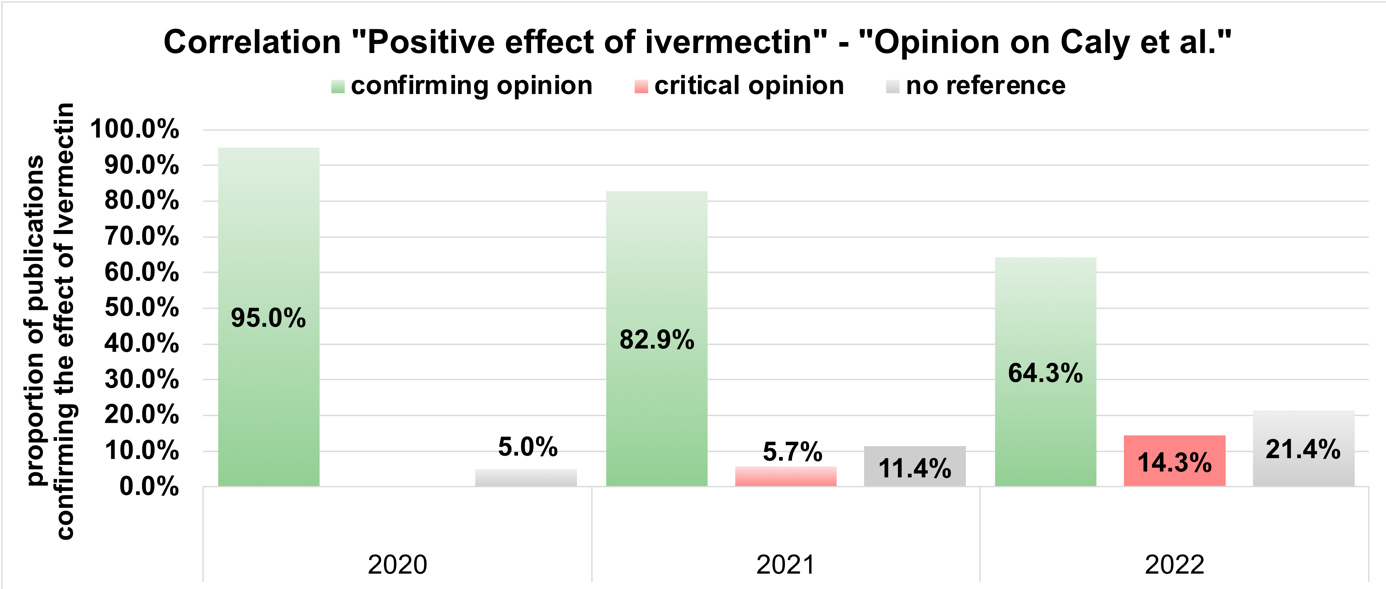


Supplemental Figure 11 This chart describes the correlation between the statement that ivermectin has a positive effect and the opinion of the individual publications on the study results of the in vitro study by Caly et al. The years 2020, 2021 and 2022 are shown. The proportion of publications confirming the effect of ivermectin is shown on the y-axis.


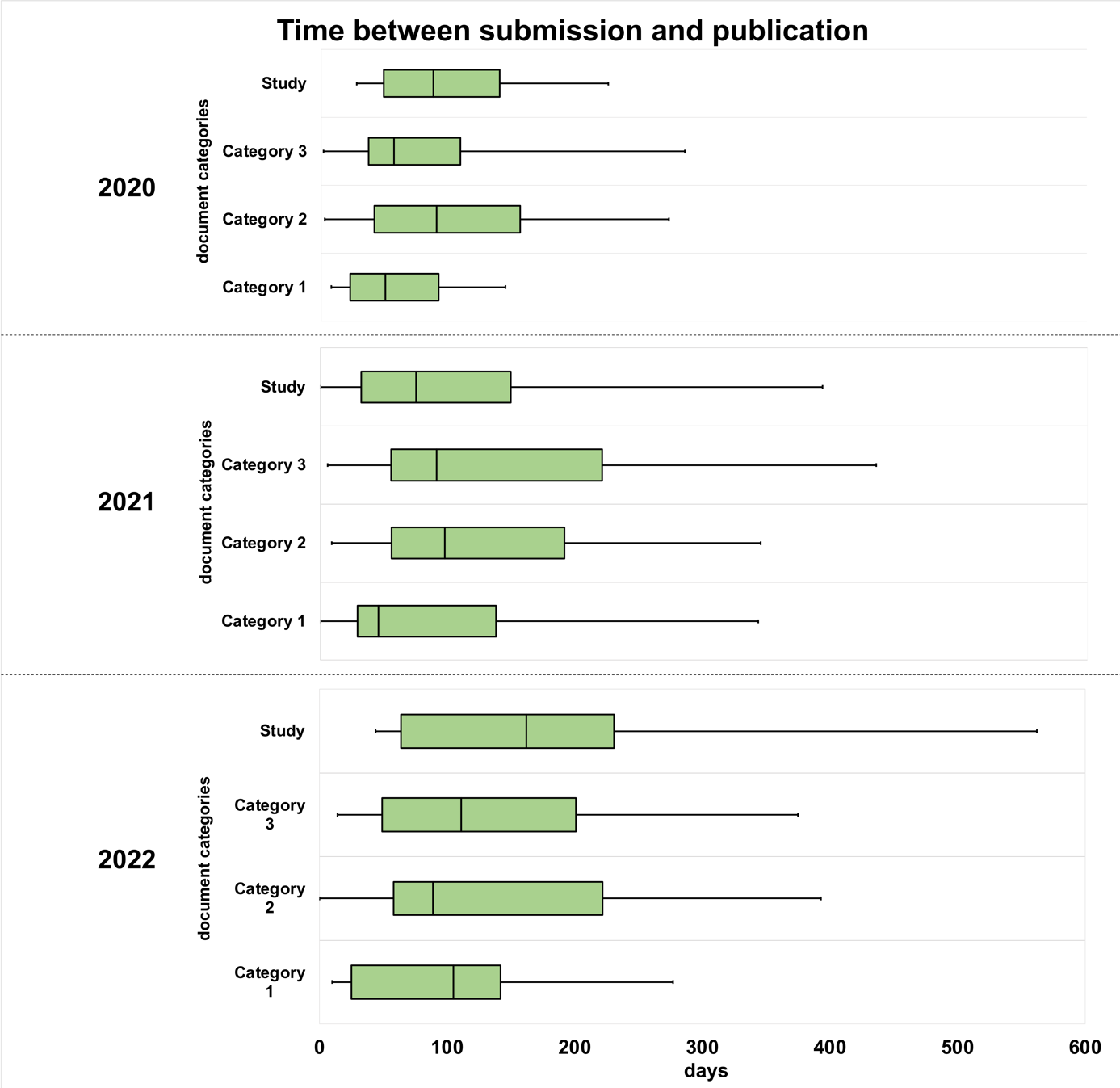


Supplemental Figure 12 This chart uses boxplots to show the time between submission and acceptance for publication, divided into the three categories according to document type (see Fig. 5) and studies. The x-axis describes the time in days. On the y-axis the categories are subordinated into the years 2020, 2021 and 2022.

The ANOVA analysis showed that the duration between submission and publication (measured in days) differs significantly between the years 2020 - 2022 for category 3: F (2, 110) = 5.207, p = < .007. There is no statistically significant difference between the publication time of the years 2020 - 2022 in the category 1, category 2 and category ‘Study’: in category 1: F (2, 56) = 2.241, p < 0.116; in category 2: F (2, 102) = 1.271, p < 0.285; in category ‘Study’: F (2, 39) = 1.679, p < 0.200. The Bonferroni-corrected post-hoc analysis revealed several significant differences (p < 0.05) in the number of days to publication; in category 3 between 2020 and 2021 (MDiff = 52.01, 95%-CI [9.93, 94.10] and between 2020 and 2022 (MDiff = 47.82, 95%-CI [6.28, 89.37] (Supplemental Tables 5-7)


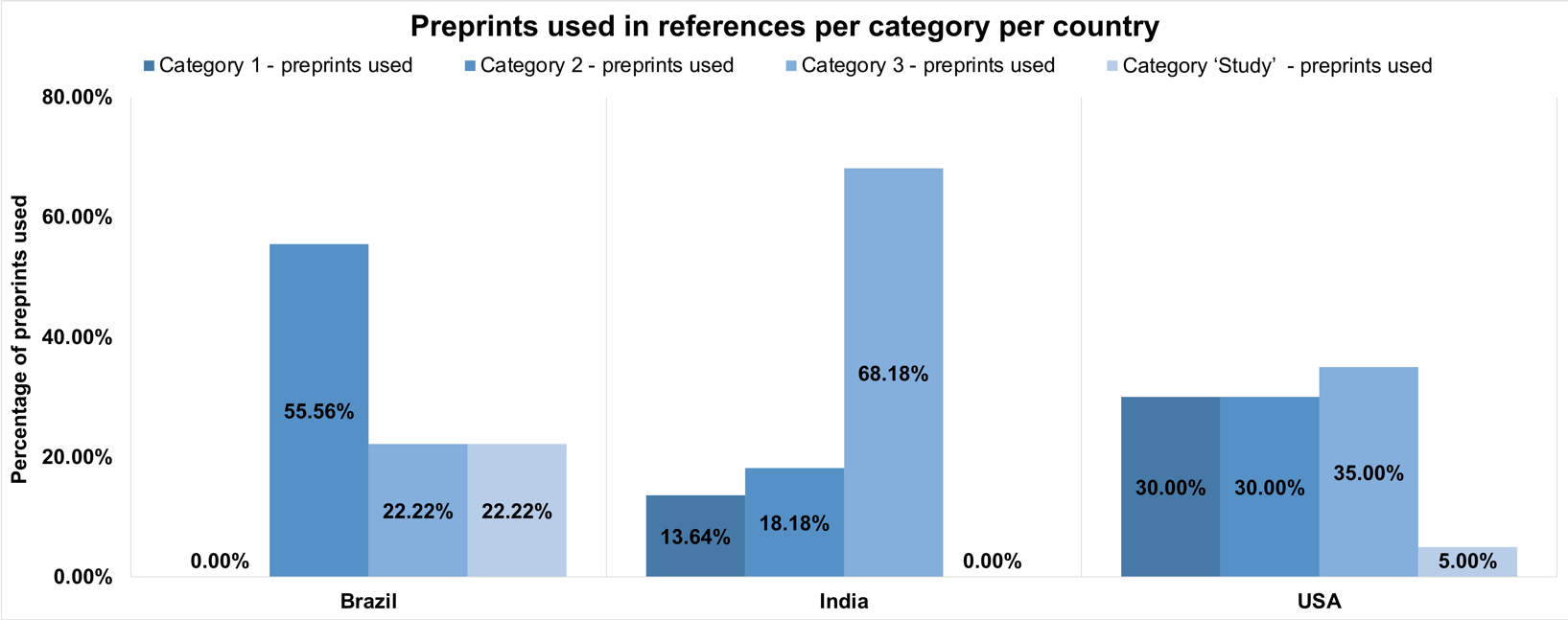
**Supplemental Figures (Pubmed analysis)**

Supplemental Figure 13 This chart shows a breakdown of the percentage distribution of the usage of preprints in the references per document category in Brazil, India and the USA.

**Supplemental Tables**

| # | Amino acid residues Amino acid residues in the receptor binding site (RBS) of the spike protein | Affinity | Binding energy | Investigation methods |
| --- | --- | --- | --- | --- |
| I | Leu492, Gln493, Gly496, Tyr505 | -9.0 kcal/mol | -30.60 kcal/mol | MD, MM-PBSA |
| J | Arg403, Ile418, Tyr489, Phe490 | -8.1 kcal/mol | / | MD, MM-PBSA |
| L | Tyr449, Tyr453, Leu455, Phe456, Ala475, Gly476, Phe486, Asn487, Tyr489, Gln493, Gly496, Gln498, Thr500, Asn501, Gly502, Tyr505 | -10.8697 kcal/mol | -89.360 kJ/mol | MM-PBSA |
| K | Leu492, Gln493, Gly496, Tyr505 | -9.0 kcal/mol | -22.4 kcal/mol (estimated using DFT) | MD, DFT |
| H | Tyr51, Ala54, Lys60, Pro66, Glu88, Arg90, Ser55, Asp87, Thr97, Thr182, Glu239, Lys245, Asp500, Thr183 | -372.99 kcal/mol | / | MD |
| G | Tyr453, Gln493, Asn501, Gly496 | -8.78 kcal/mol | / | Ensemble Docking, Reverse Docking |

Supplemental Table 1 The table shows all the molecular docking studies investigating the receptor binding site of the spike protein

| **Descriptives** | | | | | | | | |  |
| --- | --- | --- | --- | --- | --- | --- | --- | --- | --- |
|  | | N | Mean | Std. Deviation | Std. Error | 95% Confidence Interval for Mean | | Minimum | Maximum |
|  |  |  |  |  |  | Lower Bound | Upper Bound |  |  |
| Category 1 | 01/01/2020 | 37 | 54,16 | 41,309 | 6,791 | 40,39 | 67,94 | 8 | 153 |
|  | 01/01/2021 | 23 | 74,87 | 66,610 | 13,889 | 46,07 | 103,67 | 0 | 233 |
|  | 01/01/2022 | 27 | 108,37 | 74,505 | 14,339 | 78,90 | 137,84 | 10 | 308 |
|  | Total | 87 | 76,46 | 63,733 | 6,833 | 62,88 | 90,04 | 0 | 308 |
| Category 2 | 01/01/2020 | 18 | 83,28 | 52,530 | 12,381 | 57,16 | 109,40 | 3 | 182 |
|  | 01/01/2021 | 58 | 104,59 | 62,774 | 8,243 | 88,08 | 121,09 | 9 | 300 |
|  | 01/01/2022 | 62 | 141,65 | 84,346 | 10,712 | 120,23 | 163,07 | 9 | 346 |
|  | Total | 138 | 118,46 | 75,080 | 6,391 | 105,82 | 131,09 | 3 | 346 |
| Category 3 | 01/01/2020 | 16 | 48,56 | 28,383 | 7,096 | 33,44 | 63,69 | 2 | 90 |
|  | 01/01/2021 | 48 | 125,94 | 89,797 | 12,961 | 99,86 | 152,01 | 14 | 354 |
|  | 01/01/2022 | 41 | 112,12 | 67,269 | 10,506 | 90,89 | 133,35 | 14 | 287 |
|  | Total | 105 | 108,75 | 78,732 | 7,684 | 93,52 | 123,99 | 2 | 354 |
| Study | 01/01/2020 | 9 | 73,22 | 36,650 | 12,217 | 45,05 | 101,39 | 28 | 137 |
|  | 01/01/2021 | 18 | 107,11 | 104,898 | 24,725 | 54,95 | 159,28 | 2 | 404 |
|  | 01/01/2022 | 23 | 139,78 | 96,731 | 20,170 | 97,95 | 181,61 | 37 | 400 |
|  | Total | 50 | 116,04 | 94,173 | 13,318 | 89,28 | 142,80 | 2 | 404 |

*Supplemental Table 2* The table shows the descriptive analysis of the Pubmed data of the publication time for categories 1 to 3 and years, 2020 – 2022, carried out with SPSS.

| **ANOVA** | | | | | | |
| --- | --- | --- | --- | --- | --- | --- |
|  | | Sum of Squares | df | Mean Square | F | Sig. |
| Category 1 | Between Groups | 45947,677 | 2 | 22973,839 | 6,361 | ,003 |
|  | Within Groups | 303371,932 | 84 | 3611,571 |  |  |
|  | Total | 349319,609 | 86 |  |  |  |
| Category 2 | Between Groups | 66772,366 | 2 | 33386,183 | 6,389 | ,002 |
|  | Within Groups | 705493,874 | 135 | 5225,881 |  |  |
|  | Total | 772266,239 | 137 |  |  |  |
| Category 3 | Between Groups | 72606,422 | 2 | 36303,211 | 6,473 | ,002 |
|  | Within Groups | 572069,140 | 102 | 5608,521 |  |  |
|  | Total | 644675,562 | 104 |  |  |  |
| Study | Between Groups | 30900,674 | 2 | 15450,337 | 1,799 | ,177 |
|  | Within Groups | 403655,246 | 47 | 8588,409 |  |  |
|  | Total | 434555,920 | 49 |  |  |  |

*Supplemental Table 3* The table shows the results of the Pubmed data of the ANOVA analysis for category 1 to 3, carried out with SPSS.

| **Multiple Comparisons** | | | | | | | |
| --- | --- | --- | --- | --- | --- | --- | --- |
| **Bonferroni** | | | | | | | |
| Dependent Variable | (I) Year | (J) Year | Mean Difference (I-J) | Std. Error | Sig. | 95% Confidence Interval | |
|  |  |  |  |  |  | Lower Bound | Upper Bound |
| Category 1 | 01/01/2020 | 01/01/2021 | -20,707 | 15,957 | ,594 | -59,69 | 18,27 |
|  |  | 01/01/2022 | -54,208^*^ | 15,211 | ,002 | -91,37 | -17,05 |
|  | 01/01/2021 | 01/01/2020 | 20,707 | 15,957 | ,594 | -18,27 | 59,69 |
|  |  | 01/01/2022 | -33,501 | 17,052 | ,158 | -75,16 | 8,16 |
|  | 01/01/2022 | 01/01/2020 | 54,208^*^ | 15,211 | ,002 | 17,05 | 91,37 |
|  |  | 01/01/2021 | 33,501 | 17,052 | ,158 | -8,16 | 75,16 |
| Category 2 | 01/01/2020 | 01/01/2021 | -21,308 | 19,505 | ,830 | -68,59 | 25,97 |
|  |  | 01/01/2022 | -58,367^*^ | 19,355 | ,009 | -105,29 | -11,45 |
|  | 01/01/2021 | 01/01/2020 | 21,308 | 19,505 | ,830 | -25,97 | 68,59 |
|  |  | 01/01/2022 | -37,059^*^ | 13,206 | ,017 | -69,07 | -5,05 |
|  | 01/01/2022 | 01/01/2020 | 58,367^*^ | 19,355 | ,009 | 11,45 | 105,29 |
|  |  | 01/01/2021 | 37,059^*^ | 13,206 | ,017 | 5,05 | 69,07 |
| Category 3 | 01/01/2020 | 01/01/2021 | -77,375^*^ | 21,619 | ,002 | -130,00 | -24,75 |
|  |  | 01/01/2022 | -63,559^*^ | 22,075 | ,015 | -117,29 | -9,83 |
|  | 01/01/2021 | 01/01/2020 | 77,375^*^ | 21,619 | ,002 | 24,75 | 130,00 |
|  |  | 01/01/2022 | 13,816 | 15,926 | 1,000 | -24,95 | 52,58 |
|  | 01/01/2022 | 01/01/2020 | 63,559^*^ | 22,075 | ,015 | 9,83 | 117,29 |
|  |  | 01/01/2021 | -13,816 | 15,926 | 1,000 | -52,58 | 24,95 |
| Study | 01/01/2020 | 01/01/2021 | -33,889 | 37,834 | 1,000 | -127,82 | 60,04 |
|  |  | 01/01/2022 | -66,560 | 36,437 | ,222 | -157,02 | 23,90 |
|  | 01/01/2021 | 01/01/2020 | 33,889 | 37,834 | 1,000 | -60,04 | 127,82 |
|  |  | 01/01/2022 | -32,671 | 29,164 | ,805 | -105,08 | 39,73 |
|  | 01/01/2022 | 01/01/2020 | 66,560 | 36,437 | ,222 | -23,90 | 157,02 |
|  |  | 01/01/2021 | 32,671 | 29,164 | ,805 | -39,73 | 105,08 |
| *. The mean difference is significant at the 0.05 level. | | | | | | | |
| *Supplemental Table 4* The table shows the results of the Pubmed data of the post hoc analysis for category 1 to 3, carried out with SPSS. | | | | | | | |

| **Descriptives** | | | | | | | | | |
| --- | --- | --- | --- | --- | --- | --- | --- | --- | --- |
|  | | N | Mean | Std. Deviation | Std. Error | 95% Confidence Interval for Mean | | Minimum | Maximum |
|  |  |  |  |  |  | Lower Bound | Upper Bound |  |  |
| Category 1 | 2020 | 16 | 46,94 | 28,515 | 7,129 | 31,74 | 62,13 | 8 | 94 |
|  | 2021 | 26 | 80,58 | 75,383 | 14,784 | 50,13 | 111,02 | 0 | 297 |
|  | 2022 | 17 | 87,53 | 52,942 | 12,840 | 60,31 | 114,75 | 10 | 172 |
|  | Total | 59 | 73,46 | 60,891 | 7,927 | 57,59 | 89,33 | 0 | 297 |
| Category 2 | 2020 | 16 | 84,31 | 51,887 | 12,972 | 56,66 | 111,96 | 3 | 182 |
|  | 2021 | 40 | 96,30 | 52,210 | 8,255 | 79,60 | 113,00 | 9 | 247 |
|  | 2022 | 49 | 110,63 | 72,138 | 10,305 | 89,91 | 131,35 | 0 | 304 |
|  | Total | 105 | 101,16 | 62,509 | 6,100 | 89,06 | 113,26 | 0 | 304 |
| Category 3 | 2020 | 26 | 63,15 | 47,017 | 9,221 | 44,16 | 82,14 | 2 | 228 |
|  | 2021 | 42 | 115,17 | 79,702 | 12,298 | 90,33 | 140,00 | 6 | 344 |
|  | 2022 | 45 | 110,98 | 69,679 | 10,387 | 90,04 | 131,91 | 14 | 264 |
|  | Total | 113 | 101,53 | 71,929 | 6,767 | 88,12 | 114,94 | 2 | 344 |
| Study | 2020 | 9 | 73,11 | 36,498 | 12,166 | 45,06 | 101,17 | 28 | 137 |
|  | 2021 | 16 | 99,44 | 93,193 | 23,298 | 49,78 | 149,10 | 0 | 318 |
|  | 2022 | 17 | 136,65 | 100,493 | 24,373 | 84,98 | 188,32 | 44 | 400 |
|  | Total | 42 | 108,86 | 89,519 | 13,813 | 80,96 | 136,75 | 0 | 400 |

Supplemental Table 5 The table shows the descriptive analysis of the Clarivate data of the publication time for categories 1 to 3 and years, 2020 – 2022, carried out with SPSS.

| **ANOVA** | | | | | | |
| --- | --- | --- | --- | --- | --- | --- |
|  | | Sum of Squares | df | Mean Square | F | Sig. |
| Category 1 | Between Groups | 15937,125 | 2 | 7968,563 | 2,241 | ,116 |
|  | Within Groups | 199109,519 | 56 | 3555,527 |  |  |
|  | Total | 215046,644 | 58 |  |  |  |
| Category 2 | Between Groups | 9883,022 | 2 | 4941,511 | 1,271 | ,285 |
|  | Within Groups | 396481,225 | 102 | 3887,071 |  |  |
|  | Total | 406364,248 | 104 |  |  |  |
| Category 3 | Between Groups | 50117,946 | 2 | 25058,973 | 5,207 | ,007 |
|  | Within Groups | 529344,196 | 110 | 4812,220 |  |  |
|  | Total | 579462,142 | 112 |  |  |  |
| Study | Between Groups | 26048,434 | 2 | 13024,217 | 1,679 | ,200 |
|  | Within Groups | 302510,709 | 39 | 7756,685 |  |  |
|  | Total | 328559,143 | 41 |  |  |  |

Supplemental Table 6 The table shows the results of the Clarivate data of the ANOVA analysis for category 1 to 3, carried out with SPSS.

| **Multiple Comparisons** | | | | | | | |
| --- | --- | --- | --- | --- | --- | --- | --- |
| **Bonferroni** | | | | | | | |
| Dependent Variable | (I) Year | (J) Year | Mean Difference (I-J) | Std. Error | Sig. | 95% Confidence Interval | |
|  |  |  |  |  |  | Lower Bound | Upper Bound |
| Category 1 | 2020 | 2021 | -33,639 | 18,947 | ,244 | -80,40 | 13,12 |
|  |  | 2022 | -40,592 | 20,769 | ,167 | -91,85 | 10,67 |
|  | 2021 | 2020 | 33,639 | 18,947 | ,244 | -13,12 | 80,40 |
|  |  | 2022 | -6,952 | 18,598 | 1,000 | -52,85 | 38,95 |
|  | 2022 | 2020 | 40,592 | 20,769 | ,167 | -10,67 | 91,85 |
|  |  | 2021 | 6,952 | 18,598 | 1,000 | -38,95 | 52,85 |
| Category 2 | 2020 | 2021 | -11,987 | 18,442 | 1,000 | -56,88 | 32,90 |
|  |  | 2022 | -26,320 | 17,952 | ,437 | -70,02 | 17,38 |
|  | 2021 | 2020 | 11,987 | 18,442 | 1,000 | -32,90 | 56,88 |
|  |  | 2022 | -14,333 | 13,286 | ,850 | -46,67 | 18,01 |
|  | 2022 | 2020 | 26,320 | 17,952 | ,437 | -17,38 | 70,02 |
|  |  | 2021 | 14,333 | 13,286 | ,850 | -18,01 | 46,67 |
| Category 3 | 2020 | 2021 | -52,013^*^ | 17,311 | ,010 | -94,10 | -9,93 |
|  |  | 2022 | -47,824^*^ | 17,089 | ,018 | -89,37 | -6,28 |
|  | 2021 | 2020 | 52,013^*^ | 17,311 | ,010 | 9,93 | 94,10 |
|  |  | 2022 | 4,189 | 14,883 | 1,000 | -31,99 | 40,37 |
|  | 2022 | 2020 | 47,824^*^ | 17,089 | ,018 | 6,28 | 89,37 |
|  |  | 2021 | -4,189 | 14,883 | 1,000 | -40,37 | 31,99 |
| Study | 2020 | 2021 | -26,326 | 36,697 | 1,000 | -118,13 | 65,48 |
|  |  | 2022 | -63,536 | 36,306 | ,264 | -154,36 | 27,29 |
|  | 2021 | 2020 | 26,326 | 36,697 | 1,000 | -65,48 | 118,13 |
|  |  | 2022 | -37,210 | 30,677 | ,697 | -113,95 | 39,53 |
|  | 2022 | 2020 | 63,536 | 36,306 | ,264 | -27,29 | 154,36 |
|  |  | 2021 | 37,210 | 30,677 | ,697 | -39,53 | 113,95 |
| *. The mean difference is significant at the 0.05 level. | | | | | | | |

Supplemental Table 7 The table shows the results of the Clarivate data of the post hoc analysis for category 1 to 3, carried out with SPSS.

| # | Author (year) | Title | Opinion on Caly et al |
| --- | --- | --- | --- |
| 1 | Samaddar et al. (2020) | Pathophysiology and Potential Therapeutic Candidates for COVID-19: A Poorly Understood Arena | Results adopted |
| 2 | Rocha Formiga et al. (2020) | Ivermectin: an award-winning drug with expected antiviral activity against COVID-19 | Results criticized |
| 3 | Martin et al. (2020) | Ivermectin: An Anthelmintic, an Insecticide, and Much More | Results criticized |
| 4 | Bray et al. (2020) | Ivermectin and COVID-19: A report in Antiviral Research, widespread interest, an FDA warning, two letters to the editor and the authors' responses | Results criticized |
| 5 | Gouvea Dos Santos (2020) | Natural history of COVID-19 and current knowledge on treatment therapeutic options | Results criticized |
| 6 | Chan et al. (2020) | Pandemic COVID-19: Current status and challenges of antiviral therapies | Results criticized |
| 7 | Schmith et al. (2020) | The Approved Dose of Ivermectin Alone is not the Ideal Dose for the Treatment of COVID-19 | Results criticized |
| 8 | Khana et al. (2020) | Reply to “Ivermectin Treatment May Improve the Prognosis of Patients With COVID-19” | Results criticized |
| 9 | Wang, Yang  (2020) | Turning the Tide: Natural Products and Natural-Product-Inspired Chemicals as Potential Counters to SARS-CoV-2 Infection | Results criticized |
| 10 | Marcolino, Pimentel, Barão (2020) | What to expect from different drugs used in the treatment of COVID-19: A study on applications and in vivo and in vitro results | Results criticized |
| 11 | Chaccour et al. (2020) | Nebulized ivermectin for COVID‐19 and other respiratory diseases, a proof of concept, dose‐ranging study in rats | Results criticized |
| 12 | Peña-Silva et al. (2020) | Pharmacokinetic considerations on the repurposing of ivermectin for treatment of COVID-19 | Results criticized |
| 13 | Gupta (2020) | Progress in Studies on Structural and Remedial Aspects of Newly Born Coronavirus, SARS-CoV-2 | Results criticized |
| 14 | Segura-Aguilar, Tizabi (2020) | Ivermectin as a potential therapeutic in COVID-19 | Results criticized |
| 15 | López Reboiro et al. (2020) | Ivermectin in COVID-19. Argumentun ad ignorantiam? | Results criticized |
| 16 | Hossen et al. (2020) | A Review on Current Repurposing Drugs for the Treatment of COVID-19: Reality and Challenges | Results criticized |
| 17 | Lehrer, Rheinstein et al. (2020) | Ivermectin Docks to the SARS-CoV-2 Spike Receptor-binding Domain Attached to ACE2 | Results criticized |
| 18 | Elkholy et al. (2020) | Ivermectin: A Closer Look at a Potential Remedy | Results criticized |
| 19 | Heidary, Gharebaghi (2020) | Ivermectin: a systematic review from antiviral effects to COVID-19 complementary regimen | Results criticized |
| 20 | Drożdża et al. (2020) | FDA approved drugs with pharmacotherapeutic potential for SARS-CoV-2 (COVID-19) therapy | Results adopted |
| 21 | Patrì et al. (2020) | Hydroxychloroquine and Ivermectin: A synergistic combination for COVID-19 chemoprophylaxis and treatment? | Results adopted |
| 22 | Hellwig et al. (2020) | A COVID-19 prophylaxis? Lower incidence associated with prophylactic administration of ivermectin | Results adopted |
| 23 | Perez-Garcia et al. (2020) | Ivermectin: repurposing a multipurpose drug for Venezuela's humanitarian crisis | Results adopted |
| 24 | Alonso et al. (2020) | Repurposing of host-based therapeutic agents for the treatment of coronavirus disease 2019 (COVID-19): a link between antiviral and anticancer mechanisms? | Results adopted |
| 25  *Supplemental Table 8* The table shows a list of all publications 2020 – 2022 and their opinion on the publication of Caly et al. | Kumar et al. (2020) | A chronicle of SARS-CoV-2: Part-I - Epidemiology, diagnosis, prognosis, transmission and treatment | Results adopted |
| 26 | Ahmed et al. (2020) | A five-day course of ivermectin for the treatment of COVID-19 may reduce the duration of illness | Results adopted |
| 27 | Rajter et al. (2020) | Use of Ivermectin Is Associated With Lower Mortality in Hospitalized Patients With Coronavirus Disease 2019: The Ivermectin in COVID Nineteen Study | Results adopted |
| 28 | Taguchi, Turki (2020) | A new advanced in silico drug discovery method for novel coronavirus (SARS-CoV-2) with tensor decomposition-based unsupervised feature extraction | Results adopted |
| 29 | Kelleni (2020) | Nitazoxanide/azithromycin combination for COVID-19: A suggested new protocol for early management | Results adopted |
| 30 | Yuan et al. (2020) | Discovery of the FDA-approved drugs bexarotene, cetilistat, diiodohydroxyquinoline, and abiraterone as potential COVID-19 treatments with a robust two-tier screening system | Results adopted |
| 31 | Beltrão Molento (2020) | COVID-19 and the rush for self-medication and self-dosing with ivermectin: A word of caution | Results adopted |
| 32 | Pandey et al. (2020) | Ivermectin in COVID-19: What do we know? | Results adopted |
| 33 | Parvez et al. (2020) | Prediction of potential inhibitors for RNA-dependent RNA polymerase of SARS-CoV-2 using comprehensive drug repurposing and molecular docking approach | Results adopted |
| 34 | Jans, Wagstaff (2020) | Ivermectin as a Broad-Spectrum Host-Directed Antiviral: The Real Deal? | Results adopted |
| 35 | Panoutsopoulos (2020) | Known drugs and small molecules in the battle for COVID-19 treatment | Results adopted |
| 36 | Sharun et al. (2020) | Ivermectin, a new candidate therapeutic against SARS-CoV-2/COVID-19 | Results adopted |
| 37 | Li, Zhao, Zhan  (2020) | Quantitative proteomics reveals a broad‐spectrum antiviral property of ivermectin, benefiting for COVID‐19 treatment | Results adopted |
| 38 | Khan et al. (2020) | Ivermectin Treatment May Improve the Prognosis of Patients With COVID-19 | Results adopted |
| 39 | Perišić (2020) | Recognition of Potential COVID-19 Drug Treatments through the Study of Existing Protein-Drug and Protein-Protein Structures: An Analysis of Kinetically Active Residues | Results adopted |
| 40 | Hussman (2020) | Cellular and Molecular Pathways of COVID-19 and Potential Points of Therapeutic Intervention | Results adopted |
| 41 | Gupta, Rana (2020) | Ivermectin, Famotidine, and Doxycycline: A Suggested Combinatorial Therapeutic for the Treatment of COVID-19 | Results adopted |
| 42 | Surnar et al. (2020) | Clinically Approved Antiviral Drug in an Orally Administrable Nanoparticle for COVID-19 | Results adopted |
| 43 | Vanachayangkul et al. (2020) | Safety, Pharmacokinetics, and Activity of High-Dose Ivermectin and Chloroquine against the Liver Stage of Plasmodium cynomolgi Infection in Rhesus Macaques | Results adopted |
| 44 | Jean, Hsueh  (2020) | Old and re-purposed drugs for the treatment of COVID-19 | Results adopted |
| 45 | Low, Farouk, Lal (2020) | Drug Repositioning: New Approaches and Future Prospects for Life-Debilitating Diseases and the COVID-19 Pandemic Outbreak | Results adopted |
| 46 | Hanafy, Abd-Elsalam (2020) | Challenges in COVID-19 drug treatment in patients with advanced liver diseases: A hepatology perspective | Results adopted |
| 47 | Vital de Oliveiraa et al. (2020) | Repurposing approved drugs as inhibitors of SARS-CoV-2 S-protein from molecular modeling and virtual screening | Results adopted |
| 48 | Siddiquia et al. (2020) | Current status and strategic possibilities on potential use of combinational drug therapy against COVID-19 caused by SARS-CoV-2 | Results adopted |
| 49 | Kalhor et al. (2020) | Repurposing of the approved small molecule drugs in order to inhibit SARS-CoV-2 S protein and human ACE2 interaction through virtual screening approaches | Results adopted |
| 50 | Gupta et al. (2020) | Binding mechanism and structural insights into the identified protein target of COVID-19 and importin-a with in-vitro effective drug ivermectin | Results adopted |
| 51 | Azama et al. (2020) | An in-silico analysis of ivermectin interaction with potential SARS-CoV-2 targets and host nuclear importin alpha | Results adopted |
| 52 | Ali et al. (2020) | Treatment Options for COVID-19: A Review | Results adopted |
| 53 | Anastasiou et al. (2020) | In Vitro Data of Current Therapies for SARS-CoV-2 | Results adopted |
| 54 | Mittal (2020) | Inhaled route and anti-inflammatory action of ivermectin: Do they hold promise in fighting against COVID-19? | Results adopted |
| 55 | Horowitz, Freeman (2020) | Three novel prevention, diagnostic, and treatment options for COVID-19 urgently necessitating controlled randomized trials | Results adopted |
| 56 | Heimfarth et al. (2020) | IVERMECTIN: PANACEA OR TRUE PROMISE FOR COVID-19? | Results adopted |
| 57 | Camprubí et al. (2020) | Lack of efficacy of standard doses of ivermectin in severe COVID-19 patients | Results adopted |
| 58 | Jerman et al. (2020) | Development of a Minimal Physiologically-Based Pharmacokinetic Model to Simulate Lung Exposure in Humans Following Oral Administration of Ivermectin for COVID-19 Drug Repurposing | Results adopted |
| 59 | Merone, Finlay (2020) | Pandemic and promise: progress towards finding an effective treatment for Novel Coronavirus 19 | Results adopted |
| 60 | Trivedi, Verma, Kumar (2020) | Possible treatment and strategies for COVID-19: review and assessment | Results adopted |
| 61 | Chaccour et al. (2020) | Ivermectin and COVID-19: Keeping Rigor in Times of Urgency | Results adopted |
| 62 | Tapia (2020) | COVID-19 and Fake News in the Dominican Republic | Results adopted |
| 63 | Jans, Wagstaff (2020) | The broad spectrum host-directed agent ivermectin as an antiviral for SARS-CoV-2 ? | Results adopted |
| 64 | Gupta, Sahoo, Singh (2020) | Ivermectin: potential candidate for the treatment of Covid 19 | Results adopted |
| 65 | Padhy et al. (2020) | Therapeutic potential of ivermectin as add-on treatment in COVID 19: A systematic review and meta-analysis | Results adopted |
| 66 | Mishra, Tripathi (2020) | One year update on the COVID-19 pandemic: Where are we now? | Results adopted |
| 67 | Rizzo (2020) | Ivermectin, antiviral properties and COVID-19: a possible new mechanism of action | Results adopted |
| 68 | Khan et al. (2020) | Toward Preparing a Knowledge Base to Explore Potential Drugs and Biomedical Entities Related to COVID-19: Automated Computational Approach | Results adopted |
| 69 | Hamed (2020) | An overview on COVID-19: reality and expectation | Results adopted |
| 70 | Galvao de Castro, Gregianin, Burger (2020) | Continuous high-dose ivermectin appears to be safe in patients with acute myelogenous leukemia and could inform clinical repurposing for COVID-19 infection | Results adopted |
| 71 | Yavuz, Ünal (2020) | Antiviral treatment of COVID-19 | Results adopted |
| 72 | Vallejos et al. (2020) | Ivermectin to prevent hospitalizations in patients with COVID-19 (IVERCOR-COVID19): a structured summary of a study protocol for a randomized controlled trial | Results adopted |
| 73 | Banerjee et al. (2020) | The Battle against COVID 19 Pandemic: What we Need to Know Before we "Test Fire" Ivermectin | Results adopted |
| 74 | Paumgartten, de Oliveira  (2020) | Off label, compassionate and irrational use of medicines in Covid-19 pandemic, health consequences and ethical issues | Results adopted |
| 75 | Akshayaa et al. (2020) | Data on known anti-virals in combating Covid-19 | Results adopted |
| 76 | Khadka et al. (2020) | Repurposing Drugs for COVID-19: An Approach for Treatment in the Pandemic | Results adopted |
| 77 | Guerrero et al. (2020) | COVID-19: The Ivermectin African Enigma | Results adopted |
| 78 | Peron et al. (2020) | COVID-19 Pandemic and Dysbiosis: Can the Ivermectin Hysteria Lead to an Increase of Autoimmune Neuroinflammatory Diseases? | Results adopted |
| 79 | Vora et al. (2020) | White paper on Ivermectin as a potential therapy for COVID-19 | Results adopted |
| 80 | Daghir Janabi (2020) | Effective Anti-SARS-CoV-2 RNA Dependent RNA Polymerase Drugs Based on Docking Methods: The Case of Milbemycin, Ivermectin, and Baloxavir Marboxil | Results adopted |
| 81 | Dixit et al. (2020) | Ivermectin: Potential Role as Repurposed Drug for COVID-19 | Results adopted |
| 82 | Choudhary, Sharma (2020) | Potential use of hydroxychloroquine, ivermectin and azithromycin drugs in fighting COVID-19: trends, scope and relevance | Results adopted |
| 83 | Khan et al. (2020) | Diagnostic approaches and potential therapeutic options for coronavirus disease 2019 | Results adopted |
| 84 | García-Álvarez, García-Vigil (2020) | Guidelines for clinical management of SARS-CoV-2 infection | Results adopted |
| 85 | Cedillo-Alvarez et al. (2020) | COVID-19: a basic approach to understanding potential treatments | Results adopted |
| 86 | Agarwal et al. (2020) | A living WHO guideline on drugs for covid-19 | no reference |
| 87 | Torres-Atencio et al. (2020) | COVID-19: Panama stockpiles unproven drugs | no reference |
| 88 | Nunes Szente Fonseca et al. (2020) | Risk of hospitalization for Covid-19 outpatients treated with various drug regimens in Brazil: Comparative analysis | no reference |
| 89 | Kim et al (2020) | Comparative efficacy and safety of pharmacological interventions for the treatment of COVID-19: A systematic review and network meta-analysis | no reference |
| 90 | Chuang et al. (2020) | Successful treatment of tocilizumab and ivermectin for a patient with ARDS due to COVID-19 | no reference |
| 91 | Marchese et al. (2020) | Strongyloides infection manifested during immunosuppressive therapy for SARS-CoV-2 pneumonia | no reference |
| 92 | Idda, Soru, Floris (2020) | Overview of the First 6 Months of Clinical Trials for COVID-19 Pharmacotherapy: The Most Studied Drugs | no reference |
| 93 | Procterr et al. (2020) | Clinical outcomes after early ambulatory multidrug therapy for high-risk SARS-CoV-2 (COVID-19) infection | no reference |
| 94 | Montoya et al. (2020) | COVID-19 in pediatric cancer patients in a resource-limited setting: National data from Peru | no reference |
| 95 | Mohapatra et al. (2020) | Computational investigations of three main drugs and their comparison with synthesized compounds as potent inhibitors of SARS-CoV-2 main protease (Mpro): DFT, QSAR, molecular docking, and in silico toxicity analysis | no reference |
| 96 | de Carvalho (2020) | COVID-19 in Still’s disease | no reference |
| 97 | Wamae  (2020) | Mass Drug Administration and Worms Experience in Africa: Envisage Repurposing Ivermectin for SARS-COV-2 | no reference |
| 98 | López Reboiro et al. (2020) | Reply to- Ivermectin in COVID-19. Argumentum ad ignorantiam? | no reference |
| 99 | Chaccour et al. (2020) | The SARS-CoV-2 Ivermectin Navarra-ISGlobal Trial (SAINT) to Evaluate the Potential of Ivermectin to Reduce COVID-19 Transmission in low risk, non-severe COVID-19 patients in the first 48 hours after symptoms onset: A structured summary of a study protocol for a randomized control pilot trial | no reference |
| 100 | Suzuki et al. (2020) | Severe Coronavirus Disease 2019 That Recovered from Respiratory Failure by Treatment That Included High-dose Intravenous Immunoglobulin | no reference |
| 101 | Turkia (2020) | The History of Methylprednisolone, Ascorbic Acid, Thiamine, and Heparin Protocol and I- MASK+ Ivermectin Protocol for COVID-19 | no reference |
| 102 | Malik et al. (2020) | Clinical Presentation, Management and In-Hospital Outcome of Healthcare Personnel With COVID-19 Disease | no reference |
| 103 | Wadvalla (2021) | Covid-19: Ivermectin’s politicisation is a warning sign for doctors | Results criticized |
| 104 | Vallejos et al. (2021) | Ivermectin to prevent hospitalizations in patients with COVID-19 (IVERCOR-COVID19) a randomized, double-blind, placebo- controlled trial | Results criticized |
| 105 | Temple, Hoang, Hendrickson (2021) | Toxic Effects from Ivermectin Use Associated with Prevention and Treatment of Covid-19 | Results criticized |
| 106 | Tawfeek et al. (2021) | Protective effect of vitamin C against ivermectin induced nephrotoxicity in different age groups of male wistar rats: bio-histopathological study | Results criticized |
| 107 | Siedner (2021) | Ivermectin for the Treatment of COVID-19 Disease: Too Good to Pass Up or Too Good to Be True? | Results criticized |
| 108 | Shukla, Misra (2021) | > | Results criticized |
| 109 | Rothrock et al. (2021) | Meta-Analyses Do Not Establish Improved Mortality With Ivermectin Use in COVID-19 | Results criticized |
| 110 | Reardon (2021) | Flawed ivermectin preprint highlights challenges of COVID drug studies | Results criticized |
| 111 | Popp et al. (2021) | Ivermectin for preventing and treating COVID-19 (Review) | Results criticized |
| 112 | Popp et al. (2021) | Evidence on the efficacy of ivermectin for COVID-19: another story of apples and oranges | Results criticized |
| 113 | Pecho-Silva et al. (2021) | Non-recommended medical interventions and their possible harm in patients with COVID-19 | Results criticized |
| 114 | Payne et al. (2021) | Evidence-based approach to early outpatient treatment of SARS-CoV-2 (COVID-19) infection | Results criticized |
| 115 | Patil, Verma, Masand (2021) | Prospective mode of action of Ivermectin: SARS-CoV-2. | Results criticized |
| 116 | Parrish et al. (2021) | Meta-analytic magic, ivermectin, and socially responsible reporting | **Results criticized** |
| 117 | Mouffaka et al. (2021) | Recent advances in management of COVID-19: A review | Results criticized |
| 118 | Mohan et al (2021) | Single-dose oral ivermectin in mild and moderate COVID-19 (RIVET-COV): A single-centre randomized, placebo-controlled trial | Results criticized |
| 119 | Mody et al (2021) | Identification of 3-chymotrypsin like protease (3CLPro) inhibitors as potential anti-SARS-CoV-2 agents | Results criticized |
| 120 | Mahmud et al. (2021) | Ivermectin in combination with doxycycline for treating COVID-19 symptoms: a randomized trial | Results criticized |
| 121 | Low, Yip, Lal (2021) | Repositioning Ivermectin for Covid-19 treatment: Molecular mechanisms of action against SARS-CoV-2 replication | Results criticized |
| 122 | Lawrence et al. (2021) | The lesson of ivermectin: meta-analyses based on summary data alone are inherently unreliable | Results criticized |
| 123 | Lasco, Yu (2021) | Pharmaceutical messianism and the COVID-19 pandemic | Results criticized |
| 124 | Khandelwal, Singh, Jamil (2021) | Ivermectin as a multifaceted drug in COVID-19: Current insights | Results criticized |
| 125 | Izcovich et al. (2021) | Bias as a source of inconsistency in ivermectin trials for COVID-19: A systematic review. Ivermectin’s suggested benefits are mainly based on potentially biased results | Results criticized |
| 126 | Iwanaga et al. (2021) | A fatal case of COVID-19-associated invasive pulmonary aspergillosis | Results criticized |
| 127 | Gurung et al. (2021) | Molecular modelling studies unveil potential binding sites on human serum albumin for selected experimental and in silico COVID-19 drug candidate molecules | Results criticized |
| 128 | García-Lledó et al. (2021) | Pharmacological treatment of COVID-19: an opinion paper | Results criticized |
| 129 | Fittler et al. (2021) | Effect of Infodemic Regarding the Illegal Sale of Medications on the Internet: Evaluation of Demand and Online Availability of Ivermectin during the COVID-19 Pandemic | Results criticized |
| 130 | Elalfy et al. (2021) | Effect of a combination of nitazoxanide, ribavirin, and ivermectin plus zinc supplement (MANS.NRIZ study) on the clearance of mild COVID‐19 | Results criticized |
| 131 | Echeverría-Esnal, Grau (2021) | Ivermectin: a pathway out of the pandemic or another dead end? | Results criticized |
| 132 | Dyer (2021) | Covid-19: Hospital may cease giving patient ivermectin, US court rules, as prescriptions soar | Results criticized |
| 133 | Drozdzal et al. (2021) | An update on drugs with therapeutic potential for SARS-CoV-2 (COVID-19) treatment | Results criticized |
| 134 | Dietl, Frick (2021) | Channels and Transporters of the Pulmonary Lamellar Body in Health and Disease | Results criticized |
| 135 | Deng et al. (2021) | Efficacy and safety of ivermectin for the treatment of COVID-19: a systematic review and meta-analysis | Results criticized |
| 136 | Deng et al. (2021) | Caution should be exercised when assessing ivermectin for the treatment of COVID‐19 in systematic reviews | Results criticized |
| 137 | Chaccour et al. (2021) | The effect of early treatment with ivermectin on viral load, symptoms and humoral response in patients with non-severe COVID-19: A pilot, double-blind, placebo-controlled, randomized clinical trial | Results criticized |
| 138 | Buonfrate, Bisoffi, (2021) | Standard Dose Ivermectin for COVID-19 | Results criticized |
| 139 | Bryant et al. (2021) | Ivermectin for Prevention and Treatment of COVID-19 Infection: A Systematic Review, Meta-analysis, and Trial Sequential Analysis to Inform Clinical Guidelines | Results criticized |
| 140 | Borges dos Santos (2021) | Regional drug information center disseminates educational materials related to the COVID-19 pandemic | Results criticized |
| 141 | Beltrã̃o Molento (2021) | Ivermectin against COVID-19: The unprecedented consequences in Latin America | Results criticized |
| 142 | Bartoleetti et al. (2021) | ESCMID COVID-19 living guidelines: drug treatment and clinical management | Results criticized |
| 143 | Alvarez-Moreno et al. (2021) | Long-term consequences of the misuse of ivermectin data | Results criticized |
| 144 | Abd‐Elsalam et al. (2021) | Clinical study evaluating the efficacy of ivermectin in COVID‐19 treatment: A randomized controlled study | Results criticized |
| 145 | Rensburg et al. (2021) | Ivermectin for COVID-19: Promising but not yet conclusive | Results criticized |
| 146 | Zein et al. (2021) | Ivermectin and mortality in patients with COVID-19: A systematic review, meta-analysis, and meta-regression of randomized controlled trials | Results adopted |
| 147 | Zaidi, Dehgani-Mobaraki (2021) | The mechanisms of action of ivermectin against SARS-CoV-2—an extensive review | Results adopted |
| 148 | Yuce et al. (2021) | Repurposing of FDA-approved drugs against active site and potential allosteric drug-binding sites of COVID-19 main protease | Results adopted |
| 149 | Younis et al. (2021) | COVID‐19: potential therapeutics for pediatric patients | Results adopted |
| 150 | Yadav et al. (2021) | Antiviral treatment in COVID-19: which is the most promising? - a narrative review | Results adopted |
| 151 | WiWanitkit (2021) | Ivermectin and COVID-19 | Results adopted |
| 152 | Wietzikoski Lovato et al. (2021) | Repurposing Drugs for the Management of Patients with Confirmed Coronavirus Disease 2019 (COVID-19) | Results adopted |
| 153 | Wehbe et al. (2021) | Repurposing Ivermectin for COVID-19: Molecular Aspects and Therapeutic Possibilities | Results adopted |
| 154 | Udofia et al. (2021) | In silico studies of selected multi-drug targeting against 3CLpro and nsp12 RNA-dependent RNA-polymerase proteins of SARS-CoV-2 and SARS-CoV | Results adopted |
| 155 | Uddin et al. (2021) | Potential Drugs for the Treatment of COVID-19: Synthesis, Brief History and Application | Results adopted |
| 156 | Toro et al. (2021) | Effect of Ivermectin and Atorvastatin on Nuclear Localization of Importin Alpha and Drug Target Expression Profiling in Host Cells from Nasopharyngeal Swabs of SARS-CoV-2- Positive Patients | Results adopted |
| 157 | Telbisz et al. (2021) | Interactions of Potential Anti-COVID-19 Compounds with Multispecific ABC and OATP Drug Transporters | Results adopted |
| 158 | Tan et al. (2021) | Combination Treatment With Remdesivir and Ivermectin Exerts Highly Synergistic and Potent Antiviral Activity Against Murine Coronavirus Infection | Results adopted |
| 159 | Taher, Tik Susanti (2021) | Drugs intervention study in COVID-19 management | Results adopted |
| 160 | Shimizu et al. (2021) | Ivermectin administration is associated with lower gastrointestinal complications and greater ventilator-free days in ventilated patients with COVID-19: A propensity score analysis | Results adopted |
| 161 | Shahbaznejad et al. (2021) | Effects of Ivermectin in Patients With COVID-19: A Multicenter, Double-blind, Randomized, Controlled Clinical Trial | Results adopted |
| 162 | Shahbaznejad et al. (2021) | Response to Letter Regarding Article, “Effects of Ivermectin in Patients With COVID-19: A Multicenter, Double-Blind, Randomized, Controlled Clinical Trial” | Results adopted |
| 163 | Semiz (2021) | SIT1 transporter as a potential novel target in treatment of COVID-19 | Results adopted |
| 164 | Seeta et al. (2021) | Positive impact of oral hydroxychloroquine and povidone-iodine throat spray for COVID-19 prophylaxis: An open-label randomized trial | Results adopted |
| 165 | Sardana, Mathachan (2021) | Is there any prophylactic role for ivermectin in COVID-19—A literature summary | Results adopted |
| 166 | Saha, Raihan (2021) | The binding mechanism of ivermectin and levosalbutamol with spike protein of SARS-CoV-2 | Results adopted |
| 167 | Roche, O’Connor, Murphy (2021) | Ivermectin in dermatology: why it ‘mite’ be useless against COVID-19 | Results adopted |
| 168 | Ravikirti et al. (2021) | Evaluation of Ivermectin as a Potential Treatment for Mild to Moderate COVID-19: A Double-Blind Randomized Placebo Controlled Trial in Eastern India | Results adopted |
| 169 | Ramíreza et al. (2021) | Is ivermectin ready to be part of a public health policy for COVID-19 prophylaxis? | Results adopted |
| 170 | Rakedzon et al. (2021) | From hydroxychloroquine to ivermectin: what are the anti-viral properties of anti-parasitic drugs to combat SARS-CoV-2? | Results adopted |
| 171 | Rajter et al. (2021) | Response: Standard Dose Ivermectin for COVID-19 | Results adopted |
| 172 | Qureshi et al. (2021) | Mechanistic insights into the inhibitory activity of FDA approved ivermectin against SARS-CoV-2: old drug with new implications | Results adopted |
| 173 | Quek et al. (2021) | Treatment of COVID-19: a review of current and prospective pharmacotherapies | Results adopted |
| 174 | Pedroso et al. (2021) | Self-prescribed Ivermectin use is associated with a lower rate of seroconversion in health care workers diagnosed with COVID, in a dose-dependent response | Results adopted |
| 175 | Ozer et al. (2021) | Effectiveness and safety of Ivermectin in COVID‐19 patients: A prospective study at a safety‐net hospital | Results adopted |
| 176 | Ohe, Furuya, Goudarz (2021) | Multidrug treatment for COVID-19 | Results adopted |
| 177 | Naik, Shakya (2021) | Therapeutic Strategies in the Management of COVID-19 | Results adopted |
| 178 | Morgenstern et al. (2021) | Ivermectin as a SARS-CoV-2 Pre-Exposure Prophylaxis Method in Healthcare Workers: A Propensity Score-Matched Retrospective Cohort Study | Results adopted |
| 179 | Mathachan, Sardana, Khurana (2021) | Current Use of Ivermectin in Dermatology, Tropical Medicine, and COVID-19: An Update on Pharmacology, Uses, Proven and Varied Proposed Mechanistic Action | Results adopted |
| 180 | Martínez (2021) | Electron Donor–Acceptor Capacity of Selected Pharmaceuticals against COVID-19 | Results adopted |
| 181 | Martin, Jans (2021) | Antivirals that target the host IMPα/β1-virus interface | Results adopted |
| 182 | Marik, Kory (2021) | Ivermectin, A Reanalysis of the Data | Results adopted |
| 183 | López-Medina et al. (2021) | Effect of Ivermectin on Time to Resolution of Symptoms Among Adults With Mild COVID-19 | Results adopted |
| 184 | Little; Cosetti (2021) | A Narrative Review of Pharmacologic Treatments for COVID-19: Safety Considerations and Ototoxicity | Results adopted |
| 185 | Lima-Morales et al. (2021) | Effectiveness of a multidrug therapy consisting of Ivermectin, Azithromycin, Montelukast, and Acetylsalicylic acid to prevent hospitalization and death among ambulatory COVID-19 cases in Tlaxcala, Mexico | Results adopted |
| 186 | Leung et al. (2021) | Ivermectin treatment for Strongyloides infection in patients with COVID-19 | Results adopted |
| 187 | Law et al. (2021) | Gastrointestinal and hepatic side effects of potential treatment for COVID-19 and vaccination in patients with chronic liver diseases | Results adopted |
| 188 | Kumar et al. (2021) | Moxidectin and Ivermectin Inhibit SARS-CoV-2 Replication in Vero E6 Cells but Not in Human Primary Bronchial Epithelial Cells | Results adopted |
| 189 | Kow, Hasan (2021) | Pitfalls in Reporting Sample Size Calculation Across Randomized Controlled Trials Involving Ivermectin for the treatment of COVID-19 | Results adopted |
| 190 | Kern et al. (2021) | Modeling of SARS-CoV-2 Treatment Effects for Informed Drug Repurposing | Results adopted |
| 191 | Kaur et al. (2021) | Ivermectin as a potential drug for treatment of COVID‐19: an in‐sync review with clinical and computational attributes | Results adopted |
| 192 | Jamir et al. (2021) | Determinants of Outcome Among Critically Ill Police Personnel With COVID-19: A Retrospective Observational Study From Andhra Pradesh, India | Results adopted |
| 193 | Jain et al. (2021) | Management of COVID-19 in patients with seizures: Mechanisms of action of potential COVID-19 drug treatments and consideration for potential drug-drug interactions with anti-seizure medications | Results adopted |
| 194 | Ismaila et al. (2021) | Therapeutic options for COVID-19: a quick review | Results adopted |
| 195 | Hagiya, Otsuka (2021) | Ivermectin for Coronavirus Disease 2019: Yet to Be Well Evaluated Before Clinical Use | Results adopted |
| 196 | H. Holzgreve (2021) | Ivermectin gegen Hakenwurm – und Coronavirus? | Results adopted |
| 197 | González-Paz et al. (2021) | Structural deformability induced in proteins of potential interest associated with COVID-19 by binding of homologues present in ivermectin: Comparative study based in elastic networks models | Results adopted |
| 198 | González-Paz et al. (2021) | Comparative study of the interaction of ivermectin with proteins of interest associated with SARS-CoV-2: A computational and biophysical approach | Results adopted |
| 199 | Francés-Monerris et al. (2021) | Microscopic interactions between ivermectin and key human and viral proteins involved in SARS-CoV-2 infection | Results adopted |
| 200 | Eweas, Alhossary, Abdel-Moneim (2021) | Molecular Docking Reveals Ivermectin and Remdesivir as Potential Repurposed Drugs Against SARS-CoV-2 | Results adopted |
| 201 | Errecalde et al. (2021) | Safety and Pharmacokinetic Assessments of a Novel Ivermectin Nasal Spray Formulation in a Pig Model | Results adopted |
| 202 | Duru et al. (2021) | Blocking the interactions between human ACE2 and coronavirus spike glycoprotein by selected drugs: a computational perspective | Results adopted |
| 203 | DiNicolantonio, Barroso-Aranda, McCarty (2021) | Anti-inflammatory activity ­of ­ivermectin­ in­ late-s­tage­ COVID-19­ may­ reflect­ activation ­of ­systemic­ glycine­ receptors | Results adopted |
| 204 | de Oliveiraa et al. (2021) | Repurposing approved drugs as inhibitors of SARS-CoV-2 S-protein from molecular modeling and virtual screening | Results adopted |
| 205 | de Melo et al. (2021) | Attenuation of clinical and immunological outcomes during SARS-CoV-2 infection by ivermectin Infection” | Results adopted |
| 206 | de Lima Machado et al. (2021) | Effectiveness and safety of ivermectin in the treatment of COVID-19: protocol for a systematic review and meta-analysis | Results adopted |
| 207 | Daoud et al. (2021) | Immunosuppression in kidney transplant recipients with COVID-19 infection – where do we stand and where are we heading? | Results adopted |
| 208 | Cobos-Camposa et al. (2021) | Potential use of ivermectin for the treatment and prophylaxis of SARS-CoV-2 infection | Results adopted |
| 209 | Chowdhury et al. (2021) | A secondary approach with conventional medicines and supplements to recuperate current COVID-19 status | Results adopted |
| 210 | Chosidow et al.. (2021) | Ivermectin as a potential treatment for COVID-19? | Results adopted |
| 211 | Chaudhry et al. (2021) | Role of ivermectin in patients hospitalized with COVID-19: a systematic review of literature | Results adopted |
| 212 | Chakraborty et al. (2021) | The Drug Repurposing for COVID-19 Clinical Trials Provide Very Effective Therapeutic Combinations: Lessons Learned From Major Clinical Studies | Results adopted |
| 213 | Chahla et al. (2021) | Intensive Treatment With Ivermectin and Iota-Carrageenan as Pre-exposure Prophylaxis for COVID-19 in Health Care Workers From Tucuman, Argentina | Results adopted |
| 214 | Cardwell et al. (2021) | Pharmacological interventions to prevent Covid‐19 disease: A rapid review | Results adopted |
| 215 | Cadegiani et al. (2021) | Early COVID-19 therapy with azithromycin plus nitazoxanide, ivermectin or hydroxychloroquine in outpatient settings significantly improved COVID-19 outcomes compared to known outcomes in untreated patients | Results adopted |
| 216 | Budnitz et al. (2021) | Increase in Outpatient Ivermectin Dispensing in the US During the COVID-19 Pandemic: A Cross-Sectional Analysis | Results adopted |
| 217 | Bryant, Lawrie, Fordham (2021) | Ivermectin for Prevention and Treatment of COVID-19 Infection: A Systematic Review, Meta-analysis, and Trial Sequential Analysis to Inform Clinical Guidelines. | Results adopted |
| 218 | Bousquet-Mélou et al. (2021) | A Large Impact of Obesity on the Disposition of Ivermectin, Moxidectin and Eprinomectin in a Canine Model: Relevance for COVID-19 Patients | Results adopted |
| 219 | Bhowmick et al. (2021) | Safety and Efficacy of Ivermectin and Doxycycline Monotherapy and in Combination in the Treatment of COVID‐19: A Scoping Review | Results adopted |
| 220 | Bhattacharyy et al. (2021) | COVID-19: morphology and mechanism of the SARS-CoV-2, global outbreak, medication, vaccines and future of the virus | Results adopted |
| 221 | Bestetti et al. (2021) | Pharmacological Treatment of Patients with Mild to Moderate COVID-19: A Comprehensive Review | Results adopted |
| 222 | Bernigaud et al. (2021) | Oral ivermectin for a scabies outbreak in a long-term care facility: potential value in preventing COVID-19 and associated mortality | Results adopted |
| 223 | Bermejo Galan et al. (2021) | Phase 2 randomized study on chloroquine, hydroxychloroquine or ivermectin in hospitalized patients with severe manifestations of SARS-CoV-2 infection | Results adopted |
| 224 | Bello (2021) | Elucidation of the inhibitory activity of ivermectin with host nuclear importin a and several SARS-CoV-2 targets | Results adopted |
| 225 | Behl et al. (2021) | CD147-spike protein interaction in COVID-19: Get the ball rolling with a novel receptor and therapeutic target | Results adopted |
| 226 | Behera et al. (2021) | Role of ivermectin in the prevention of SARS-CoV-2 infection among healthcare workers in India: A matched case-control study | Results adopted |
| 227 | Behera et al. (2021) | Prophylactic Role of Ivermectin in Severe Acute Respiratory Syndrome Coronavirus 2 Infection Among Healthcare Workers | Results adopted |
| 228 | Azeez et al. (2021) | Chemoprophylaxis against COVID-19 among health-care workers using Ivermectin in low- and middle-income countries: A systematic review and meta-analysis | Results adopted |
| 229 | Ayipo et al. (2021) | Pathomechanisms, therapeutic targets and potent inhibitors of some beta-coronaviruses from bench-to-bedside | Results adopted |
| 230 | Aslan, Akova (2021) | Current status of therapeutic alternatives for COVID-19: A narrative review | Results adopted |
| 231 | Aref et al. (2021) | Clinical, Biochemical and Molecular Evaluations of Ivermectin Mucoadhesive Nanosuspension Nasal Spray in Reducing Upper Respiratory Symptoms of Mild COVID-19 | Results adopted |
| 232 | Apaydın et al. (2021) | Small-molecule Antiviral Agents in Ongoing Clinical Trials for COVID-19 | Results adopted |
| 233 | Ambrus et al. (2021) | Interactions of anti‐COVID‐19 drug candidates with hepatic transporters may cause liver toxicity and affect pharmacokinetics | Results adopted |
| 234 | Alam et al. (2021) | Therapeutic Effectiveness and Safety of Repurposing Drugs for the Treatment of COVID-19: Position Standing in 2021 | Results adopted |
| 235 | Ahmed, Khan (2021) | A five-day course of ivermectin may reduce the duration of COVID-19 illness | Results adopted |
| 236 | Aherfi et al. (2021) | Drug repurposing against SARS-CoV-1, SARS-CoV-2 and MERS-CoV | Results adopted |
| 237 | Santin et al. (2021) | Ivermectin: a multifaceted drug of Nobel prize-honoured distinction with indicated efficacy against a new global scourge, COVID-19 | Results adopted |
| 238 | Chen et al. (2021) | Efficacy and safety of current medications for treating severe and non-severe COVID-19 patients: an updated network meta-analysis of randomized placebo-controlled trials | Results adopted |
| 239 | Zhang et al. (2021) | Efficacy of COVID-19 Treatments: A Bayesian Network Meta-Analysis of Randomized Controlled Trials | no reference |
| 240 | Yesilbag, Toker, Ates (2021) | Ivermectin also inhibits the replication of bovine respiratory viruses (BRSV, BPIV-3, BoHV-1, BCoV and BVDV) in vitro | no reference |
| 241 | Yanagida et al. (2021) | Comprehensive Cardiotoxicity Assessment of COVID-19 Treatments Using Human-Induced Pluripotent Stem Cell-Derived Cardiomyocytes | no reference |
| 242 | Wise, Scialli (2021) | Ivermectin for COVID-19: Concerns during pregnancy | no reference |
| 243 | Wang et al. (2021) | In vitro assays on the susceptibility of four species of nematophagous fungi to anthelmintics and chemical fungicides/antifungal drug | no reference |
| 244 | Thigpen (2021) | SARS CoV-2 (COVID-19) Current Pharmacotherapy for Mother and Infant | no reference |
| 245 | Suravajhala et al. (2021) | Molecular docking and dynamics studies of curcumin with COVID‐19 proteins | no reference |
| 246 | Subbarao (2021) | Cancer vs. SARS‐CoV‐2 induced inflammation, overlapping functions, and pharmacological targeting | no reference |
| 247 | Stylemans et al. (2021) | COVID-19–Associated Eosinopenia in a Patient With Chronic Eosinophilia Due to Chronic Strongyloidiasis | no reference |
| 248 | Stokel-Walker (2021) | Ivermectin buyers clubs | no reference |
| 249 | Simsek et al. (2021) | An update of anti-viral treatment of COVID-19 | no reference |
| 250 | Shah, Hariharan, Chawla (2021) | Common anti‐COVID‐19 drugs and their anticipated interaction with anesthetic agents | no reference |
| 251 | Samajdar et al. (2021) | Ivermectin and Hydroxychloroquine for Chemo-Prophylaxis of COVID-19: A Questionnaire Survey of Perception and Prescribing Practice of Physicians vis-a-vis Outcomes | no reference |
| 252 | Romanowski et al. (2021) | The in vitro Evaluation of the Activity of COVID-19 Antiviral Drugs Against Adenovirus | no reference |
| 253 | Rein (2021) | Harnessing autophagy to fight SARS-CoV-2: An update in view of recent drug development efforts | no reference |
| 254 | Quincho-Lopez et al. (2021) | Self-medication practices to prevent or manage COVID-19: A systematic review | no reference |
| 255 | Ortega-Guillén, Meneses, Coila (2021) | Remarks About Retrospective Analysis of Ivermectin Effectiveness on Coronavirus Disease 2019 (ICON Study) | no reference |
| 256 | Olivera (2021) | Dexamethasone and COVID-19: Strategies in Low- and Middle-Income Countries to Tackle Steroid-Related Strongyloides Hyperinfection | no reference |
| 257 | Okumus et al. (2021) | Evaluation of the effectiveness and safety of adding ivermectin to treatment in severe COVID-19 patients | no reference |
| 258 | Okogbenin et al. (2021) | Clinical Characteristics, Treatment Modalities and Outcome of Coronavirus Disease 2019 Patients Treated at ThisDay Dome Isolation and Treatment Centre, Federal Capital Territory Abuja, Nigeria | no reference |
| 259 | O’Higgins et al. (2021) | Off‐label use of ivermectin for COVID‐19: Are there any neuropsychiatric effects to be aware of? | no reference |
| 260 | Nunes et al. (2021) | Antivirals virtual screening to SARS-CoV-2 non-structural proteins | no reference |
| 261 | Nogrady (2021) | I hope you die': how the COVID pandemic unleashed attacks on scientists | no reference |
| 262 | Ngoa et al. (2021) | The time to offer treatments for COVID-19 | no reference |
| 263 | Murchu et al. (2021) | Interventions in an Ambulatory Setting to Prevent Progression to Severe Disease in Patients With COVID-19: A Systematic Review | no reference |
| 264 | Misra et al (2021) | Formulating Hypotheses for Different Study Designs | no reference |
| 265 | Mansour et al (2021) | Safety of inhaled ivermectin as a repurposed direct drug for treatment of COVID-19: A preclinical tolerance study | no reference |
| 266 | Kow et al. (2021) | The association between the use of ivermectin and mortality in patients with COVID‐19: a meta‐analysis | no reference |
| 267 | Kifle, Ayele, Enyew (2021) | Drug Repurposing Approach, Potential Drugs, and Novel Drug Targets for COVID-19 Treatment | no reference |
| 268 | Khodavirdipour et al. (2021) | Potential of CRISPR/Cas13 System in Treatment and Diagnosis of COVID-19 | no reference |
| 269 | Kamran et al. (2021) | SARS-CoV-2 infection pattern, transmission and treatment: Multi-center study in low to middle-income districts hospitals in Punjab, Pakistan | no reference |
| 270 | Jasuja et al. (2021) | COVID-19 Infection Clinical Profile, Management, Outcome, and Antibody Response in Kidney Transplant Recipients: A Single Centre Experience | no reference |
| 271 | Jagiasi et al. (2021) | Variation in therapeutic strategies for the management of severe COVID-19 in India: A nationwide cross-sectional survey | no reference |
| 272 | Hossein et al. (2021) | The efficacy and safety of Ivermectin in patients with mild and moderate COVID-19: A structured summary of a study protocol for a randomized controlled trial | no reference |
| 273 | Hamley et al. (2021) | What does the COVID-19 pandemic mean for the next decade of onchocerciasis control and elimination? | no reference |
| 274 | Giannattasio et al (2021) | Concomitant SARS-CoV-2 infection and crusted scabies in a 4-month infant | no reference |
| 275 | Garcia et al. (2021) | Randomized clinical trial to compare the efficacy of ivermectin versus placebo to negativize nasopharyngeal PCR in patients with early COVID-19 in Peru (SAINT-Peru): a structured summary of a study protocol for randomized controlled trial | no reference |
| 276 | Fricke-Galindo, Falfán-Valencia (2021) | Pharmacogenetics Approach for the Improvement of COVID-19 Treatment | no reference |
| 277 | Ferreira et al. (2021) | Outcomes associated with Hydroxychloroquine and Ivermectin in hospitalized patients with COVID-19: a single-center experience | no reference |
| 278 | Elekofehinti et al. (2021) | Identification of Main Protease of Coronavirus SARS-CoV-2 (M pro) Inhibitors from Melissa officinalis | no reference |
| 279 | Devi et al. (2021) | Deep vein thrombosis with pulmonary thromboembolism in a case of severe COVID-19 pneumonia | no reference |
| 280 | Bukanova et al. (2021) | Antiviral Drug Ivermectin at Nanomolar Concentrations Inhibits Glycine-Induced Chloride Current in Rat Hippocampal Neurons | no reference |
| 281 | Bryant et al. (2021) | Re: commentary by Rothrock et al. "Meta-analysis did not establish improved mortality with ivermectin use in COVID-19" | no reference |
| 282 | Bhorat, Bhorat (2021) | A qualitative analysis of seven ivermectin formulations in South Africa | no reference |
| 283 | Bhaskar (2021) | A Global Early Empirical Therapy Randomized Study Design for Ivermectin in SARS-CoV-2 Infection | no reference |
| 284 | Bartoszko et al. (2021) | Prophylaxis against covid-19: living systematic review and network meta-analysis | no reference |
| 285 | Badary (2021) | Pharmacogenomics and COVID-19: clinical implications of human genome interactions with repurposed drugs | no reference |
| 286 | Ashraf et al. (2021) | Anti-COVID property of subcutaneous ivermectin in synergy with zinc among midlife moderately symptomatic patients: a structured summary of a study protocol for a randomised controlled trial | no reference |
| 287 | Arouche et al. (2021) | Molecular Docking of Azithromycin, Ritonavir, Lopinavir, Oseltamivir, Ivermectin and Heparin Interacting with Coronavirus Disease 2019 Main and Severe Acute Respiratory Syndrome Coronavirus-2 3C-Like Proteases | no reference |
| 288 | Arévalo et al. (2021) | Ivermectin reduces in vivo coronavirus infection in a mouse experimental model | no reference |
| 289 | Anyaypoma-Ocón et al. (2021) | Factors associated with COVID-19 lethality in a hospital in the Cajamarca region in Peru | no reference |
| 290 | Amazigo et al. (2021) | Community-directed distributors-The "foot soldiers" in the fight to control and eliminate neglected tropical diseases | no reference |
| 291 | Ahsan et al. (2021) | Clinical Variants, Characteristics, and Outcomes Among COVID-19 Patients: A Case Series Analysis at a Tertiary Care Hospital in Karachi, Pakistan | no reference |
| 292 | Agarwal et al. (2021) | Update to living WHO guideline on drugs for covid-19 | no reference |
| 293 | Shukla, Misra (2022) | Antimicrobials in COVID-19: strategies for treating a COVID-19 pandemic | Results criticized |
| 294 | Van Scoy et al. (2022) | A mixed methods study exploring requests for unproven COVID therapies such as ivermectin and healthcare distrust in the rural South | Results criticized |
| 295 | Diaz et al. (2022) | The Politicization of Ivermectin Tweets During the COVID-19 Pandemic | Results criticized |
| 296 | Onyeaka et al. (2022) | The use of Ivermectin for the treatment of COVID-19: Panacea or enigma? | Results criticized |
| 297 | Barac et al. (2022) | Inappropriate use of ivermectin during the COVID-19 pandemic: primum non nocere! | Results criticized |
| 298 | Alibudbud (2022) | A Case of Pharmaceutical Messianism Amidst the COVID-19 Pandemic: An Infodemiological Study of Ivermectin in the Philippines | Results criticized |
| 299 | Rezai et al. (2022) | Non-effectiveness of Ivermectin on Inpatients and Outpatients With COVID-19; Results of Two Randomized, Double-Blinded, Placebo-Controlled Clinical Trials | Results criticized |
| 300 | Shafiee et al. (2022) | Ivermectin under scrutiny: a systematic review and meta-analysis of efficacy and possible sources of controversies in COVID-19 patients | Results criticized |
| 301 | Bazil et al. (2022) | COVID-19 update: NIH recommends against ivermectin | Results criticized |
| 302 | Angkasekwinai et al.(2022) | Safety and Efficacy of Ivermectin for the Prevention and Treatment of COVID-19: A Double-Blinded Randomized Placebo-Controlled Study | Results criticized |
| 303 | Long et al.(2022) | Clinical update on COVID-19 for the emergency and critical care clinician: Medical management | Results criticized |
| 304 | Nino-Orrego et al.(2022) | Prescription for COVID-19 by non-medical professionals during the pandemic in Colombia: a cross-sectional study | Results criticized |
| 305 | Kaduszkiewicz et al.(2022) | Recommendations for the Outpatient Drug Treatment of Patients With COVID-19 | Results criticized |
| 306 | Zhenga et al.(2022) | Red blood cell-hitchhiking mediated pulmonary delivery of ivermectin: Effects of nanoparticle properties | Results criticized |
| 307 | Reis et al. (2022) | Effect of Early Treatment with Ivermectin among Patients with Covid-19 | Results criticized |
| 308 | Tedeschi, Ventura (2022) | Viral Load Reduction and High-Dose Ivermectin in Early Treatment: A Reappraisal | Results criticized |
| 309 | Johnson-Arbor (2022) | Ivermectin: a mini-review | Results criticized |
| 310 | Chary et al. (2022) | COVID-19 Therapeutics: Use, Mechanism of Action, and Toxicity (Xenobiotics) | Results criticized |
| 311 | Chiu et al. (2022) | Safety profile of COVID-19 drugs in a real clinical setting | Results criticized |
| 312 | Cardwell et al. (2022) | Pharmacological interventions to prevent Covid-19 disease: A rapid review | Results criticized |
| 313 | Skarzynska et al. (2022) | Adverse Audio-Vestibular Effects of Drugs and Vaccines Used in the Treatment and Prevention of COVID-19: A Review | Results criticized |
| 314 | Heilmann et al. (2022) | A VSV-based assay quantifies coronavirus Mpro/3CLpro/Nsp5 main protease activity and chemical inhibition | Results criticized |
| 315 | Hill et al.(2022) | Ivermectin for the prevention of COVID-19: addressing potential bias and medical fraud | Results criticized |
| 316 | Baracaldo-Santamaría et al. (2022) | Drug safety of frequently used drugs and substances for self-medication in COVID-19 | Results criticized |
| 317 | Nadeem et al.(2022) | Development of Evidence-Based COVID-19 Management Guidelines for Local Context: The Methodological Challenges | Results criticized |
| 318 | Meyerowitz-Katz et al.(2022) | Unethical studies of ivermectin for covid-19 | Results criticized |
| 319 | Delandre et al.(2022) | Antiviral Activity of Repurposing Ivermectin against a Panel of 30 Clinical SARS-CoV-2 Strains Belonging to 14 Variants | Results criticized |
| 320 | Taccone, Hites, Dauby (2022) | From hydroxychloroquine to ivermectin: how unproven "cures" can go viral | Results criticized |
| 321 | Deng et al.(2022) | Safety cannot justify the use of ivermectin for the management of COVID-19 | Results criticized |
| 322 | O’Mathúna (2022) | Ivermectin and the Integrity of Healthcare Evidence During COVID-19 | Results criticized |
| 323 | Galkina et al.(2022) | Ivermectin Affects Neutrophil-Induced Inflammation through Inhibition of Hydroxylysine but Stimulation of Cathepsin G and Phenylalanine Secretion | Results criticized |
| 324 | Duverger, Herlem, Picaud (2022) | Nanovectorization of Ivermectin to avoid overdose of drugs | Results criticized |
| 325 | Madhavi et al.(2022) | Ivermectin in COVID-19 Management: What is the Current Evidence? | Results criticized |
| 326 | O'Malley (2022) | Ivermectin: 21st Century "Snake Oil" or Safe & Effective for COVID-19? | Results criticized |
| 327 | Šín, Kubiska (2022) | Death from COVID-19 of a 57-year-old man refusing medical care and self-medicating with ivermectin | Results criticized |
| 328 | De Forni et al. (2022) | Synergistic drug combinations designed to fully suppress SARS-CoV-2 in the lung of COVID-19 patients | Results criticized |
| 329 | Elshafie et al. (2022) | Ivermectin role in COVID-19 treatment (IRICT): single-center, adaptive, randomized, double-blind, placebo-controlled, clinical trial | Results criticized |
| 330 | Castillejos-López et al. (2022) | Ivermectin: A Controversial Focal Point during the COVID-19 Pandemic | Results criticized |
| 331 | Deng et al. (2022) | Caution should be exercised when assessing ivermectin for the treatment of COVID-19 in systematic reviews | Results criticized |
| 332 | Na-Bangchang et al. (2022) | Perspective: repurposed drugs for COVID-19 | Results criticized |
| 333 | Awad et al.(2022) | Repurposing Potential of the Antiparasitic Agent Ivermectin for the Treatment and/or Prophylaxis of COVID-19 | Results criticized |
| 334 | Barati et al. (2022) | Potential therapeutic effects of Ivermectin in COVID-19 | Results criticized |
| 335 | Farah et al. (2022) | Ivermectin associated adverse events in the treatment and prevention of COVID-19 reported to the FACT pharmacovigilance project | Results criticized |
| 336 | Marcolino et al. (2022) | Systematic review and meta-analysis of ivermectin for treatment of COVID-19: evidence beyond the hype | Results criticized |
| 337 | Maheshwari, Roy (2022) | In search for a panacea for coronavirus disease-19: Analysis of ongoing clinical trials for the management of coronavirus disease-19 pandemic in India | Results criticized |
| 338 | Bryant et al.(2022) | Re: Expression of Concern for Bryant A, Lawrie TA, Dowswell T, Fordham EJ, Mitchell S, Hill SR, Tham TC. Ivermectin for Prevention and Treatment of COVID-19 Infection. Am J Ther. 2022;29:e232 | Results adopted |
| 339 | Yang, Shen, Hou (2022) | Is Ivermectin Effective in Treating COVID-19? | Results adopted |
| 340 | Goyal et al. (2022) | Ivermectin-Induced Acute Psychosis in Patients Infected With COVID-19 Pneumonia | Results adopted |
| 341 | Popp et al. (2022) | Ivermectin for preventing and treating COVID‐19 | Results adopted |
| 342 | Jitobaom et al. (2022) | Synergistic anti-SARS-CoV-2 activity of repurposed anti-parasitic drug combinations | Results adopted |
| 343 | Schaffer et al.(2022) | Changes in dispensing of medicines proposed for re-purposing in the first year of the COVID-19 pandemic in Australia | Results adopted |
| 344 | Prathapan (2022) | A determination of pan-pathogen antimicrobials? | Results adopted |
| 345 | Fuentes-Gonzalez et al.(2022) | Outpatient prescription patterns of COVID-19 drugs in the metropolitan area of Mexico City | Results adopted |
| 346 | Sezer et al.(2022) | A review on drug repurposing in COVID-19: from antiviral drugs to herbal alternatives | Results adopted |
| 347 | Marquesa et al.(2022) | Ivermectin as a possible treatment for COVID-19: a review of the 2022 protocols | Results adopted |
| 348 | Albariqia et al.(2022) | Pharmacokinetics and safety of inhaled ivermectin in mice as a potential COVID-19 treatment | Results adopted |
| 349 | Sharmaa et al.(2022) | Combined therapy with ivermectin and doxycycline can effectively alleviate the cytokine storm of COVID-19 infection amid vaccination drive: A narrative review | Results adopted |
| 350 | Ho et al. (2022) | COVID-19 and the promise of small molecule therapeutics: Are there lessons to be learnt? | Results adopted |
| 351 | Molnar et al. (2022) | Ivermectin in COVID-19: The Case for a Moratorium on Prescriptions | Results adopted |
| 352 | Mir et al.(2022) | Immune-related therapeutics: an update on antiviral drugs and vaccines to tackle the COVID-19 pandemic | Results adopted |
| 353 | McCarthy (2022) | Current and emerging immunomodulators for treatment of SARS-CoV2 infection (COVID-19) | Results adopted |
| 354 | Schellack et al.(2022) | Social Media and COVID-19—Perceptions and Public Deceptions of Ivermectin, Colchicine and Hydroxychloroquine: Lessons for Future Pandemics | Results adopted |
| 355 | Foo et al.(2022) | Ivermectin Does Not Protect against SARS-CoV-2 Infection in the Syrian Hamster Model | Results adopted |
| 356 | Liu et al.(2022) | Genome-wide analyses reveal the detrimental impacts of SARS-CoV-2 viral gene Orf9c on human pluripotent stem cell-derived cardiomyocytes | Results adopted |
| 357 | Gonzalez et al. (2022) | Efficacy and Safety of Ivermectin and Hydroxychloroquine in Patients with Severe COVID-19: A Randomized Controlled Trial | Results adopted |
| 358 | Schwart (2022) | Does ivermectin have a place in the treatment of mild Covid-19? | Results adopted |
| 359 | Jeffreys et al.(2022) | Remdesivir-ivermectin combination displays synergistic interaction with improved in vitro activity against SARS-CoV-2 | Results adopted |
| 360 | Oyefabi et al. (2022) | Comparison of the Ivermectin and Lopinavir/Ritonavir Treatment Outcomes among COVID-19 Mild to Moderate Cases in Kaduna State | Results adopted |
| 361 | Ohe (2022) | Multi-drug Treatment for COVID-19-induced Acute Respiratory Distress Syndrome | Results adopted |
| 362 | Alejandro Mayer et al.(2022) | Safety and Efficacy of a MEURI Program for the Use of High Dose Ivermectin in COVID-19 Patients | Results adopted |
| 363 | Boretti (2022) | Zinc augments the antiviral potential of HCQ/CQ and ivermectin to reduce the risks of more serious outcomes from COVID-19 infection | Results adopted |
| 364 | Shirazi et al. (2022) | Repurposing the drug, ivermectin, in COVID-19: toxicological points of view | Results adopted |
| 365 | Hill et al. (2022) | Ivermectin for COVID-19: Addressing Potential Bias and Medical Fraud | Results adopted |
| 366 | Buonfrate et al. (2022) | High-dose ivermectin for early treatment of COVID-19 (COVER study): a randomised, double-blind, multicentre, phase II, dose-finding, proof-of-concept clinical trial | Results adopted |
| 367 | Zubair et al. (2022) | The effect of ivermectin on non-severe and severe COVID-19 disease and gender-based difference of its effectiveness | Results adopted |
| 368 | Kumar et al. (2022) | Moxidectin and Ivermectin Inhibit SARS-CoV-2 Replication in Vero E6 Cells but Not in Human Primary Bronchial Epithelial Cells | Results adopted |
| 369 | Babalola et al. (2022) | Ivermectin shows clinical benefits in mild to moderate COVID19: A randomised controlled double-blind, dose-response study in Lagos. | Results adopted |
| 370 | Oscanoa et al. (2022) | Hepatic disorders associated with the use of Ivermectin for SARS- CoV-2 infection in adults: a pharmacovigilance study in VigiBase | Results adopted |
| 371 | Porubcin et al. (2022) | Intravenous veterinary ivermectin in a COVID-19 patient causing neurotoxicity | Results adopted |
| 372 | de la Rocha et al. (2022) | Dynamics and binding affinity of nucleoside and non-nucleoside inhibitors with RdRp of SARS-CoV-2: a molecular screening, docking, and molecular dynamics simulation study | Results adopted |
| 373 | Samdani et al. (2022) | Targeting SARS-CoV-2 non-structural protein 13 via helicase-inhibitor-repurposing and non-structural protein 16 through pharmacophore-based screening | Results adopted |
| 374 | Farfán-Castillo et al. (2022) | Use of Ivermectin and Chlorine Dioxide for for COVID-19 Treatment and Prophylaxis in Peru: A Narrative Review | Results adopted |
| 375 | Kujur et al. (2022) | Effect of Ivermectin prophylaxis in prevention of COVID 19: Meta-analysis and systematic review | Results adopted |
| 376 | Hazan et al. (2022) | Letter in reply: more power to ivermectin multidrug therapy | Results adopted |
| 377 | Harza-Santiago et al. (2022) | Comment on: 'Effectiveness of ivermectin-based multidrug therapy in severely hypoxic, ambulatory COVID-19 patients' | Results adopted |
| 378 | Annie et al.(2022) | The Effect of Ivermectin on Cases of COVID-19 | Results adopted |
| 379 | Bello (2022) | Elucidation of the inhibitory activity of ivermectin with host nuclear importin a and several SARS-CoV-2 targets | Results adopted |
| 380 | Cheng et al.(2022) | Comparative efficacy and safety of pharmacological interventions for severe COVID-19 patients: An updated network meta-analysis of 48 randomized controlled trials | Results adopted |
| 381 | George et al. (2022) | Single Dose of Ivermectin is not Useful in Patients with Hematological Disorders and COVID-19 Illness: A Phase II B Open Labelled Randomized Controlled Trial | Results adopted |
| 382 | Albariqi et al. (2022) | Preparation and Characterization of Inhalable Ivermectin Powders as a Potential COVID-19 Therapy | Results adopted |
| 383 | Sarojvisut et al. (2022) | An Open Label Randomized Controlled Trial of Ivermectin Plus Favipiravir-Based Standard of Care versus Favipiravir-Based Standard of Care for Treatment of Moderate COVID-19 in Thailand | Results adopted |
| 384 | Qureshi et al. (2022) | Mechanistic insights into the inhibitory activity of FDA approved ivermectin against SARS-CoV-2: old drug with new implications | Results adopted |
| 385 | Zhanga et al. (2022) | In vitro evaluation of the impact of Covid-19 therapeutic agents on the hydrolysis of the antiviral prodrug remdesivir | Results adopted |
| 386 | Aref et al. (2022) | Possible Role of Ivermectin Mucoadhesive Nanosuspension Nasal Spray in Recovery of Post-COVID-19 Anosmia | Results adopted |
| 387 | Michel et al. (2022) | Understanding the widespread use of veterinary ivermectin for Chagas disease, underlying factors and implications for the COVID-19 pandemic: a convergent mixed-methods study | Results adopted |
| 388 | Mohseni et al (2022) | Potential limitations in systematic review studies assessing the effect of the main intervention for treatment/therapy of COVID-19 patients: An overview | Results adopted |
| 389 | Hu et al. (2022) | Ivermectin's Role in the Prevention of COVID-19: A Systematic Review and Meta-Analysis | Results adopted |
| 390 | Said et al. (2022) | Synthesis and greener pastures biological study of bis-thiadiazoles as potential Covid-19 drug candidates | Results adopted |
| 391 | Biber et al. (2022) | The effect of ivermectin on the viral load and culture viability in early treatment of nonhospitalized patients with mild COVID-19 - a double-blind, randomized placebo-controlled trial | Results adopted |
| 392 | Mirahmadizadeh et al. (2022) | Efficacy of single-dose and double-dose ivermectin early treatment in preventing progression to hospitalization in mild COVID-19: A multi-arm, parallel-group randomized, double-blind, placebo-controlled trial | Results adopted |
| 393 | Alsmadi (2022) | The investigation of the complex population-drug-drug interaction between ritonavir-boosted lopinavir and chloroquine or ivermectin using physiologically-based pharmacokinetic modeling | Results adopted |
| 394 | Barbash et al. (2022) | Adoption and Deadoption of Medications to Treat Hospitalized Patients With COVID-19 | no reference |
| 395 | Franczyk et al. (2022) | Will the Use of Pharmacogenetics Improve Treatment Efficiency in COVID-19? | no reference |
| 396 | Ogunyemi et al.(2022) | An internet-based cross-sectional study on infection control practices and drug use for COVID-19 prevention in Nigerian adults | no reference |
| 397 | Alian et al.(2022) | Mucormycosis, one month after recovery from COVID-19: A case report | no reference |
| 398 | Griffin et al.(2022) | Unexpected consequences of SARS-CoV-2 pandemic: scabies infestation | no reference |
| 399 | Kieliszek (2022) | Selenium in the Prevention of SARS‐CoV‐2 and Other Viruses | no reference |
| 400 | Parise et al.(2022) | Impact of COVID-19 therapy on hyperglycemia | no reference |
| 401 | Buonfrate, Rulli, Bisoffi (2022) | Reply to: Viral load reduction and high-dose ivermectin in early treatment: a reappraisal | no reference |
| 402 | Imran et al. (2022) | The Therapeutic and Prophylactic Potential of Quercetin against COVID-19: An Outlook on the Clinical Studies, Inventive Compositions, and Patent Literature | no reference |
| 403 | Ahmed et al.(2022) | Identification of host transcriptome-guided repurposable drugs for SARS-CoV-1 infections and their validation with SARS-CoV-2 infections by using the integrated bioinformatics approaches | no reference |
| 404 | Giannos et al.(2022) | Persistent Hiccups as an Atypical Presentation of SARS-CoV-2 Infection: A Systematic Review of Case Reports | no reference |
| 405 | Czeresnia, Weiss (2022) | Strongyloides stercoralis | no reference |
| 406 | Dicks, Deane,Grobbelaar (2022) | Could the COVID-19-Driven Increased Use of Ivermectin Lead to Incidents of Imbalanced Gut Microbiota and Dysbiosis? | no reference |
| 407 | Lim et al. (2022) | Efficacy of Ivermectin Treatment on Disease Progression Among Adults With Mild to Moderate COVID-19 and Comorbidities: The I-TECH Randomized Clinical Trial | no reference |
| 408 | Ocanha-Xavier, Xavier-Junior (2022) | Despite the use of ivermectin during the COVID pandemic, scabies keeps playing tricks with us | no reference |
| 409 | Ascencio-Montiel et al. (2022) | A Multimodal Strategy to Reduce the Risk of Hospitalization/death in Ambulatory Patients with COVID-19 | no reference |
| 410 | Okoye et al.(2022) | Self medication practices and its determinants in health care professionals during the coronavirus disease-2019 pandemic: cross-sectional study | no reference |
| 411 | Vasquez-Elera et al.(2022) | Self-medication in hospitalized patients with COVID-19: A cross-sectional study in northern Peru | no reference |
| 412 | Hernandez et al.(2022) | Reply to Banno et al and Padhi et al | no reference |
| 413 | Imran et al.(2022) | Development of Therapeutic and Prophylactic Zinc Compositions for Use against COVID-19: A Glimpse of the Trends, Inventions, and Patents | no reference |
| 414 | Ho et al.(2022) | Outpatient purchasing patterns of hydroxychloroquine and ivermectin in the USA and Canada during the COVID-19 pandemic: an interrupted time series analysis from 2016 to 2021 | no reference |
| 415 | Mosharaf et al.(2022) | Computational identification of host genomic biomarkers highlighting their functions, pathways and regulators that influence SARS-CoV-2 infections and drug repurposing | no reference |
| 416 | Valladales-Restrepo et al.(2022) | Prescription Patterns of Drugs Given to Hospitalized COVID-19 Patients: A Cross-Sectional Study in Colombia | no reference |
| 417 | Quiñones-LaverianoI et al. (2022) | Mortality and associated risk factors in patients hospitalized due to COVID-19 in a Peruvian reference hospital | no reference |
| 418 | Falavigna et al.(2022) | Brazilian guidelines for the treatment of outpatients with suspected or confirmed COVID-19. A joint guideline of the Brazilian Association of Emergency Medicine (ABRAMEDE), Brazilian Medical Association (AMB), Brazilian Society of Angiology and Vascular Surgery (SBACV), Brazilian Society of Geriatrics and Gerontology (SBGG), Brazilian Society of Infectious Diseases (SBI), Brazilian Society of Family and Community Medicine (SBFMC), and Brazilian Thoracic Society (SBPT) | no reference |
| 419 | Bitterman et al.(2022) | Comparison of Trials Using Ivermectin for COVID-19 Between Regions With High and Low Prevalence of Strongyloidiasis | no reference |
| 420 | Kalsar et al.(2022) | Coronavirus disease 2019 on routine testing in eclampsia: a case report | no reference |
| 421 | Hazan et al.(2022) | Effectiveness of ivermectin-based multidrug therapy in severely hypoxic, ambulatory COVID-19 patients | no reference |
| 422 | Giannattasio et al. (2022) | Concomitant SARS-CoV-2 infection and crusted scabies in a 4-month infant | no reference |
| 423 | Murchu et al.(2022) | Interventions in an Ambulatory Setting to Prevent Progression to Severe Disease in Patients With COVID-19: A Systematic Review | no reference |
| 424 | Dyer (2022) | Covid-19: Mexico City gave ivermectin kits to people with covid in “unethical” experiment | no reference |
| 425 | Behl et al. (2022) | CD147-spike protein interaction in COVID-19/ Get the ball rolling with a novel receptor and therapeutic target | no reference |
| 426 | Biswas et al. (2022) | Pharmacogenetics and Precision Medicine Approaches for the Improvement of COVID-19 Therapies | no reference |
| 427 | Manu (2022) | Expression of Concern for Bryant a, Lawrie TA, Dowswell T, Fordham EJ, Mitchell S, Hill SR, Tham TC. Ivermectin for Prevention and Treatment of COVID-19 Infection: A Systematic Review, Meta-Analysis, and Trial Sequential Analysis to Inform Clinical Guidelines. Am J Ther. 2021;28(4): e434-e460 | no reference |
| 428 | Paichitrojjana (2022) | Demodicosis Associated with Wearing a Face Mask: A Case Report | no reference |
| 429 | Manu (2022) | Expression of Concern for Kory P, Meduri GU, Varon J, Iglesias J, Marik PE. Review of the Emerging Evidence Demonstrating the Efficacy of Ivermectin in the Prophylaxis and Treatment of COVID-19. Am J Ther. 2021;28(3): e299-e318 | no reference |
| 430 | Rothrock et al. (2022) | Meta-Analyses Do Not Establish Improved Mortality With Ivermectin Use in COVID-19 | no reference |
| 431 | Bhinder et al. (2022) | Chronic Kidney Disease and COVID-19: Outcomes of hospitalised adults from a tertiary care centre in North India | no reference |
| 432 | Chua et al.(2022) | US Insurer Spending on Ivermectin Prescriptions for COVID-19 | no reference |
| 433 | Nahalka (2022) | Transcription of the Envelope Protein by 1-L Protein–RNA Recognition Code Leads to Genes/Proteins That Are Relevant to the SARS-CoV-2 Life Cycle and Pathogenesis | no reference |
| 434 | Reis, Mills (2022) | Ivermectin Treatment for Covid-19. Reply | no reference |
| 435 | Dutta et al. (2022) | Demand of COVID-19 medicines without prescription among community pharmacies in Jodhpur, India: Findings and implications | no reference |
| 436 | Rein (2022) | Harnessing autophagy to fight SARS‐CoV‐2: An update in view of recent drug development efforts | no reference |
| 437 | Furlan (2022) | Ivermectin Treatment for Covid-19 | no reference |
| 438 | Barkati, Greenaway, Libman (2022) | Strongyloidiasis in immunocompromised migrants to non-endemic countries in the era of COVID-19: What is the role for presumptive ivermectin? | no reference |
| 439 | Niaee et al. (2022) | Ivermectin-Induced Clinical Improvement and Alleviation of Significant Symptoms of COVID-19 Outpatients: A Cross-Sectional Study | no reference |
| 440 | Al-kuraishy et al. (2022) | Central Effects of Ivermectin in Alleviation of Covid-19-induced Dysauto- nomia | no reference |
| 441 | Falavigna et al. (2022) | Brazilian Guidelines for the pharmacological treatment of patients hospitalized with COVID-19: Joint guideline of Associação Brasileira de Medicina de Emergência, Associação de Medicina Intensiva Brasileira, Associação Médica Brasileira, Sociedade Brasileira de Angiologia e Cirurgia Vascular, Sociedade Brasileira de Infectologia, Sociedade Brasileira de Pneumologia e Tisiologia, Sociedade Brasileira de Reumatologia | no reference |
| 442 | Mejía, Jimenez (2022) | Ivermectin Treatment for Covid-19 | no reference |
| 443 | Akaslan, Mert, Kücük (2022) | Scabies increase during the COVID-19 pandemic: should we change our treatment strategy during the pandemic? | no reference |
| 444 | Calello, Kazzi, Stolbach (2022) | American College of Medical Toxicology (ACMT) Cautions Against Off-Label Prescribing of Ivermectin for the Prevention or Treatment of COVID-19 | no reference |
| 445 | Koçak, İpek (2022) | Electrochemical Detection of Ivermectin Used for the Treatment of COVID‐19 with Glutardialdehyde‐Modified Glassy Carbon Electrode | no reference |
| 446 | Barus, Gautier, Wabont (2022) | Ivermectin Treatment for Covid-19 | no reference |
| 447 | Bramante, Buse, Boulware (2022) | Trial of Metformin, Ivermectin, and Fluvoxamine for Covid-19. Reply | no reference |
| 448 | Shukla, Misra (2022) | Trial of Metformin, Ivermectin, and Fluvoxamine for Covid-19 | no reference |
| 449 | de la Rocha et al (2022) | Ivermectin compared with placebo in the clinical course in Mexican patients with asymptomatic and mild COVID-19: a randomized clinical trial | no reference |
| 450 | Boschi et al. (2022) | SARS-CoV-2 Spike Protein Induces Hemagglutination: Implications for COVID-19 Morbidities and Therapeutics and for Vaccine Adverse Effects | no reference |
| 451 | Ilangovan et al. (2022) | Current status of usage of ivermectin in the management of COVID | no reference |
| 452 | Hoang et al. (2022) | Characteristics of ivermectin toxicity in patients taking veterinary and human formulations for the prevention and treatment of COVID-19 | no reference |
| 453 | Soriano-Moreno et al. (2022) | Factors Associated With Drug Consumption Without Scientific Evidence in Patients With Mild COVID-19 in Peru | no reference |
| 454 | Polkinghorne, Branley (2022) | Medications for early treatment of COVID‐19 in Australia | no reference |
| 455 | Homer et al. (2022) | The National COVID‐19 Clinical Evidence Taskforce: pregnancy and perinatal guidelines | no reference |
| 456 | Manu (2022) | Ivermectin for COVID-19: The 2022 Update | no reference |
| 457 | Caira-Chuquineyraa et al. (2022) | Association between prehospital medication and fatal outcomes in a cohort of hospitalized patients due to coronavirus disease-2019 in a referral hospital in Peru | no reference |
| 458 | Campillo, Faillie (2022) | Adverse drug reactions associated with ivermectin use for COVID-19 reported in the WHO´s pharmacovigilance database | no reference |
| 459 | Montastruc (2022) | Adverse drug reactions and ivermectin in COVID-19 | no reference |
| 460 | Brady et al.(2022) | A guide to COVID-19 antiviral therapeutics: a summary and perspective of the antiviral weapons against SARS-CoV-2 infection | no reference |
| 461 | Luvira et al. (2022) | Strongyloides stercoralis: A Neglected but Fatal Parasite | no reference |
| 462 | Moraes et al. (2022) | COVID-19 incidence, severity, medication use, and vaccination among dentists: survey during the second wave in Brazil* | no reference |
| 463 | Chellasamy, Watson (2022) | Docking and molecular dynamics studies of human ezrin protein with a modelled SARS-CoV-2 endodomain and their interaction with potential invasion inhibitors | no reference |
| 464 | Stanford et al. (2022) | Evidence in decision-making in the context of COVID-19 in Latin America | no reference |
| 465 | Hua et al (2022) | Using Twitter data to understand public perceptions of approved versus off-label use for COVID-19-related medications | no reference |
| 466 | Schmidt et al. (2022) | The prevalence of onchocerciasis in Africa and Yemen, 2000-2018: a geospatial analysis | no reference |
| 467 | Chakravarty et al. (2022) | COVID-19-associated Mucormycosis: A clinico-epidemiological study | no reference |
| 468 | Choudhary et al. (2022) | Factors Associated With Severe Illness in Patients Aged <21 Years Hospitalized for COVID-19 | no reference |
| 469 | Schwalb et al. (2022) | COVID-19 in Latin America and the Caribbean: Two years of the pandemic | no reference |
| 470 | Kerr et al. (2022) | Regular Use of Ivermectin as Prophylaxis for COVID-19 Led Up to a 92% Reduction in COVID-19 Mortality Rate in a Dose-Response Manner: Results of a Prospective Observational Study of a Strictly Controlled Population of 88,012 Subjects | no reference |
| 471 | Watari et al. (2022) | Incidence of and Ivermectin Prescription Trends for COVID-19 in Japan | no reference |
| 472 | Manomaipiboon et al. (2022) | Efficacy and safety of ivermectin in the treatment of mild to moderate COVID-19 infection: a randomized, double-blind, placebo-controlled trial | no reference |
| 473 | Coffin et al.(2022) | Putative COVID-19 therapies imatinib, lopinavir, ritonavir, and ivermectin cause hair cell damage: A targeted screen in the zebrafish lateral line | no reference |
| 474 | Amaeze et al. (2022) | Community Pharmacists' Services during the COVID-19 Pandemic: A Case Study of Lagos State, Nigeria | no reference |
| 475 | Bramante et al. (2022) | Randomized Trial of Metformin, Ivermectin, and Fluvoxamine for Covid-19 | no reference |
| 476 | Fazio et al.(2022) | The Problem of Home Therapy during COVID-19 Pandemic in Italy: Government Guidelines versus Freedom of Cure? | no reference |
| 477 | Lorente et al.(2022) | Ivermectin exposures reported to the Poisons Information Helpline in South Africa during the COVID-19 pandemic | no reference |
| 478 | Romaní et al.(2022) | Association between the Use of Antibiotics and the Development of Acute Renal Injury in Patients Hospitalized for COVID-19 in a Hospital in the Peruvian Amazon | no reference |
| 479 | Alvarado et al. (2022) | Interaction of the new inhibitor paxlovid (PF-07321332) and ivermectin with the monomer of the main protease SARS-CoV-2/ A volumetric study based on molecular dynamics, elastic networks, classical thermodynamics and SPT | no reference |
| 480 | Bomze et al.(2022) | Severe cutaneous adverse reactions associated with systemic ivermectin: A pharmacovigilance analysis | no reference |
| 481 | Shaw et al. (2022) | COVID-19 Misinformation and Social Network Crowdfunding: Cross-sectional Study of Alternative Treatments and Antivaccine Mandates | no reference |
| 482 | Hentschke-Lopes et al. (2022) | Sales of "COVID kit" drugs and adverse drug reactions reported by the Brazilian Health Regulatory Agency | no reference |
| 483 | Saha et al. (2022) | Manipulation of Spray-Drying Conditions to Develop an Inhalable Ivermectin Dry Powder | no reference |
| 484 | Cosentino et al. (2022) | Early Outpatient Treatment of COVID-19: A Retrospective Analysis of 392 Cases in Italy | no reference |

*Supplemental Table 8* The table shows a list of all publications 2020 – 2022 and their opinion on the publication of Caly et al.
